# Supplementary material for: Biomarker-guided responses in patient-derived organoids predict effective therapies in breast cancer
Source: Cell Rep Med. 2026 Aug 6;7(8):102973. doi: 10.1016/j.xcrm.2026.102973 (PMC13522806; doi:10.1016/j.xcrm.2026.102973)

Cell Reports Medicine, Volume *7*

Supplemental information

Biomarker-guided responses in patient-derived organoids predict effective therapies in breast cancer

Tam Binh V. Bui, Denise M. Wolf, Michael C. Bruck, Kaitlin Moore, Jessica Lien, Sarah D. W. Choi, Shruti Warhadpande, Amirabbas Parizadeh, Deborah Dillon, Beth Overmoyer, Filipa Lynce, Lamorna Brown-Swigart, Gillian L. Hirst, Christina Yau, I-SPY2 Investigators, Isaac J. Nijman, Boudewijn M.T. Burgering, Isaac S. Harris, Laura J. Esserman, Laura J. van `t Veer, and Jennifer M. Rosenbluth

Figure S1

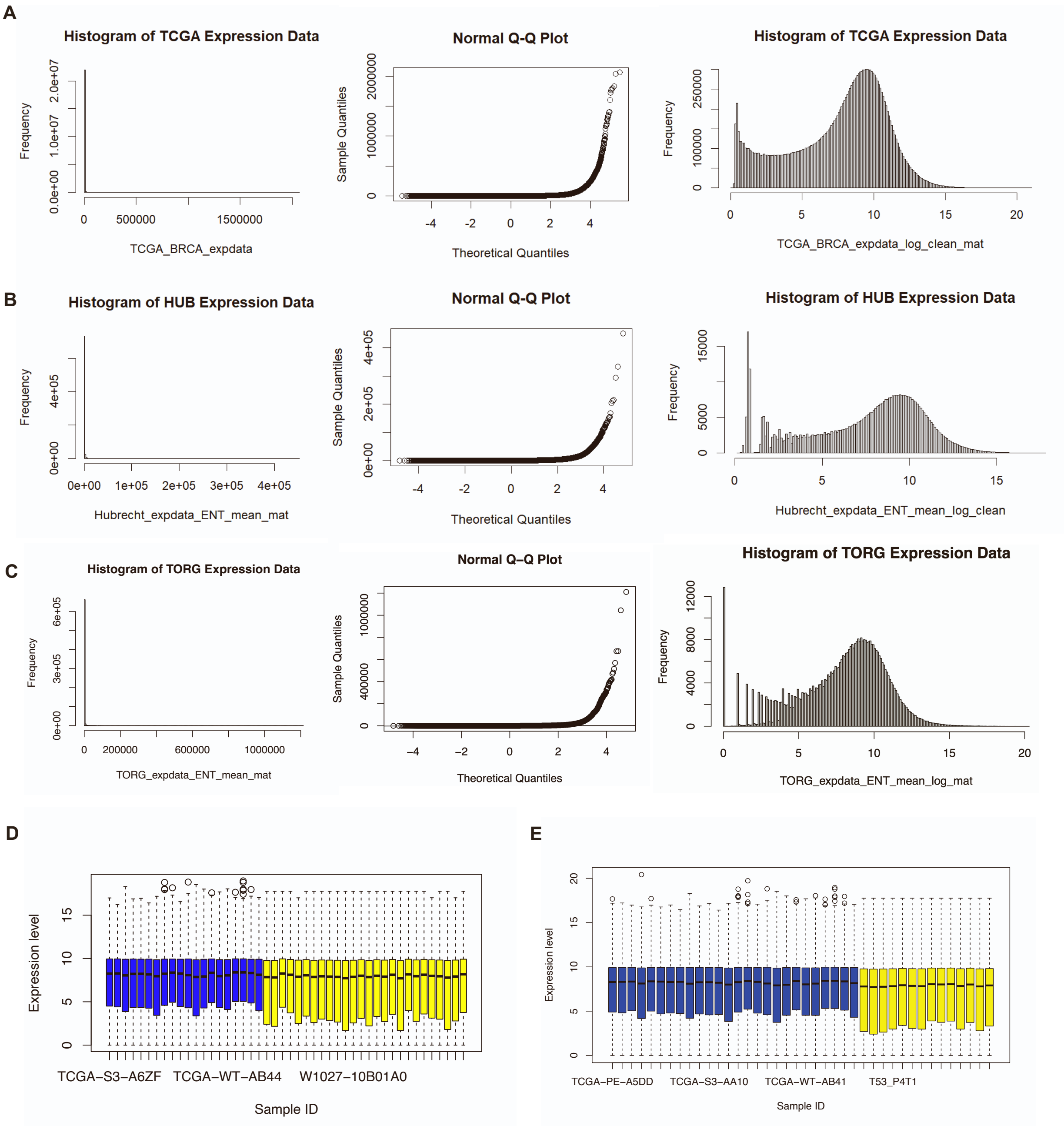

A
Histogram of TCGA Expression Data
Frequency
TCGA_BRCA_expdata
Normal Q-Q Plot
Sample Quantiles
Theoretical Quantiles
Histogram of TCGA Expression Data
TCGA_BRCA_expdata_log_clean_mat
B
Histogram of HUB Expression Data
Hubrecht_expdata_ENT_mean_mat
Normal Q-Q Plot
Histogram of HUB Expression Data
Hubrecht_expdata_ENT_mean_log_clean
C
Histogram of TORG Expression Data
TORG_expdata_ENT_mean_mat
Normal Q–Q Plot
Histogram of TORG Expression Data
TORG_expdata_ENT_mean_log_mat
D
Expression level
TCGA–S3–A6ZF
TCGA–WT–AB44
W1027–10B01A0
Sample ID
E
Expression level
TCGA–PE–A5DD
TCGA–S3–AA10
TCGA–WT–AB41
T53_P4T1
Sample ID

Figure S1. Quality controls for the merge/integration of organoid bulk RNA-Seq and TCGA tissue bulk RNA-Seq data. Q-Q plot = Quantile-Quantile plot. TCGA = The Cancer Genome Atlas. Related to Figure 1 and Figure 3.

A. Gene expression data from TCGA published breast cancer tissues. Left to right: histogram of data before log-transformation, Q-Q plot of data before log-transformation, and histogram of log-transformed expression data.

B. Gene expression data from HUB biobank (Hubrecht Institute) organoids. Left to right: histogram of data before log-transformation, Q-Q plot of data before log-transformation, and histogram of log-transformed expression data.

C. Gene expression data from TNBC TORG (UCSF triple-negative breast cancer organoids). Left to right: histogram of data before log-transformation, Q-Q plot of data before log-transformation, and histogram of log-transformed expression data.

D. Boxplots showing expression levels for a representative subset of the TCGA dataset (blue bars), and all HUB organoid samples (n=26; yellow bars) after quantile-normalization of organoid data to TCGA data and merge of the data. The boxplots illustrate comparable expression levels within samples of both datasets after transformation of the data.

E. Boxplots showing expression levels for a representative subset of the TCGA dataset (blue bars), and all TORG TNBC organoid samples (n=14; yellow bars) after quantile-normalization of organoid data to TCGA data and merge of the data. The boxplots illustrate comparable expression levels within samples of both datasets after transformation of the data.

Figure S2

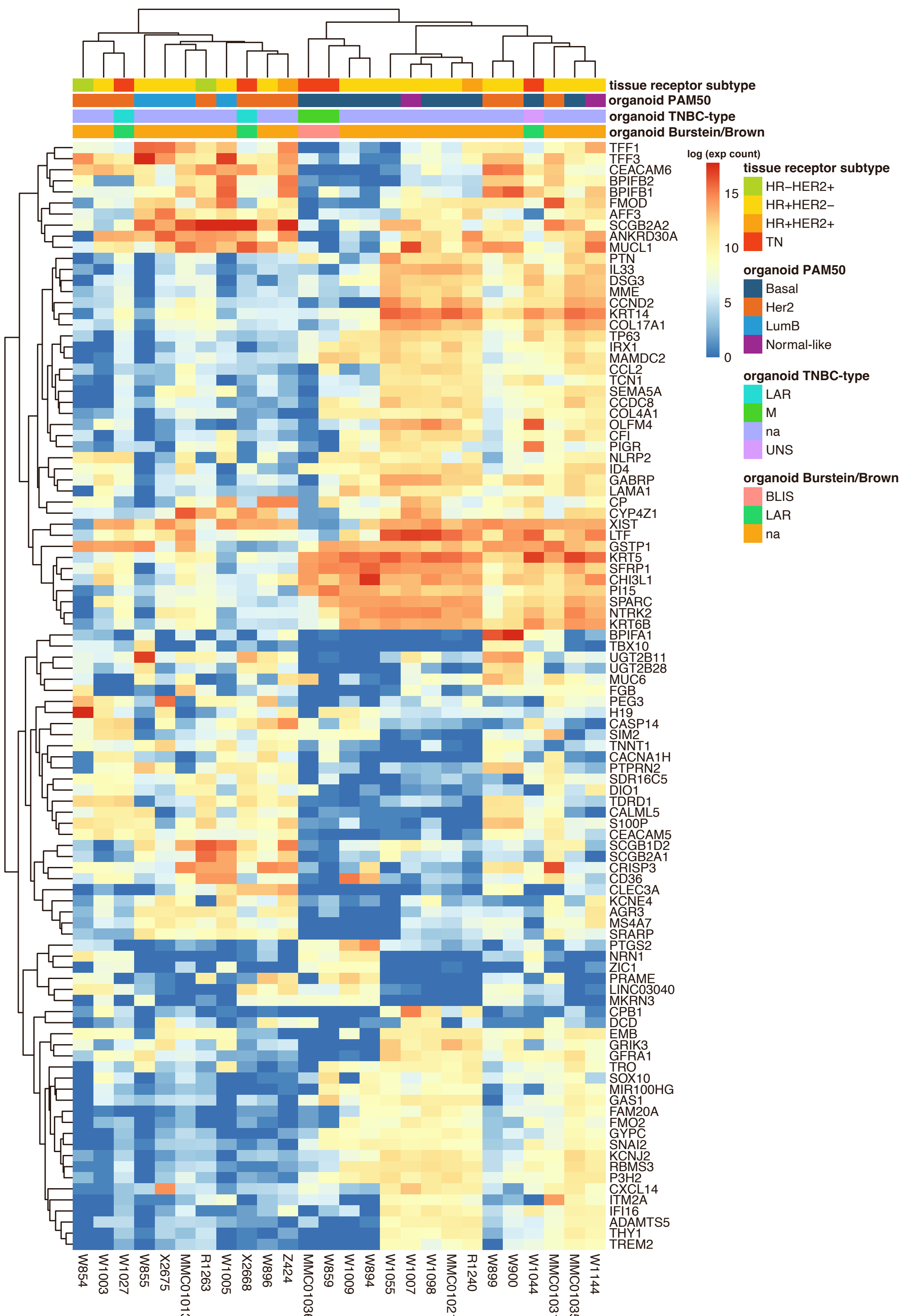
tissue receptor subtype
organoid PAM50
organoid TNBC-type
organoid Burstein/Brown
log (exp count)
15
10
5
0
tissue receptor subtype
HR−HER2+
HR+HER2−
HR+HER2+
TN
organoid PAM50
Basal
Her2
LumB
Normal-like
organoid TNBC-type
LAR
M
na
UNS
organoid Burstein/Brown
BLIS
LAR
na
TFF1
TFF3
CEACAM6
BPIFB2
BPIFB1
FMOD
AFF3
SCGB2A2
ANKRD30A
MUCL1
PTN
IL33
DSG3
MME
CCND2
KRT14
COL17A1
TP63
IRX1
MAMDC2
CCL2
TCN1
SEMA5A
CCDC8
COL4A1
OLFM4
CFI
PIGR
NLRP2
ID4
GABRP
LAMA1
CP
CYP4Z1
XIST
LTF
GSTP1
KRT5
SFRP1
CHI3L1
PI15
SPARC
NTRK2
KRT6B
BPIFA1
TBX10
UGT2B11
UGT2B28
MUC6
FGB
PEG3
H19
CASP14
SIM2
TNNT1
CACNA1H
PTPRN2
SDR16C5
DIO1
TDRD1
CALML5
S100P
CEACAM5
SCGB1D2
SCGB2A1
CRISP3
CD36
CLEC3A
KCNE4
AGR3
MS4A7
SRARP
PTGS2
NRN1
ZIC1
PRAME
LINC03040
MKRN3
CPB1
DCD
EMB
GRIK3
GFRA1
TRO
SOX10
MIR100HG
GAS1
FAM20A
FMO2
GYPC
SNAI2
KCNJ2
RBMS3
P3H2
CXCL14
ITM2A
IFI16
ADAMTS5
THY1
TREM2
W854
W1003
W1027
W855
X2675
MMC01013
R1263
W1005
X2668
W896
Z424
MMC01036
W859
W1009
W894
W1055
W1007
W1098
MMC01021
R1240
W899
W900
W1044
MMC01031
MMC01035
W1144

Figure S2. Unsupervised clustering of HUB biobank organoids based on the top 100 most differentially expressed genes. Annotations for each of the indicated subtype classification schemas are shown. BLIS = basal-like immunosuppressed. Exp count = expected count (RSEM). HR = hormone receptor. LAR = luminal androgen receptor. LumB = luminal B. M = mesenchymal. na = not applicable (sample is not triple-negative). TN = triple-negative. UNS = a subtype could not be selected by the algorithm. Related to Figure 1.

Figure S3

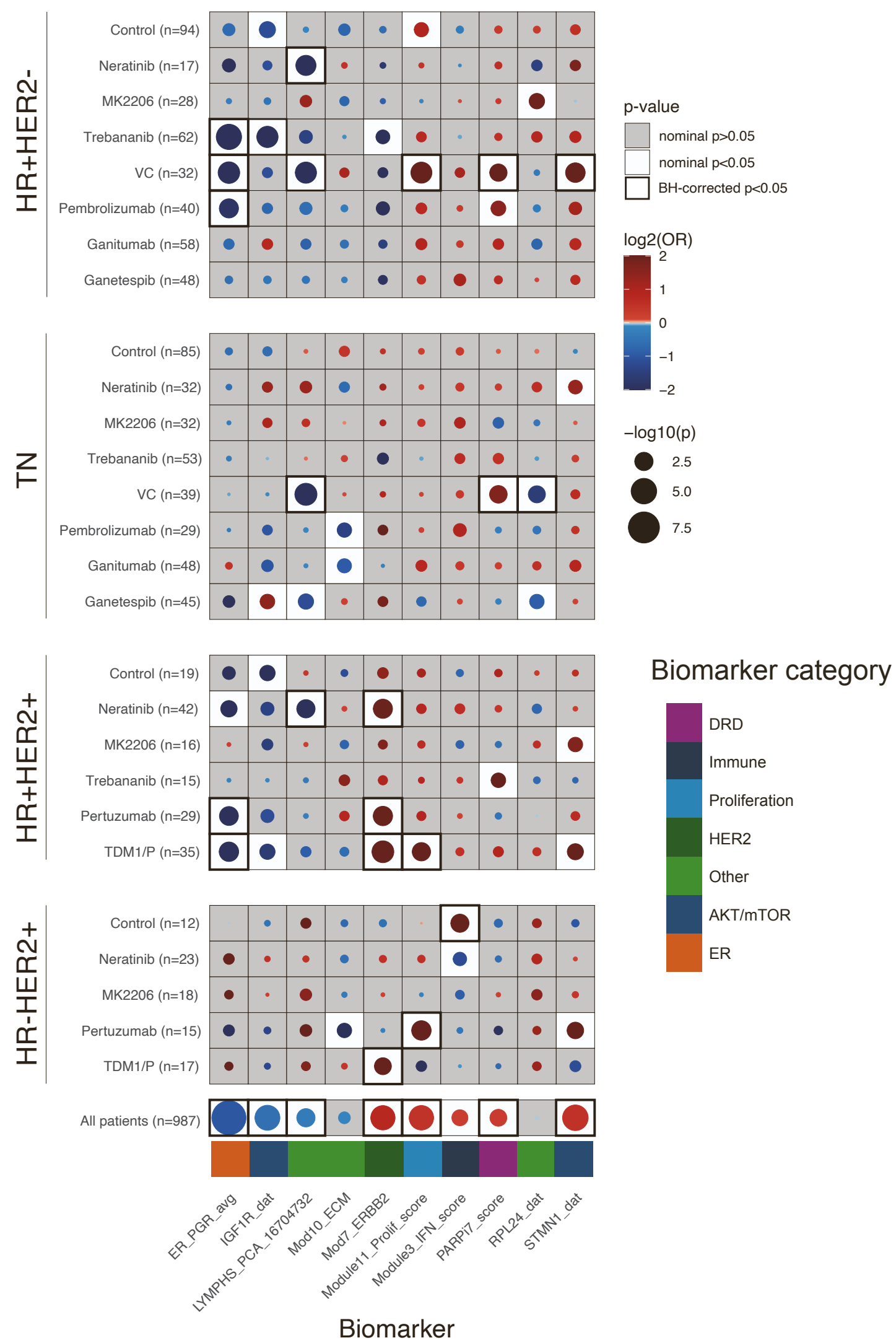

Figure S3. Results of pathologic complete response (pCR) association analysis in the I-SPY2 clinical trial are shown for each organoid-evaluable biomarker in each treatment arm by receptor subtype, based on gene expression and clinical data from the trial (Wolf et al., 2022) (right panel). Each row is a treatment arm. Dot color (red/blue) and intensity reflect the direction and strength of association with pCR, while dot size corresponds to the significance of the association. White background boxes indicate a nominal p-value < 0.05, and black border indicates False Discovery Rate (FDR) p-value <0.05 (Benjamini-Hochberg correction, BH). HR = hormone receptor. OR = odds ratio. p = p-value. TDM1/P = ado-trastuzumab emtansine plus pertuzumab (T-DM1/P). TN = triple negative. VC = veliparib-carboplatin. Related to Figure 2.

Figure S4

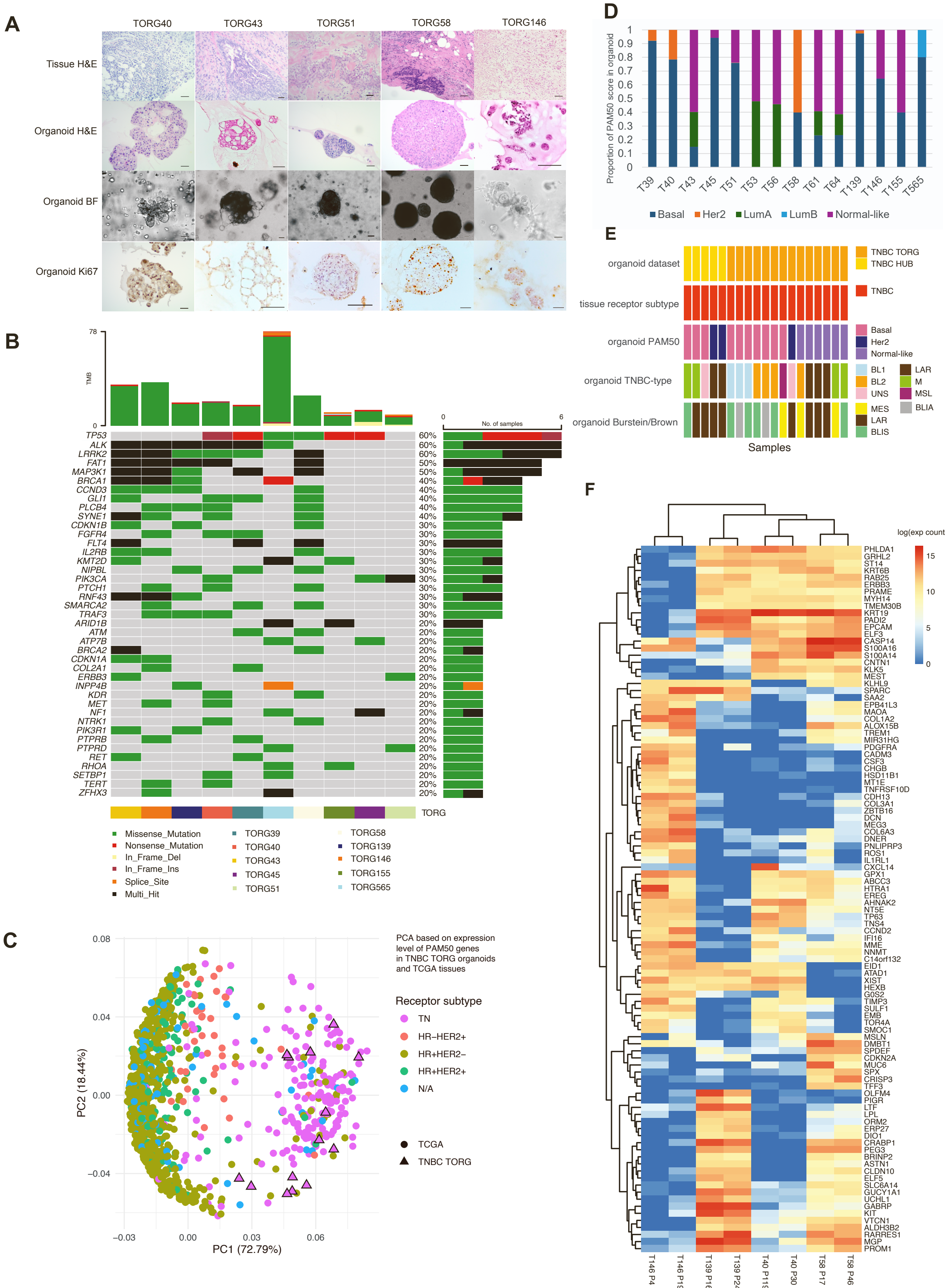

A
TORG40
TORG43
TORG51
TORG58
TORG146
Tissue H&E
Organoid H&E
Organoid BF
Organoid Ki67
B
TMB
78
0
No. of samples
TP53
ALK
LRRK2
FAT1
MAP3K1
BRCA1
CCND3
GLI1
PLCB4
SYNE1
CDKN1B
FGFR4
FLT4
IL2RB
KMT2D
NIPBL
PIK3CA
PTCH1
RNF43
SMARCA2
TRAF3
ARID1B
ATM
ATP7B
BRCA2
CDKN1A
COL2A1
ERBB3
INPP4B
KDR
MET
NF1
NTRK1
PIK3R1
PTPRB
PTPRD
RET
RHOA
SETBP1
TERT
ZFHX3
TORG
Missense_Mutation
Nonsense_Mutation
In_Frame_Del
In_Frame_Ins
Splice_Site
Multi_Hit
TORG39
TORG40
TORG43
TORG45
TORG51
TORG58
TORG139
TORG146
TORG155
TORG565
C
PCA based on expression level of PAM50 genes in TNBC TORG organoids and TCGA tissues
Receptor subtype
TN
HR−HER2+
HR+HER2−
HR+HER2+
N/A
TCGA
TNBC TORG
PC1 (72.79%)
PC2 (18.44%)
D
Proportion of PAM50 score in organoid
T39
T40
T43
T45
T51
T53
T56
T58
T61
T64
T139
T146
T155
T565
Basal
Her2
LumA
LumB
Normal-like
E
organoid dataset
tissue receptor subtype
organoid PAM50
organoid TNBC-type
organoid Burstein/Brown
Samples
TNBC TORG
TNBC HUB
TNBC
Basal
Her2
Normal-like
BL1
BL2
UNS
LAR
M
MSL
MES
LAR
BLIS
BLIA
F
log(exp count)
T146 P4
T146 P19
T139 P16
T139 P24
T40 P119
T40 P30
T58 P17
T58 P46

Figure S4. Validation of sensitivity/resistance biomarkers and cancer gene sets in the TNBC TORG organoid bank. BL1/BL2 = basal-like 1/2. BLIA = basal-like immune-activated. BLIS = basal-like immunosuppressed. Exp count = expected count (RSEM). HR = hormone receptor. LAR = luminal androgen receptor. LumA = luminal A. LumB = luminal B. M/MES = mesenchymal. MSL = Mesenchymal Stem-Like. N/A = not available. 'P' = passage number. TMB = tumor mutational burden. TN = triple negative. TNBC = triple negative breast cancer. UNS = a subtype could not be selected by the algorithm. Related to Figure 3.

A. Representative organoid cultures show diverse morphologies and phenotypes in culture, including botryoid, solid, cystic, and metaplastic/spindle cell phenotypes. Histology of the tumor tissue and embedded derived organoids (stained by Hematoxylin and Eosin; H&E), a brightfield (BF) image of the organoids in culture, and results of immunohistochemical staining for Ki67 are shown. Ki67 expression was evident in all cases that underwent additional immunohistochemical staining (n=7) with a range of expression levels, consistent with clinical variation in high-grade features. Scale bar = 100 μm.

B. Summary plot showing single-nucleotide variants (SNVs) and insertions/deletions in a representative subset of the tumor organoids (n=10), revealing the presence of mutations in breast cancer-associated genes across the biobank. Group B organoids (Figure 3D) generally did not have as many copy number alterations, but did exhibit mutations in cancer-associated genes, including TP53 and DIS3, and expressed Hallmark gene signatures, including higher epithelial mesenchymal transition and estrogen/androgen response signatures compared with organoids in group A (BH-corrected p-value <0.05) (Supplemental Table S5).

C. Principal component analysis (PCA) showing that organoids (triangles) cluster with The Cancer Genome Atlas (TCGA) tissue samples (circles) based on expression levels of PAM50 genes. Colors indicate breast cancer receptor subtypes. Axes include the percentage of variance explained by each principal component (PC).

D. Distribution of PAM50 score components for each organoid in the TNBC TORG biobank, showing heterogeneity in each case, with the highest score resulting in the final subtype call.

E. Hubrecht Organoid Technology (HUB) TNBC and TNBC TORG organoids reflect diverse breast cancer subtypes, identified by both general (PAM50) and TNBC-specific (TNBC-type and Burstein/Brown subtypes) classifications, compared to the receptor subtype of the original tumor specimen. The most frequent TNBC-type classifications were basal-like subtypes BL1 and BL2 (37% combined) and Luminal Androgen Receptor subtype (LAR; 26%). Using Burstein/Brown subtypes, basal-like immunosuppressed (BLIS) was the most frequent subtype among the TNBC organoids (32%), with fewer basal-like immune-activated (BLIA) cases (11%), as expected for organoids.

F. Heatmap comparing the expression of the top 100 differentially expressed genes in earlier and later passage cultures from four organoid models. Hierarchical clustering demonstrated that specimens from the same organoid model clustered more closely with one another than with other models across passages, supporting the relative preservation of model-specific transcriptional features over time in culture.

Figure S5

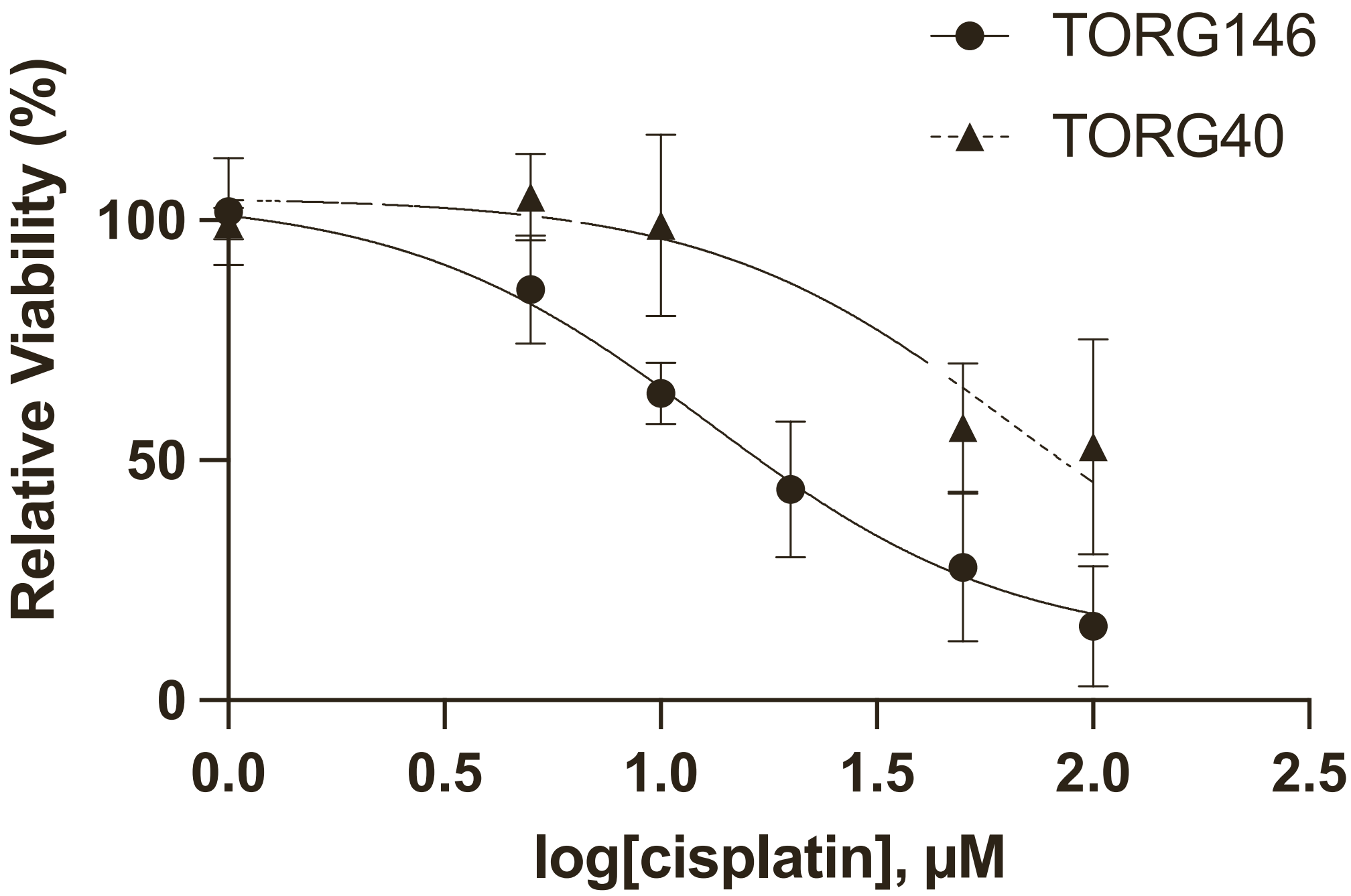

Figure S5. In vitro dose-response curve in TORG40 demonstrates resistance to cisplatin and dose-response curve in TORG146 demonstrates sensitivity to cisplatin. Relative cell viability (%) was determined at T=72 hours, and T=120 hours, respectively, using a luminescence-based cell viability assay (CellTiter-Glo). Data are expressed as the mean ± standard error of the mean (SEM) of three biological replicates. Related to Figure 5.

Table S1. Summary of clinical characteristics of HUB organoids included in the gene expression analysis (n=26). HR = hormone receptor. Related to Figure 1.

		HR+HER2- (n=17)	Triple-Negative (n=5)	HR-HER2+ (n=2)	HR+HER2+ (n=2)
**Grade**	1	0	1 (20%)	0	0
	2	11 (65%)	1 (20%)	0	2 (100%)
	3	6 (35%)	3 (60%)	2 (100%)	0
**Histological type**	Ductal carcinoma	14 (82%)	3 (60%)	2 (100%)	2 (100%)
	Lobular carcinoma	3 (18%)	0	0	0
	Other	0	2 (40%)	0	0
**Neoadjuvant therapy**	None	11 (65%)	4 (80%)	2 (100%)	1 (50%)
	Endocrine therapy	1 (6%)	0	0	0
	Chemotherapy	1 (6%)	0	0	1 (50%)
	Missing	4 (24%)	1 (20%)	0	0

Table S2. Details on multivariable logistic regression methods. Related to Figure 2.

A. Summary of significant multivariable logistic regression models to predict pathologic complete response (pCR) before refinement of the models. Using the I-SPY2-990 patient outcomes data (pCR) and the I-SPY2-990 patient tumor tissue expression level of these 9 organoid-evaluable biomarkers, we performed a multivariable logistic regression analysis across all receptor subgroup-treatment arm combinations. We used the step() function in R to perform forward selection and selected the resulting four models with three or more predictors. HR = hormone receptor. pCR = pathologic complete response. TN = triple negative.

Receptor subtype	Treatment arm	Model as identified by step() before refinement
HR+HER2-	Ganitumab (anti-IGF-1R)	pCR~Module11_Prolif_score + IGF1R_dat + LYMPHS_PCA_16704732 + Module3_IFN_score + STMN1_dat
HR+HER2+	Pertuzumab (anti-HER2)	pCR ~ Mod7_ERBB2 + PARPi7_score + ER_PGR_avg
HR+HER2+	T-DM1/Pertuzumab (anti-HER2)	pCR ~ Mod7_ERBB2 + Module11_Prolif_score + ER_PGR_avg + STMN1_dat
HR-HER2- (TN)	Veliparib/carboplatin (PARP inhibitor + platinum chemotherapy)	pCR ~ LYMPHS_PCA_16704732 + PARPi7_score + RPL24_dat

B. Final multivariable logistic regression models that predict treatment response in a specific receptor subtype after refinement. We manually reviewed the four models shown in A using summary(), and in case of any non-significant terms, we subsequently used drop1(model, test="Chisq") to remove variables that did not significantly contribute to the model (significant when $p<0.05$). Of these revised models, only three had more than one significant predictor. HR = hormone receptor. OR = odds ratio. P = predicted probability of pathologic complete response. pCR = pathologic complete response. TN = triple negative.

Receptor subtype	Treatment arm	OR predictor 1 (95% CI), p	OR predictor 2 (95% CI), p	OR predictor 3 (95% CI), p	Final model after refinement using drop1() including coefficients
HR+ HER2-	Ganitumab (anti-IGF-1R)	IGF1R_dat: 3.16 (1.03-9.75), p-value: 0.040	LYMPHS_PCA _16704732: 0.232 (0.0456-1.18), p-value: 0.072	n/a	log(P / (1 - P)) = -2.50 + 1.15* IGF1R_dat -1.46* LYMPHS_PCA_16704732
HR+ HER2+	Pertuzumab (anti-HER2)	Mod7_ERBB2: 3.90 (1.35-11.2), p-value: 0.00825	n/a	n/a	log(P / (1 - P)) = -1.84 + 1.36* Mod7_ERBB2
HR+ HER2+	T-DM1/ Pertuzumab (anti-HER2)	Mod7_ERBB2: 5.81 (1.07- 31.4), p-value: 0.036	Module11_ Prolif_score: 21.9 (1.32-364), p-value: 0.0252	ER_PGR_avg: 0.0421 (0.00147-1.21), p-value: 0.0542	log(P / (1 - P)) = 1.24 + 1.76* Mod7_ERBB2 + 3.09* Module11_Prolif_score - 3.17* ER_PGR_avg
HR- HER2- (TN)	Veliparib/ carboplatin (PARP inhibitor + platinum chemo-therapy)	LYMPHS_PCA_ 16704732: 0.0660 (0.00950- 0.458), p-value: 0.00445	PARPi7_ score: 4.82 (1.32-17.6), p-value: 0.0137	n/a	log(P / (1 - P)) = -1.95 - 2.72*LYMPHS_PCA_ 16704732 + 1.57*PARPi7_score

C. Receiver operating characteristic (ROC) curves showing the performance of the multivariable logistic regression model for predicting pCR with area under the curve (AUC) values for final models that predict treatment response in a specific receptor subtype after refinement using drop1() to exclude redundant predictors. AUC values closer to 1.0 indicate better performance of the model.

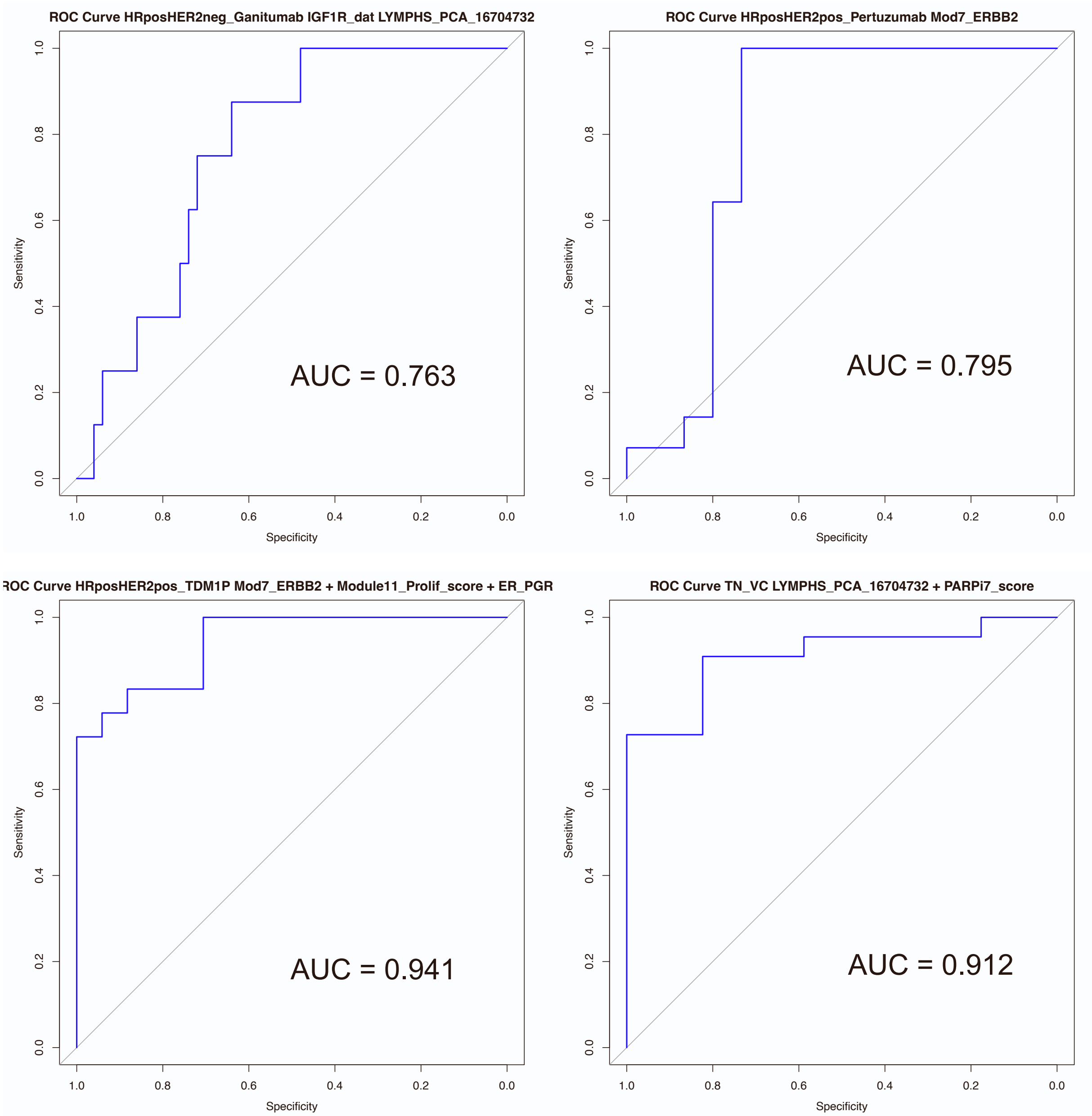

Supplement: Supplementary file 1 [file mmc1.pdf]
