# Supplementary material for: Biomarker-guided responses in patient-derived organoids predict effective therapies in breast cancer
Source: Cell Rep Med. 2026 Aug 6;7(8):102973. doi: 10.1016/j.xcrm.2026.102973 (PMC13522806; doi:10.1016/j.xcrm.2026.102973)

Cell Reports
Medicine

Article

Biomarker-guided responses in patient-derived organoids predict effective therapies in breast cancer

Graphical abstract

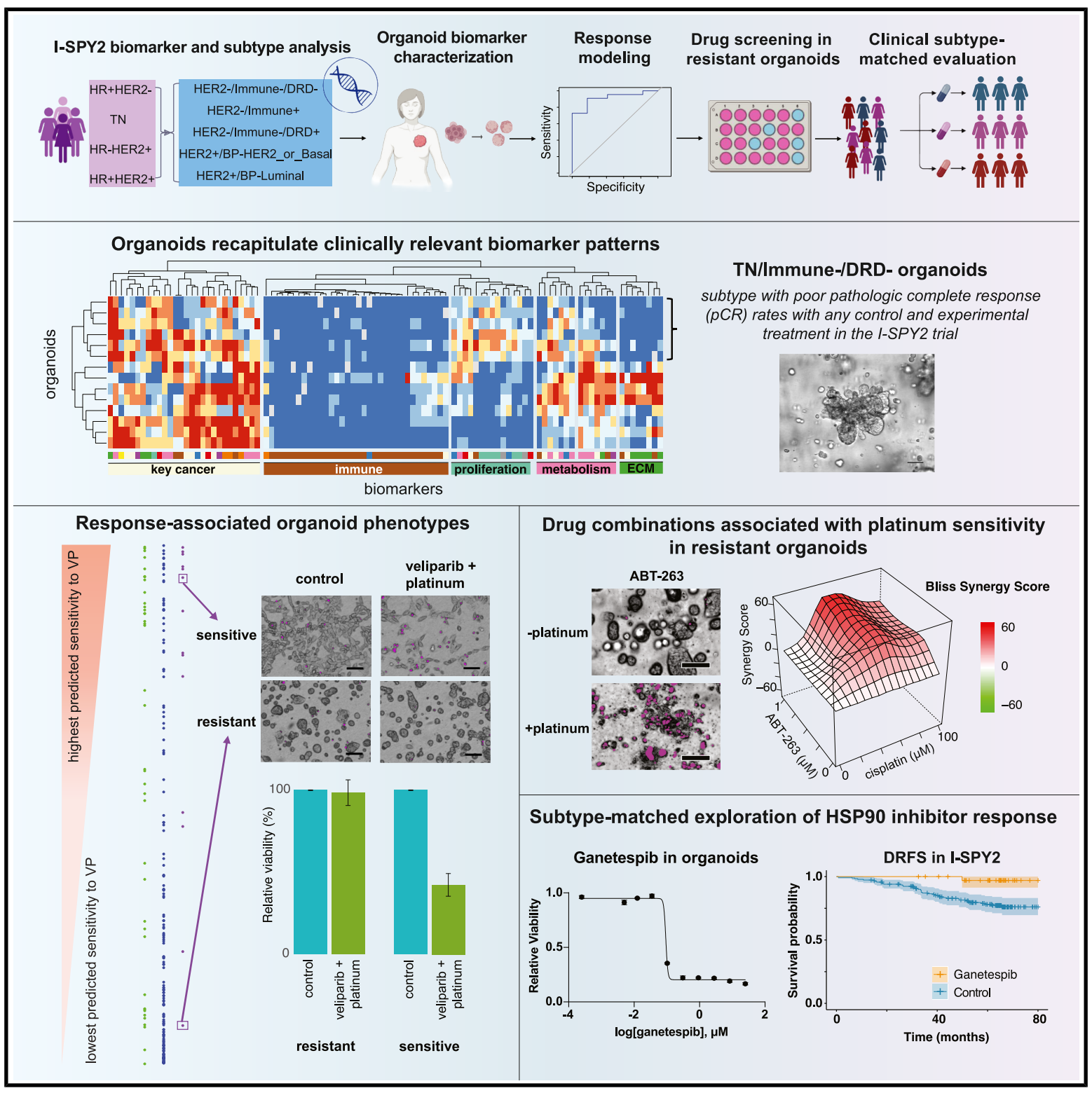

Authors

Tam Binh V. Bui, Denise M. Wolf, Michael C. Bruck, ..., Laura J. Esserman, Laura J. van 't Veer, Jennifer M. Rosenbluth

Correspondence

laura.vantveer@ucsf.edu (L.J.v.'t.V.), jennifer.rosenbluth@ucsf.edu (J.M.R.)

In brief

Bui et al. demonstrate that response-predictive models derived from patient biomarkers and clinical outcomes accurately predict *in vitro* responses in organoids using only biomarkers measurable in organoids as input and inform subtype-specific drug screening efforts. This highlights the potential of integrating organoids into personalized medicine strategies through gene-expression-based characterization.

Highlights

- Clinical biomarkers guide analysis of 40 patient-derived breast cancer organoids
- I-SPY2 patient data enable the development of treatment-response-predictive models
- Organoid drug responses validate predictions from clinical response models
- Subtype-specific drug screening identifies candidate breast cancer therapies

Bui et al., 2026, Cell Reports Medicine 7, 102973
August 18, 2026 © 2026 The Author(s). Published by Elsevier Inc.
https://doi.org/10.1016/j.xcrm.2026.102973

CellPress

Cell Reports Medicine

CellPress
OPEN ACCESS

Article

Biomarker-guided responses in patient-derived organoids predict effective therapies in breast cancer

Tam Binh V. Bui,[1,2,3,4] Denise M. Wolf,[1] Michael C. Bruck,[2] Kaitlin Moore,[5] Jessica Lien,[2] Sarah D.W. Choi,[6] Shruti Warhadpande,[2] Amirabbas Parizadeh,[2] Deborah Dillon,[7] Beth Overmoyer,[5] Filipa Lynce,[5] Lamorna Brown-Swigart,[1] Gillian L. Hirst,[6] Christina Yau,[6] I-SPY2 Investigators, Isaac J. Nijman,[8] Boudewijn M.T. Burgering,[3,4] Isaac S. Harris,[9] Laura J. Esserman,[6] Laura J. van 't Veer,[1,*] and Jennifer M. Rosenbluth[2,5,10,11,*]

[1]Department of Laboratory Medicine, Helen Diller Comprehensive Cancer Center, University of California, San Francisco, San Francisco, CA 94143, USA
[2]Department of Medicine, Division of Hematology & Oncology, Helen Diller Comprehensive Cancer Center, University of California, San Francisco, San Francisco, CA 94143, USA
[3]Molecular Cancer Research, Center for Molecular Medicine, University Medical Center Utrecht, 3584 CX Utrecht, the Netherlands
[4]Oncode Institute, 3521 AL Utrecht, the Netherlands
[5]Division of Medical Oncology, Dana-Farber Cancer Institute, Harvard Medical School, Boston, MA 02215, USA
[6]Department of Surgery, Helen Diller Comprehensive Cancer Center, University of California, San Francisco, San Francisco, CA 94143, USA
[7]Department of Pathology, Brigham & Women's Hospital, Boston, MA 02215, USA
[8]USEQ, Center for Molecular Medicine, University Medical Center Utrecht, 3584 CX Utrecht, the Netherlands
[9]Department of Biomedical Genetics, Wilmot Cancer Institute, Department of Pharmacology and Physiology, University of Rochester Medical Center, Rochester, NY 14642, USA
[10]Chan Zuckerberg Biohub – San Francisco, San Francisco, CA 94158, USA
[11]Lead contact
*Correspondence: laura.vantveer@ucsf.edu (L.J.v.'t.V.), jennifer.rosenbluth@ucsf.edu (J.M.R.)
https://doi.org/10.1016/j.xcrm.2026.102973

SUMMARY

Poor therapeutic response in subsets of breast cancer (BC) patients poses an ongoing challenge. Here, we present a biomarker-guided characterization of 40 patient-derived BC organoids, with the aim of modeling resistant disease with greater fidelity and developing an *in vitro* system grounded in clinical data for testing alternative treatment strategies. We utilize patient data from the I-SPY2 clinical trial (NCT01042379) to develop predictive models of response to a range of therapies, using only organoid-detectable biomarkers as input, and validate a model predicting response to veliparib-platinum chemotherapy (VP) in triple-negative BC (TNBC) organoids. A drug screen in VP-resistant TNBC organoids reveals combination treatments that overcome resistance to cisplatin, including pro-apoptotic therapies. Another class of hits, HSP90 inhibitors, links organoid drug sensitivity to improved recurrence-free survival in a biomarker-defined patient subset. These findings establish organoid-based functional modeling as a bridge between clinical biomarkers and precision treatment strategies in breast cancer.

INTRODUCTION

An important challenge in cancer research is developing robust and representative research models that can accurately recapitulate the complexity of the disease.[1] While traditional cell lines have provided valuable insights, they often fail to capture the full spectrum of cancer subtypes and fail to maintain the wide range of genetic, phenotypic, and molecular heterogeneity seen in the original tumors,[2] in part because the rate of successful establishment of cell lines from primary tumor specimens remains low. Patient-derived organoids, in contrast, can be established with higher efficiency and can recapitulate important histological and genetic features of the tumor tissue.[3,4] Another major area of recent advancement is the development of clinical biomarkers that can predict patient treatment response and support clinical decision-making.[5] It remains unknown, however, whether and to what extent clinical gene-expression-based biomarkers and predictive models derived from and predictive in patients can be applied to organoid models. While key studies have evaluated individual or small numbers of markers in cancer organoids,[6,7] it remains largely undetermined which gene-expression-based biomarkers can be modeled in organoids.

This is particularly relevant in breast cancer, where drug development and clinical trial progress in recent years have led to an increased number of treatment options available to patients, creating a need for better biomarkers and subtyping schemas to

© 2026 The Author(s). Published by Elsevier Inc.
This is an open access article under the CC BY license (http://creativecommons.org/licenses/by/4.0/).

identify and predict the populations that will benefit. The ongoing, multi-center phase 2 neoadjuvant chemo-/targeted-therapy I-SPY2 platform trial (NCT01042379) was designed to rapidly screen promising experimental agents and identify improved treatment regimens in women with early-stage, high-molecular-risk breast cancer, as well as to identify and propose potential biomarkers of response and resistance. Between 2010 and 2022, 24 therapies were tested in 2,117 patients. The primary endpoint of the trial is pathologic complete response (pCR), defined as the absence of invasive breast cancer in the breast and regional nodes at the time of surgery due to the complete eradication of the cancer cells by the preceding therapy.

The trial developed the response predictive subtype (RPS), a breast cancer classification schema designed to better segregate tumors based on anticipated responses to therapies in a modern treatment landscape, including immunotherapy, PARP-inhibition, platinum drugs, and dual-HER2 inhibition, by combining immune, DNA repair deficiency (DRD), and luminal-like biological phenotypes with hormone receptor (HR) and HER2 status.[5] Importantly, the efforts to improve breast cancer classification have identified subsets of patients who do not respond to neoadjuvant targeted and/or chemotherapy. Approximately 30% of the patients in the trial had HER2-negative tumors with low expression of immune genes and no DRD ($HER2^-$/$immune^-$/DRD^- subtype).[5] These patients had very low pCR rates (~10% on average) across all treatment and control arms. A deeper understanding of the molecular mechanisms driving breast cancer resistance and more approaches to overcome this treatment resistance are needed. Organoids hold potential for identifying personalized treatment strategies for patients with cancer, particularly if they can be shown to stratify patients using the same biomarkers that can do this in a clinical setting.

In this paper, we evaluated a biobank of early-stage invasive breast cancer organoids characterized using I-SPY2 predictive biomarkers as a tool to model and study mechanisms of therapy resistance. As a proof of concept, we developed organoid-tailored predictive models derived from I-SPY2 patient biomarker and clinical trial data and used these to predict treatment sensitivity and resistance in subtype-matched organoid cultures. We validated resistant organoid cultures that were then selected and used for compound screening to identify effective treatment options for biomarker-identified resistant tumors. While drug screening in patient-derived and PDX-derived breast cancer and other cancer organoids has previously been reported to be feasible and useful,[8–20] we report here a high-throughput, 386-compound combination drug screen across 10 doses in patient-derived breast cancer organoids that yielded promising multi- and single-agent therapies and identified a significant survival benefit in subtype-matched patient data.

RESULTS

Organoids recapitulate most breast tumor subtypes

We hypothesized that organoids retain the expression of clinically relevant gene expression biomarkers representing key biological pathways in cancer and that these biomarkers can be used to identify treatment-resistant organoids, which can be further used for drug testing to overcome resistance (Figure 1A). To this end, we evaluated bulk RNA-sequencing data from the published Hubrecht Organoid Technology (HUB) Living BC Organoid Biobank,[3] which contained 26 early-stage breast tumor organoids across all receptor subtypes of breast cancer (n = 17 HR^+HER2^-, n = 5 triple-negative [TN], n = 2 HR^+HER2^+, n = 2 HR^-HER2^+; hormone receptor [HR] meaning either estrogen receptor (ER) or Progesterone Receptor [PR] positive) (Table S1). To enable interpretation of the HUB organoid data in a larger (clinical) breast cancer context, we quantile-normalized the organoid gene expression data to bulk RNA sequencing data from TCGA breast tumor tissue samples, resulting in an integrated dataset for further analysis (quality controls for merge/integration in Figure S1).

Principal-component analysis based on the expression of PAM50 genes revealed that organoids largely cluster with TCGA samples of the same receptor subtype (Figure 1B), with one exception: HR^+HER2^-organoids are distributed more loosely across the TCGA samples, possibly due to the loss of ER signaling in organoid culture, as HUB organoids were cultured using a version of organoid medium not supplemented with estrogen. This is also reflected by the distribution of PAM50 scores in the HR^+HER2^- organoids (Figures 1C and 1D), with some distributions seeming to represent decreased luminal phenotype and other distributions potentially reflecting intratumoral heterogeneity. Generally, our subtype predictions matched well with the previously published calls for these dataset (>85% close match), with the possible exception of luminal-A-classified organoids.[3] Loss of ER signaling with extended culturing is an ongoing point of attention in the field, with multiple recent efforts to restore ER signaling in organoid culture.[21–25] Overall, a strong separation of basal and non-basal organoids was observed by unsupervised clustering (Figure S2).

Patient-derived organoids retain expression of most cancer-related gene signatures, consistent with clinical/pathologic tumor features

We then analyzed the expression of the 50 Molecular Signatures Database (MSigDB) Hallmark gene sets[26] by GSVA and a selection of 50 gene signatures representing key cancer signaling pathways from the I-SPY2 biomarker compendium, many of which correlate with treatment sensitivity and resistance in patients,[5] in HUB organoids compared with receptor-subtype-matched TCGA tissues. Organoids in the biobank showed variable expression of ~75% of the tested biomarkers, recapitulating key biological features of tumors, including DNA damage response, Akt/mTOR signaling, and Myc/proliferation signatures (Figure 1E). Biomarkers were considered variably expressed (or "organoid-evaluable") if they were expressed at levels greater than the 10th percentile in at least 10% of the biobank, compared with receptor-subtype-matched TCGA tissues. While most immune signatures were negative in organoids, likely due to the loss of immune cells after organoid passaging (e.g., B cells and Hallmark_IL2_STAT5_signaling), several other immune signatures were detectable in organoids, likely reflecting epithelial expression of those predictive markers (e.g., programmed cell death protein 1 [PD1] and interleukin-13 [IL-13]). The expression of clinically relevant

CellPress
OPEN ACCESS

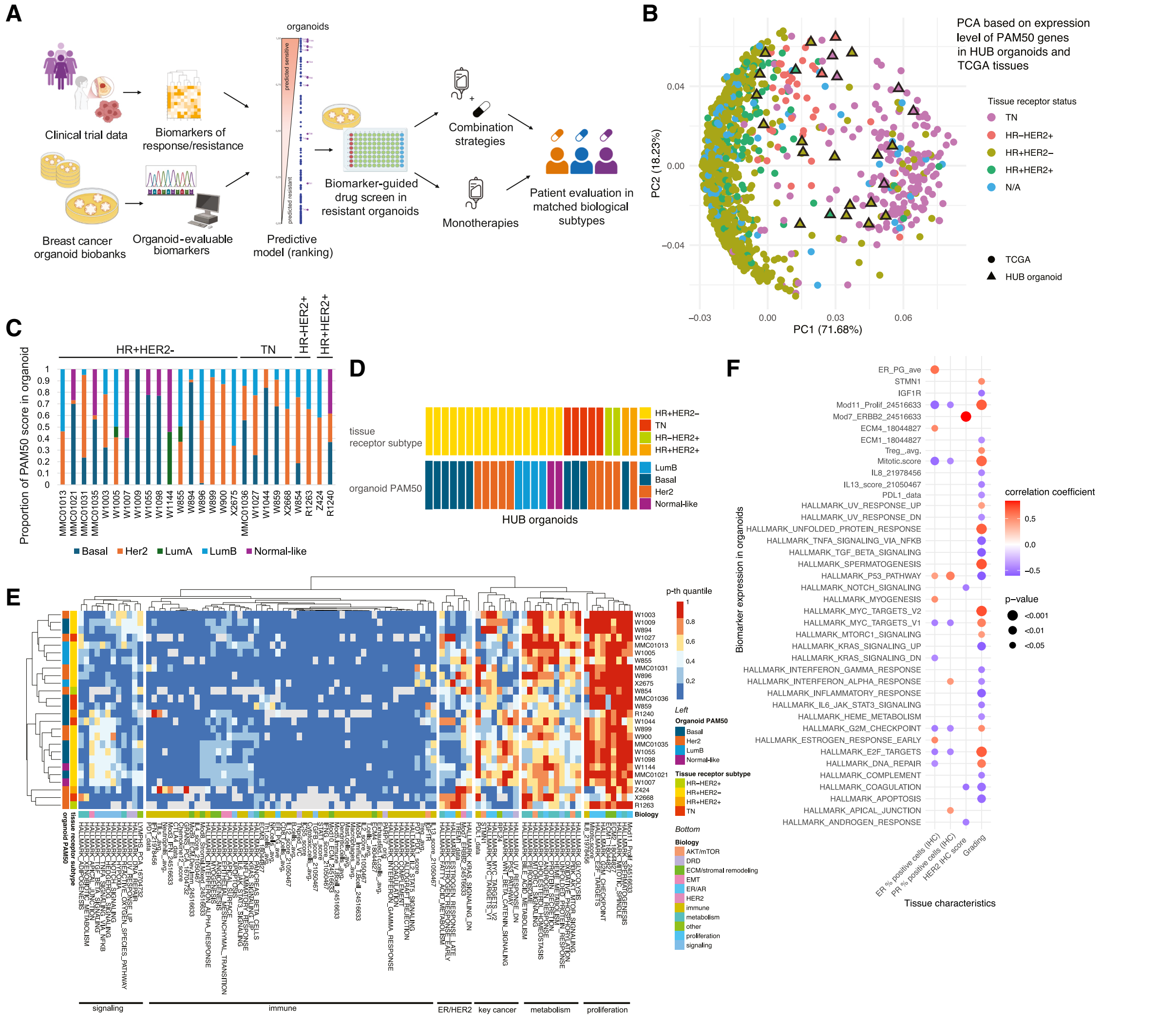

Figure 1. Breast cancer subtypes and clinical biomarkers can be modeled in breast cancer organoids

BLIS, basal-like immunosuppressed; DRD, DNA repair deficiency; ECM, extracellular matrix; EMT, epithelial-mesenchymal transition; ER/AR, estrogen receptor/androgen receptor; HR, hormone receptor; HUB, Hubrecht Organoid Technology; IHC, immunohistochemistry; LAR, luminal androgen receptor; LumA, luminal A; LumB, luminal B; M, mesenchymal; N/A, not available; PR, progesterone receptor; TCGA, The Cancer Genome Atlas; TN, triple-negative.

(A) Graphical overview of the approach to model clinical trial data in organoids and identify biomarker-guided combination therapies.

(B) Principal-component analysis (PCA) showing that organoids (triangles) cluster with TCGA tissue samples (circles) based on expression levels of PAM50 genes. Colors indicate breast cancer subtypes. See also Figure S1.

(C) Distribution of PAM50 scores components for each organoid in the HUB biobank, showing heterogeneity in each case, with the highest score resulting in the final subtype call.

(D) Subtyping of HUB organoids based on gene expression is shown for PAM50 compared to the receptor subtype of the original tumor specimen.

(E) Heatmap showing the relative expression (p-th quantile) of gene expression biomarkers from the Molecular Signatures Database (MSigDB) HALLMARK set and 50 key biomarkers from the I-SPY2 trial compendium in HUB breast cancer organoids. See also Figure S2.

(F) Dot plot showing significant results of Pearson correlation analysis between expression scores of organoid-evaluable biomarkers in organoids (*y* axis) and clinical features of the corresponding tumors (*x* axis). Negative correlations indicate inverse associations between organoid biomarker scores and clinicopathologic tumor features. Dot color (red/blue) and intensity reflect the direction and strength of the correlation, while dot size corresponds to the statistical significance. See also Table S1.

biomarkers in organoids correlated with the corresponding clinicopathologic features of the source tumors, including HR status, HER2, and tumor grade (Figure 1F). Proliferation scores were negatively correlated with ER staining and PR staining ($p < 0.05$), but strongly positively correlated with tumor grade, as expected. In contrast, expression of immune- and apoptosis-related signatures was negatively correlated with tumor grade ($p < 0.05$). Stromal tumor-infiltrating lymphocytes (TILs) have previously been associated with higher rates of pathological complete response to neoadjuvant chemotherapy independent of other clinicopathological prognostic factors or chemotherapy regimens.[27,28]

Generation of a predictive model for organoid treatment response using biomarkers and patient response data from the I-SPY2 clinical trial

We next hypothesized that these clinically relevant tissue biomarkers could be used to match organoids to drug sensitivity and resistance profiles. To this end, we first sought to derive a predictive model using patient data that could be tested in organoids.

To build this model, we assessed the published I-SPY2-990 dataset of qualifying I-SPY2 biomarkers associated with pathologic complete response (pCR) in 987 patient samples across the first 10 treatment arms of the I-SPY2 clinical trial (Figures 2A and 2B).[5] We first filtered the MSigDB Hallmark/I-SPY signature set of 100 signatures to select only the 23 signatures that were previously validated in CLIA-certified labs and associated with clinical outcome (termed "qualifying" biomarkers in I-SPY2).[5] We then further filtered this list to include only biomarkers that were detectable in organoid cultures, based on our assessment of the HUB organoid database, and excluded proprietary biomarkers (e.g., BluePrint/MammaPrint[29,30]), resulting in a list of 9 out of the 23 published qualifying gene expression markers that could be assessed in organoids (Figure 2C).

For each receptor subtype and treatment combination in I-SPY2, a number of organoid-evaluable biomarkers were significantly associated with pCR (response) or non-pCR (resistance) (Figure 2D, triple-negative breast cancer [TNBC]; Figure S3, all receptor subtypes). We tested multivariable logistic regression models using the nine selected organoid-evaluable qualifying biomarkers as input to predict pCR in each treatment-receptor subtype combination in the I-SPY2 dataset and identified three models with more than one statistically significant organoid-detectable predictor of response. PARPi7_score and LYMPHS_PCA_16704732 were statistically significant predictors of response to veliparib-carboplatin (VC) in patients with TNBC, insulin-like growth factor 1 receptor (IGF1R), and LYMPHS_PCA_16704732 were statistically significant predictors of response to ganitumab in patients with $HR^{+}HER2^{-}$ tumors, and Mod7_ERBB2, Module11_Prolif_score, and ER_PGR_avg were significant in a model predicting response to TDM1/P in patients with $HR^{+}HER2^{+}$ tumors (Tables S2A–S2C).

Here, we focused on VC, an investigational combination that includes the poly (ADP-ribose) polymerase (PARP)-1 and -2 inhibitor veliparib and carboplatin, a platinum chemotherapy that acts as an alkylating agent, causing cross-linking between and within DNA strands.[31] Both drugs have single-agent anti-tumor activity in patients with BRCA-mutation-associated breast cancer and TNBC, and it has been hypothesized that they could synergize when given together,[32–35] though a more recent report[36] that evaluated the contributions of each of these drugs to improved long-term outcomes in TNBC demonstrated that carboplatin alone and not veliparib contributed to improved outcomes, highlighting the importance of studying resistance to platinum chemotherapies.

In the TNBC subset treated with VC in I-SPY2, three biomarkers (PARPi7, LYMPHS_PCA, and RPL24) within the set of nine organoid-evaluable biomarkers were individually significantly associated with pCR (Figure 2E). The PARPi7 gene set consists mostly of DNA-repair-related genes and was positively associated with treatment response.[37] RPL24 is a single-gene biomarker for the RPL24 gene that encodes the large ribosomal subunit protein eL24.[38] RPL24 expression is commonly elevated in breast cancer, and depletion or structural alteration of RPL24 has been shown to significantly impair human breast cancer cell viability.[39,40] LYMPHS_PCA is a gene set also mostly representing ribosomal proteins and cytoplasmic translations[41] and both were negatively associated with treatment response.[5] In the multivariable logistic regression model, only PARPi7 and LYMPHS_PCA remained statistically significant (Tables S2A and S2B, bottom rows). The area under the receiver operating characteristic (ROC) curve for the model using PARPi7 and LYMPHS_PCA to predict the probability of pCR to veliparib-carboplatin in patients with TNBC was 0.912 (Figure 2F). This model, trained exclusively on clinical I-SPY2 patient data, was chosen for independent organoid-based validation to test whether it can predict treatment response to veliparib-platinum chemotherapy (VP) in triple-negative organoid cultures based on organoid gene expression.

TNBC organoids recapitulate subtypes of basal breast cancers and express key breast cancer gene signatures

To further evaluate TNBC subtypes and test whether treatment responses predicted by clinical-data-trained models could be recapitulated in organoids, we established a second, larger TNBC breast tumor organoid biobank (termed TNBC TORG) to expand the number of TNBCs in our dataset (Figure 3A). This TNBC TORG biobank consisted of 14 organoid cultures derived from primary triple-negative breast tumors, representing diverse subtypes including inflammatory and metaplastic cancers (Table 1; Table S3; Figure S4A).

Next-generation targeted oncology sequencing in a subset of matched tumor organoids and primary tumor tissues showed high concordance in variant allele frequencies (VAFs) between organoids and matched primary tumors, with many cancer-associated mutations identified in the primary tumors also detected in the corresponding organoid cultures at similar or enriched frequencies (Figure 3B), consistent with preservation and enrichment of epithelial tumor populations during organoid propagation. Among genomically validated organoid lines, copy-number variation (CNV) burden varied (Figure 3C). CNV analysis demonstrated that most TNBC TORGs (7/10 tested cases; 70%) exhibited substantial copy-number alterations, including recurrent gains commonly associated with breast cancer, such as 1q and 8q amplifications[42] (see also Figure S4B). Validated organoids with lower CNV burden nonetheless had clear mutation concordance with the primary tumor (Figure 3B), consistent with prior observations that genomic instability varies across TNBC subtypes.[43,44]

TNBC TORG gene expression data were normalized and integrated with triple-negative TCGA breast cancer tissues in the same way as the HUB biobank (Figure S4C), with characterization of each organoid using TNBC subtyping schemas (Figures S1; Figures S4D and S4E). TNBC organoids exhibited substantial transcriptional heterogeneity (Figure 3D; Table S4), including differences in genomic instability (Figure 3C), growth rates (Table S3), and pathway activity (Figure 3E). HER2 biomarker scores were highly variable, which suggests that some of these TNBC organoids might exhibit HER2-low phenotypes despite not meeting the

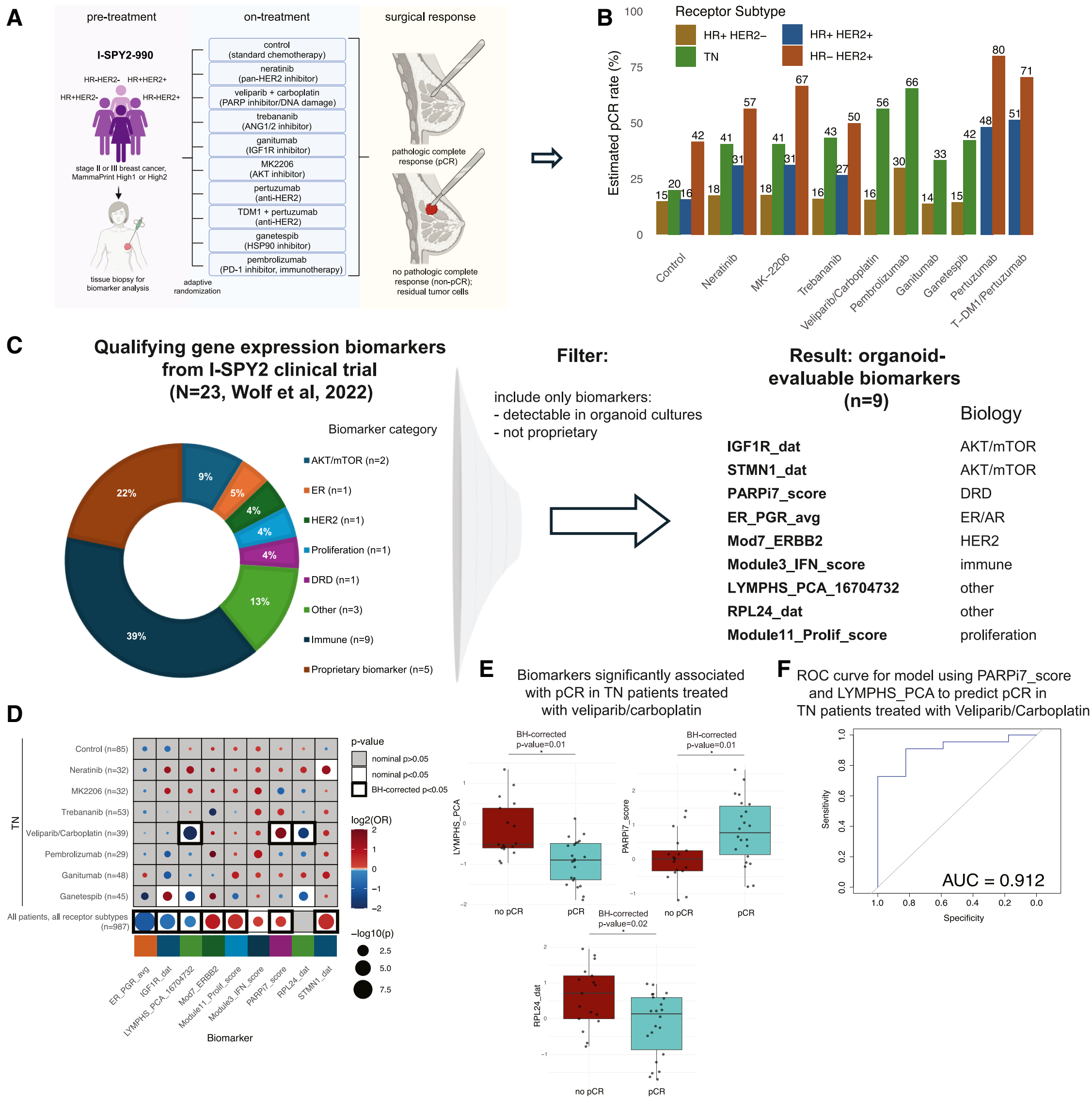

Figure 2. Systematic selection of I-SPY2 gene-expression-based biomarkers to build a predictive model of treatment response applicable to patient-derived organoids

ANG1/2, angiopoietin 1/2; AR, androgen receptor; BH, Benjamini-Hochberg; DRD, DNA repair deficiency; ER, estrogen receptor; HR, hormone receptor; HSP90, heat shock protein 90; HUB, Hubrecht Organoid Technology; IGF1R, insulin-like growth factor 1 receptor; OR, odds ratio; PARP, poly (ADP-ribose) polymerase; PD-1, programmed cell death protein 1; pCR, pathologic complete response; TDM1, ado-trastuzumab emtansine; TN, triple-negative.

(A) Schematic of the I-SPY2 clinical trial connecting biomarker discovery to pCR.

(B) Bar graph showing predicted pCR rates to each of the first 10 treatment arms of the ISPY2 clinical trial in each breast cancer receptor subtype. Numbers shown are the % pCR. Adapted from Wolf et al., 2022.[5]

(C) Twenty-three qualifying biomarkers from I-SPY2 (summarized by biomarker category in the left panel) were filtered to select only biomarkers evaluable in organoids (n = 9/23), based on observed expression levels in the HUB dataset and exclusion of proprietary biomarkers.

(D) Results of pCR association analysis in the I-SPY2 clinical trial for each organoid-evaluable biomarker in each treatment arm in triple-negative breast cancer (TNBC), based on gene expression and clinical data from the trial. Dot color (red/blue) and intensity reflect the direction and strength of association with pCR, while dot size corresponds to the significance of the association. White background boxes indicate a nominal p value <0.05, and black border indicates BH-corrected p value <0.05. See also Figure S3.

(E) Distribution of the three biomarkers that were significantly differentially expressed in TN tumors in cases with or without subsequent pCR to neoadjuvant treatment with veliparib-carboplatin (Wilcoxon rank-sum test with BH correction).

(F) Receiver operating characteristic (ROC) curve showing the performance of the final, refined, multivariable logistic regression model for predicting pCR to veliparib-carboplatin (VC) in patients with TNBC. The model incorporating both PARPi7 score and LYMPHS_PCA score demonstrates an area under the curve (AUC) of 0.912, suggesting good predictive performance. See also Table S2.

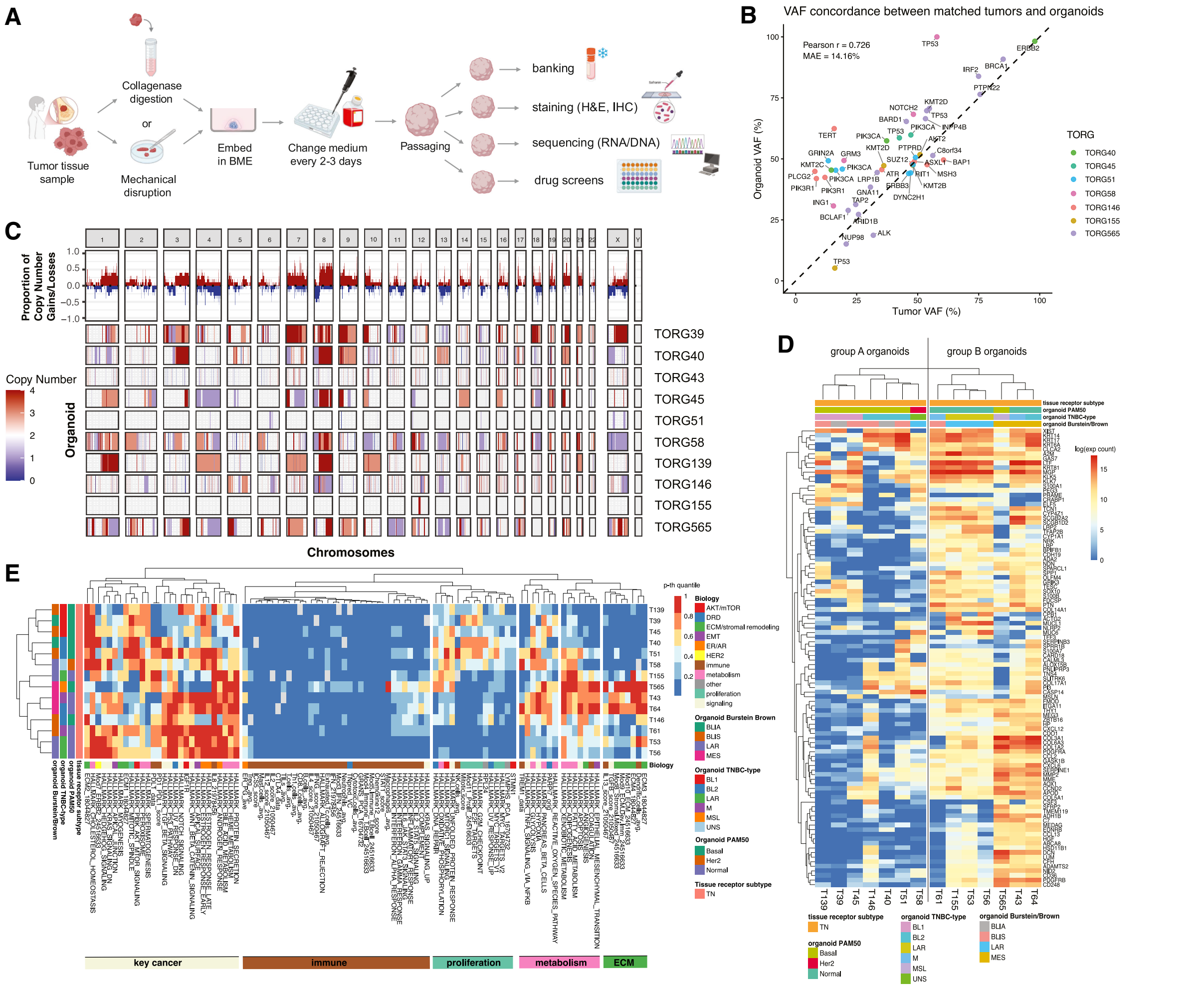

Figure 3. Generation and characterization of a TNBC organoid bank comprising multiple TNBC subtypes

See also Figure S4 and Tables S3–S7. AR, androgen receptor; BME, basement membrane extract; DRD, DNA repair deficiency; ECM, extracellular matrix; EMT, epithelial-mesenchymal transition; ER, estrogen receptor; Exp count, expected count (RSEM); H&E, hematoxylin & eosin; IHC, immunohistochemistry; MAE, mean absolute error.

(A) Tissue processing and organoid culturing pipeline for establishing organoids from surgical specimens.

(B) Variant allele frequency (VAF) concordance plot between matched tumors and organoids shows concordance between organoids and matched primary tumor tissue.

(C) Genomic copy-number variation (CNV) profiles were assessed for the indicated breast cancer organoids ($n = 10$). Gains (amplifications) and losses (deletions) are indicated by red and blue colors, respectively. Quantification of recurrent gains and losses across the dataset is shown in the last row.

(D) Unsupervised clustering of TNBC TORG organoids based on the top 100 most differentially expressed genes across the dataset identifies two subclusters, A and B. Observed differences in growth rates and morphologic features between group A and group B organoids were correlative and may reflect underlying biologic differences between organoid lines.

(E) Unsupervised clustering of TNBC TORG organoids based on the relative expression (p-th quantile) of gene expression biomarkers from the Molecular Signatures Database (MSigDB) HALLMARK set and key biomarkers from the I-SPY2 trial compendium identifies a subset of TN/immune^{-}/DRD^{-}-like organoids (T139, T39, T45, T51, T40, and T58).

clinical criteria for HER2 positivity.[45,46] There were no inflammatory breast-cancer-specific biomarkers (Table S5). Representative organoid-evaluable signatures were generally preserved across time and passaging (Table S6; Figure S4F), consistent with other previously described tumor-intrinsic signatures.[47]

Lastly, we identified a subset of six organoids (TORG39, TORG40, TORG45, TORG51, TORG58, and TORG139) that showed biomarker expression patterns highly similar to the TN/immune^{-}/DRD^{-} tumors in I-SPY2 (Figure 3E; Table S7), further supporting the use of these organoids as representative models of the TN/immune^{-}/DRD^{-} subtype, the subtype in I-SPY2 with the poorest treatment outcomes.[5]

Validation of a clinical predictive model for treatment response using TNBC organoids *in vitro*

Using gene expression biomarker data from our second, larger TNBC TORG biobank ($n = 14$), we tested the clinical-data-derived model developed here for predicting organoid treatment

Table 1. Summary of clinical and organoid culture characteristics of TNBC TORG included in the gene expression analysis (*n* = 14)

			Triple-negative (*n* = 14)
Clinical characteristics	grade	1	1 (7%)
	–	2	2 (14%)
	–	3	11 (79%)
	tumor histological type	ductal carcinoma	11 (79%)
	–	lobular carcinoma	0
	–	other	3 (21%)
	inflammatory breast cancer	yes	4 (29%)
	–	no	10 (71%)
	neoadjuvant therapy	none	4 (29%)
	–	chemotherapy	9 (64%)
	–	other	1 (7%)
Organoid characteristics	medium type	type 1	10 (71%)
	–	type 2	4 (29%)
	organoid morphology	solid	9 (64%)
	–	mixed	5 (36%)

response to VP. We ranked the 14 organoids from the highest model-predicted sensitivity score to the lowest model-predicted sensitivity score (Figure 4A). Prediction scores for the reference dataset (I-SPY2-990 TNBC in the VP arm), organoids used to identify organoid-evaluable biomarkers (HUB TNBC organoids), and testing datasets (TNBC TCGA tissues and TNBC TORG organoids) spanned the full range from predicted high VP sensitivity to high VP resistance. The TN/immune$^-$/DRD$^-$ organoids were among the organoids with the lowest predicted sensitivity to VP.

We evaluated the calculated sensitivity scores for I-SPY2 patients using our predictive model and correlated them with pCR (Figure 4B). We identified one tertile of patients with high predicted sensitivity scores (>0.85), all of whom achieved a pCR (*n* = 14/14 pCR, 100%): one tertile of patients with low predicted sensitivity scores (<0.3) who mostly did not achieve a pCR (*n* = 2/13 pCR, 15%) and an intermediate subgroup with predicted sensitivity scores between 0.3 and 0.85 who had mixed pCR outcomes (*n* = 6/12 pCR, 50%). Similarly, translating these clinical-data-based thresholds to the TNBC TORGs set, we identified a model-predicted sensitive group with a score >0.85 (*n* = 7), an intermediate group with a score between 0.3 and 0.85 (*n* = 5), and a model-predicted resistant group with a predicted sensitivity score <0.3 (*n* = 2) (Figure 4C).

We developed a rapid-response assay to get an initial estimate of organoid sensitivity/resistance to VP (Figure 4D). Cisplatin and carboplatin have highly similar mechanisms of action, with highly correlated sensitivity/resistance profiles *in vitro*.[48] Clinically, cisplatin and carboplatin are both used to treat breast cancer and have similar efficacy, while carboplatin reportedly has less toxicity than cisplatin.[49] Given its greater chemical stability and higher potency than carboplatin,[49] we chose cisplatin as the platinum backbone in the drug experiments. In the rapid-response assay, we tested evaluable organoid cultures with a fixed combination dose of 10 μM cisplatin plus 20 μM veliparib. We assessed the combination of relative cell death (quantified using CellTox) and relative organoid area/growth (by live-cell brightfield imaging) in response to VP compared with control and found that the response patterns of most organoids were generally concordant with model predictions, with predicted sensitive organoids demonstrating greater treatment response than predicted resistant organoids in the majority of cases.

We next selected four representative organoid cultures spanning the range of predicted VP responses for more detailed characterization of treatment response. Cell viability data acquired using CellTiter-Glo (Figure 4E) and secondary CellTox and Brightfield imaging results (Figure 4F) demonstrated that model-predicted resistant organoid TORG40 and predicted intermediate organoid TORG58 were resistant to cisplatin monotherapy and VP combination therapy, whereas model-predicted sensitive organoid TORG146 was sensitive to cisplatin with or without veliparib. TORG139 exhibited an intermediate *in vitro* response, consistent with its predicted score. Altogether, *in vitro* responses were broadly concordant with predictions generated from the clinical-biomarker-based model.

Organoid drug screen identifies strategies to overcome resistance to cisplatin

High-throughput screening with a fixed-dose backbone therapy ("anchor" drugs) is useful for identifying combination therapies.[50] With the aim of finding alternative treatment strategies to overcome resistance to platinum chemotherapy, we selected TORG40, a robust, TN/immune$^-$/DRD$^-$ culture with the highest predicted and subsequently validated resistance to VP, and performed a high-throughput drug screen of 386 small-molecule inhibitors at 10 doses in the presence and absence of a fixed concentration of cisplatin to find synergistic or additive small molecule inhibitors with cisplatin (median Z′ factor of the screen was 0.788; interquartile range 0.246–0.837[51]). Based on the dose-response curves of cisplatin in TORG40 (resistant) and TORG146 (sensitive), 5 μM cisplatin was selected as the fixed low-dose backbone concentration for the organoid drug screens (Figure S5).

To identify the top hits in our drug screen, we first ranked the 386 drugs by largest difference in area under the curve (AUC) of the dose-response curve of the drug without cisplatin compared with the drug with cisplatin (Figure 5A). We found that for 20/386 (5.2%) of the tested compounds, the AUC with cisplatin decreased by 40% or more compared with monotherapy, indicating increased efficacy with cisplatin. Seventy-six of three hundred eighty-six (19.7%) had a decrease in AUC between 20% and 40%, and the remaining 290/386 (75.1%) had less than 10% to no change in AUC, or an increase in AUC with cisplatin, indicating no important improvement of efficacy with the treatment combination compared with the monotherapy.

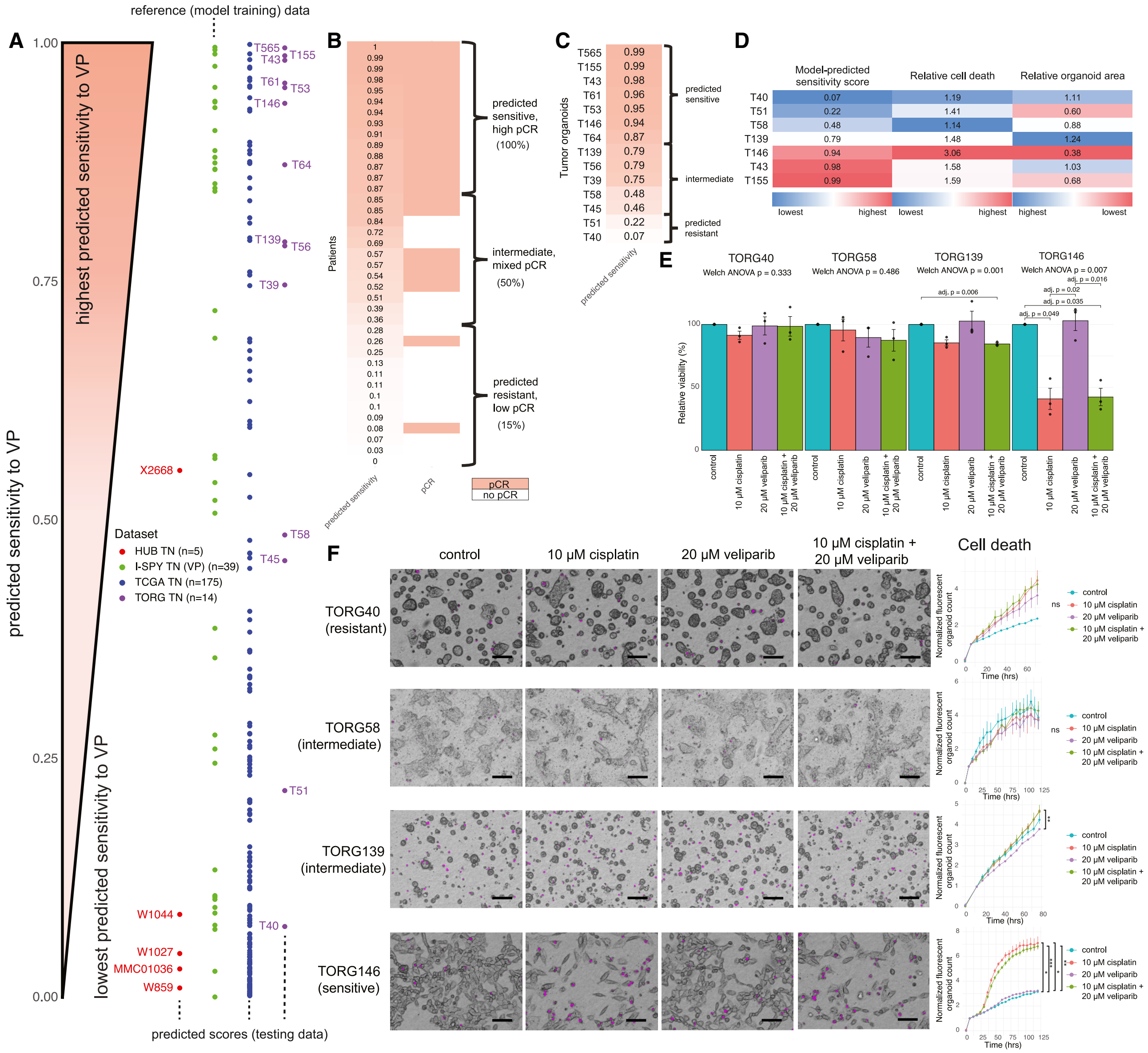

Figure 4. *In vitro* evaluation of the predictive model of treatment response

hrs, hours; HUB, Hubrecht Organoid Technology; TCGA, The Cancer Genome Atlas; ns, not significant.

(A) Ranking of organoids from the highest predicted sensitivity score to the lowest predicted sensitivity score using the predictive model of treatment response to veliparib-platinum chemotherapy (VP) in TNBC, developed using data from patients treated with VP in the I-SPY2 trial. Prediction scores across these reference data (green), triple-negative TCGA tumors (blue), HUB TNBC organoids (red), and TORG TNBC organoids (purple) span the full range of predicted sensitivity to VP.

(B) Ranking of TN I-SPY2 patients treated with VP by predicted sensitivity scores to VP using the predictive model and division in tertiles distinguish a sensitive (n = 14/39; 36%), intermediate (n = 12/39; 31%), and resistant group (n = 13/39; 33%), as confirmed by pathologic complete response (pCR) outcomes (pCR rates 100%, 50%, and 15%, respectively).

(C) Ranking of predicted sensitivity scores to VP in TNBC TORG using the predictive model of treatment response to VP in TNBC also divides the organoids into a predicted sensitive (n = 7/14; 50%), predicted intermediate (n = 5/14; 36%), and predicted resistant group (n = 2/14; 14%) based on the threshold values identified in the I-SPY2 VP-TNBC dataset.

(D) Heatmap showing rapid-response assay readouts used to obtain an initial estimate of organoid sensitivity/resistance to VP in evaluable organoids (n = 7). Scores represent the ratio of relative cell death (quantified using CellTox) and relative organoid area (by live-cell brightfield imaging) in response to VP compared with control at the essay endpoint.

(E) In-depth *in vitro* evaluation of the predicted response to VP by the mathematical model in a predicted resistant (TORG40), two predicted intermediate (TORG58 and TORG139), and one predicted sensitive organoid (TORG146), using a luminescence-based cell viability assay (CellTiter-Glo), shows results concordant with

(legend continued on next page)

The difference in AUCs between the condition without cisplatin and with cisplatin was used to rank the compounds and select the top 5% of compounds in the screen that showed the largest reduction in AUC with the addition of 5 μM cisplatin (n = 19). Seventeen of nineteen (89%) compounds were also among the top 20% of compounds with the smallest IC50 value when given in combination with cisplatin. From these, we selected eight compounds with various mechanisms of action (HDAC inhibitors [epigenetics], Bcl-2 family inhibitors [apoptosis], an inhibitor of DNA synthesis [DNA damage repair], an IAP inhibitor [apoptosis], and an aurora kinase inhibitor [cell-cycle regulation]), with a maximum of three compounds per drug target (individual dose-response curves shown for each drug, with and without cisplatin, in the original drug screen in Figure 5B, with additional details provided in Table S8). We validated these compounds with and without 5 μM cisplatin in the same organoid culture (TORG40) using live-cell brightfield imaging and CellTox. We confirmed an additive effect of cisplatin with all tested compounds at at least one concentration level, as quantified by an increase in total normalized fluorescent (dead) object count in the combination treatment with cisplatin versus monotherapy, along with matching morphological changes by brightfield imaging (Figure 5C).

Next, we sought to validate the clinically most promising drug combination of cisplatin + a Bcl-2/Bcl-xL inhibitor (ABT-263) in other intermediate or resistant organoid cultures. In TORG58 and TORG139, we observed a similar trend of increased efficacy of ABT-263 + cisplatin over ABT-263 monotherapy, measured as an apparent decrease in viable organoid morphology by brightfield (Figure 5D), numerically increased cell death by CellTox (Figure 5E), and decreased cell viability by CellTiter-Glo (Figure 5F). These exploratory single-dose experiments led us to perform formal synergy experiments in all three organoids by testing increasing doses of cisplatin in combination with increasing doses of ABT-263 to explore the additive effects observed in single-dose experiments and identify dose combinations where a synergistic effect could be observed (Figure 5G). Bliss synergy analysis results revealed mean Bliss synergy scores between 11.02 and 31.57 ($p < 0.05$), with the strongest effects observed at 5–10 μM cisplatin doses, suggesting probable synergy between ABT-263 and cisplatin.[52] These results highlight the Bcl-2 family as a promising drug target across multiple models of resistance to VP.

Organoid drug screen reveals compounds with single-agent efficacy for preclinical evaluation

Besides combinatorial treatments that overcome resistance to platinum chemotherapy, we aimed to identify drug classes that were highly effective as a monotherapy. As introduced earlier, the I-SPY2 trial has recently developed the RPS framework, a gene-expression-based classification schema that combines immune, DRD, and luminal-like biological phenotypes with hormone receptor and HER2 status to define clinically relevant breast cancer subgroups.[5] The subgroup of patients with $HER2^-/immune^-/DRD^-$ tumors had particularly low pCR rates and the highest unmet clinical need. Our $TN/immune^-/DRD^-$ organoids model the TN component of this broader biomarker-defined subgroup. One of the most potent drug classes identified in the organoid drug screen was HSP90 inhibitors (Figures 6A and 6B). HSP90 is an abundant chaperone protein involved in the folding of oncoproteins under basal conditions and during stress response. We tested ganetespib, one of the most clinically advanced HSP90 inhibitors, in two other $TN/immune^-/DRD^-$ organoids and confirmed the high single-agent potency of this drug in organoids of this subtype (Figures 6C–6E).

Clinically, the HSP90 inhibitor ganetespib has been tested in the neoadjuvant I-SPY2 clinical trial in combination with standard neoadjuvant chemotherapy in HR^+HER2^- and TNBC. Ganetespib showed numerically, but not statistically significantly, improved pCR in TNBC compared with standard neoadjuvant chemotherapy alone.[53] Given the potent drug effect *in vitro*, we retrospectively studied the ganetespib arm in I-SPY2 using the current RPS framework and extended follow-up data, focusing primarily on the $HER2^-/immune^-/DRD^-$ subgroup, while also exploring the smaller $TN/immune^-/DRD^-$ subset separately, given the continued widespread use of TNBC classifications in the field. Within the subgroup of patients classified as $HER2^-/immune^-/DRD^-$ by the RPS framework (N = 224), 38 patients had received neoadjuvant ganetespib (150 mg/m^2 ganetespib every 3 weeks with paclitaxel, then doxorubicin plus cyclophosphamide [AC]), and 186 patients had received standard neoadjuvant chemotherapy control (paclitaxel-AC) (Figure 6F). pCR rates were similarly low in the ganetespib arm and the control arm (13% vs. 9%, respectively; Fisher's exact test, p = 0.55). When assessing distant recurrence-free survival (DRFS), we observed improved DRFS outcomes with ganetespib compared with control (Figure 6G, p = 0.008). In an exploratory analysis of the $TN/immune^-/DRD^-$ subset (n = 47), we observed a similar trend (Figure 6H). Though exploratory and limited by small sample sizes, these analyses support further investigation of HSP90 inhibition in $HER2^-/immune^-/DRD^-$ breast cancers identified through the RPS framework.

DISCUSSION

Here, we describe the application of a predictive model obtained from clinical patient biomarker data to predict treatment response or resistance in organoids. We used this model to

response predictions. Bars show mean relative cell viability (%) compared to untreated control, with error bars showing the SEM of three biological replicates per organoid, each with three technical replicates. p values by Welch's ANOVA, followed by the Games-Howell test.

(F) Live-cell imaging results corresponding with the experiments in Figure 5E illustrate the lack of cell death after treatment with 10 μM cisplatin + 20 μM veliparib in TORG40 (predicted resistant) and TORG58 (predicted intermediate), whereas TORG139 (predicted intermediate) showed an intermediate result, and significant cell death was observed in TORG146 (predicted sensitive). Purple color indicates fluorescence from a cell-death dye (CellTox), as captured by the IncuCyte, with quantification in the right panels (fluorescent object count normalized to the first stable fluorescence recording [T = 6 h]). Data are expressed as the mean ± SEM of three technical replicates within a single experiment. Data representative of three independent biological replicates per organoid. ***$p < 0.001$, **$p < 0.01$, *$p < 0.05$ by Games-Howell test following Welch's ANOVA. Scale bars: 100 μm.

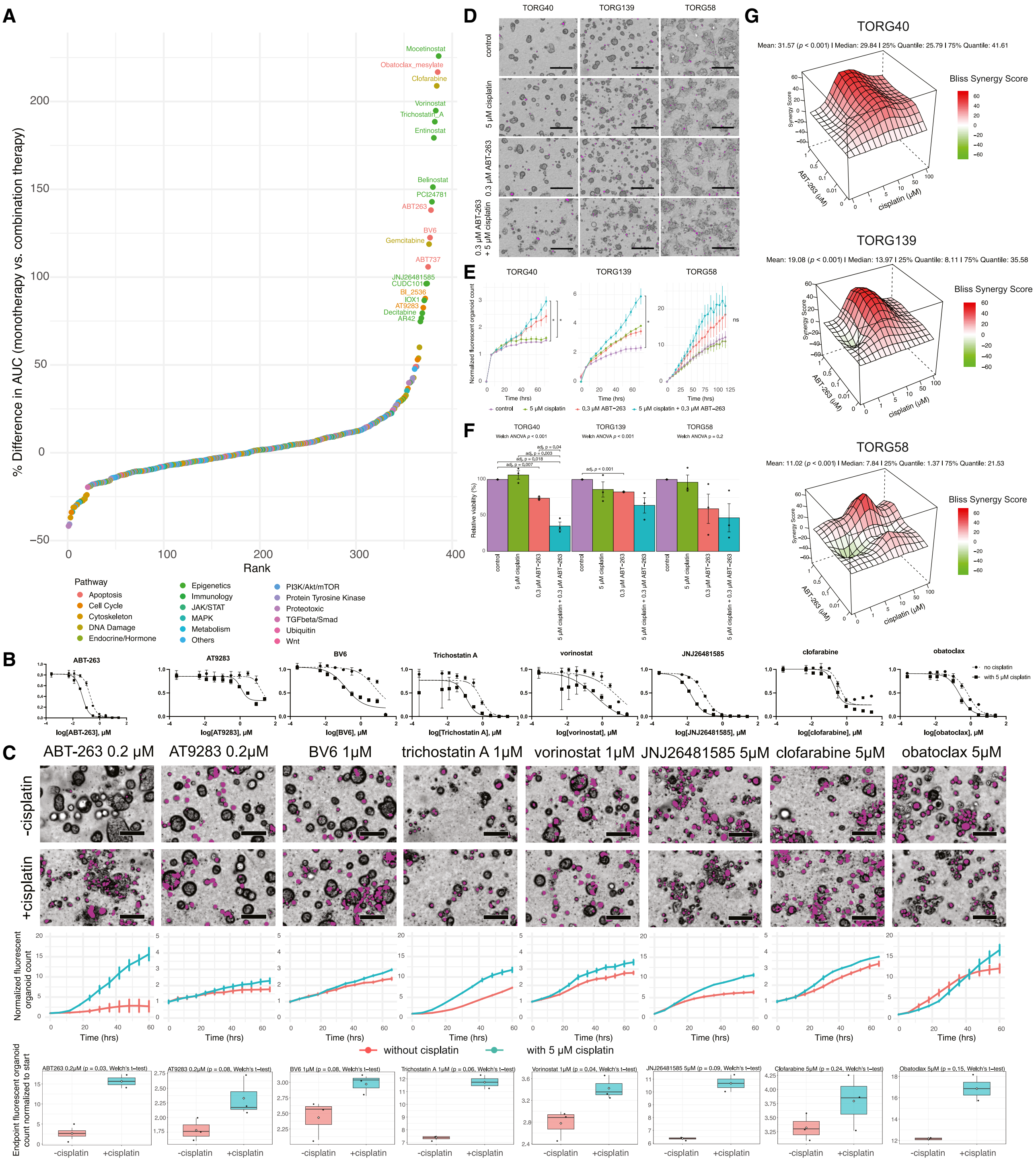

Figure 5. A high-throughput drug screen in a tumor organoid resistant to veliparib-platinum chemotherapy (VP) reveals treatment strategies to overcome resistance to platinum chemotherapy

See also Figure S5. hrs, hours. ns, not significant.

(A) Ranking of 386 drugs in high-throughput drug screen by the percent difference in area under the curve (AUC) of the dose-response curve of the drug compound without cisplatin (monotherapy) compared with the AUC of the drug with cisplatin (combination therapy). Each dot represents one drug in the drug screen, sorted by increasing rank number, with the highest rank = the highest difference. The top 5% of compounds with the largest reduction in AUC with cisplatin are labeled with the drug name. The colors of the dots correspond with the mechanism of action of the drug.

(legend continued on next page)

select biomarker-matched treatment-resistant organoid cultures from a biobank and tested alternative treatment strategies. Moreover, we make available a data resource (Table S9) that can be used to study biomarkers and mechanisms of treatment resistance in residual disease breast cancer organoids.

This proof-of-principle study illustrates the potential of breast cancer organoids to bridge the gap between correlative clinical science and experimental biology. While earlier studies have correlated cell line features with *in vitro* drug sensitivity,[54] and other studies have shown concordance in expression of focused sets of biomarkers between patient-derived organoids (PDOs) and patient-derived xenografts (PDXs),[6,7,55] experimental validations of patient-tissue-derived biomarkers in preclinical models are still scarce.[56,57] With the immense rise of genomics studies and increasing numbers of predictive biomarkers, there is a need to translate *in silico* transcriptomic findings to functional *in vitro* tools that can then be used to test or stratify large numbers of investigational therapies.

To this end, we utilized gene expression from an existing organoid biobank and developed a second set of TNBC organoids, derived from both pre-treatment and post-treatment tissues, covering a wide range of histological subtypes and including rare subtypes that are associated with treatment resistance that are often overlooked, such as metaplastic and inflammatory breast cancers. The biobank also included organoids classified as "normal-type" by PAM50. We hypothesize that the "normal-type" classification could be explained by either LAR tumors or heterogeneity of the original tumor tissue, where admixed populations of cells may not be represented well by a bulk analysis technique and a categorical classification. Interestingly, another subset of TNBC organoids was particularly aggressive in culture, as characterized by fast growth *in vitro*, high expression of MYC and proliferation signatures, and more extensive genomic copy-number aberrations. They represent a subgroup of breast cancers in exceptionally high need of adequate treatment.

We modeled organoid resistance states using qualifying gene-expression-based biomarkers and clinical data from the I-SPY2 trial. These qualifying biomarkers were pre-specified for prospective testing in I-SPY2 for their specific response-predictive value based on existing evidence suggesting a role in treatment response prediction,[5] making them especially interesting and meaningful to evaluate in organoid models. The most significant model using multiple biomarkers expressed in our organoid models and correlating with clinical outcomes in patients with TNBC in the I-SPY2 trial was a model that predicted response to veliparib-platinum chemotherapy. This is a particularly clinically relevant model, since platinum chemotherapies are often prescribed to patients with TNBC,[58–61] but non-response is frequent, and more approaches to overcome platinum-chemotherapy resistance are needed. Moreover, this model exemplifies that DNA damage-response genes (in the PARPi7 signature) can predict response to chemotherapy and/or PARP inhibition at the gene expression level, not only in patient tissues[5] but also in organoids. The organoids with the highest predicted and validated resistance to platinum chemotherapy plus veliparib were mostly basal-like organoids, suggesting that findings from our drug screen may be most applicable to patients with basal-like tumors.

As multiple clinical studies, including I-SPY2, have established platinum therapy as part of the standard of care for the treatment of TNBC,[5,36,62,63] we chose to focus our drug screen on overcoming resistance to platinum chemotherapy. Using our predictive model, we validated and selected robust veliparib-platinum chemotherapy (VP)-resistant organoid models for downstream testing. The drug screen identified HDAC inhibitors, Bcl-2 family inhibitors, and IAP inhibitors as most effective when combined with cisplatin, with a similar trend observed with the Bcl-2 family inhibitor ABT-263 in two other organoid models with relative resistance to VP. Ongoing clinical trials in breast cancer are assessing combination therapies of Bcl-2 family inhibitors with PARP inhibitors and other experimental agents, but not yet with platinum chemotherapies.[64,65] Our study suggests this strategy should be tested further to overcome platinum resistance in aggressive TNBC subtypes.

Our screen also revealed HSP90 as a potential treatment target. Prior reports have established that HSP90 is a chaperone for several client proteins that can promote metastasis and support stem cell maintenance.[66–68] Though this analysis of DRFS in I-SPY2 was exploratory and hypothesis-generating, limited by the retrospective nature, small sample size, lack of covariate adjustment, and receptor subtype imbalance, these findings

(B) Average drug dose-response curves for a selection of the top combination therapies in the 386-compound drug screen (solid line indicating the average dose-response curve for the drug + 5 μM cisplatin and dotted line indicating the average for the drug as a monotherapy) in TORG40 determined at T = 72 h using a CellTiter-Glo. Data are expressed as the mean ± SEM of two technical replicates. See also Table S8.

(C) Validation of the top combination treatments from the drug screen in TORG40 using a secondary, imaging-based assay in TORG40. Purple color indicates fluorescence from a cell-death dye (CellTox). Graphs show total fluorescent object count at the final time point normalized to the starting point (T = 0), with the blue lines/bars indicating the drug + 5 μM cisplatin, and the pink lines/bars indicating the drug as a monotherapy. Data are expressed as the mean ± SEM of 2–3 technical replicates. Scale bars: 100 μm. p values by Welch's t test.

(D) Validation of the cisplatin + ABT-263 condition in two other *in vitro* intermediate/resistant cultures to VP by brightfield and fluorescent imaging. Purple color indicates fluorescence from a cell-death dye (CellTox). Scale bars: 100 μm.

(E) Cell death curves corresponding with images in Figure 5D, showing the effects of ABT-263 with cisplatin as quantified using CellTox (total fluorescent object count at endpoint normalized to the first stable fluorescence recording [T = 6 h]). Data are expressed as the mean ± SEM of three technical replicates within a single experiment. Data representative of three independent biological replicates per organoid. $^{*}p$ value < 0.05 by Games-Howell test following Welch's ANOVA.

(F) Validation of the cisplatin + ABT-263 treatment combination in two other *in vitro* intermediate/resistant cultures to VP using a cell viability assay (CellTiter-Glo) suggests an additive effect of cisplatin to ABT-263. Bars show mean relative cell viability (%), compared to the untreated control ± SEM of three biological replicates per organoid, each with three technical replicates. p values by Welch's ANOVA, followed by the Games-Howell test.

(G) 3D plots showing the Bliss synergy analysis results for ABT-263 with cisplatin for TORG40, TORG139, and TORG58 within one experiment. Data representative of two independent biological replicates per organoid. The organoids have mean Bliss synergy scores >10 (p value < 0.05), suggesting probable synergy between ABT-263 and cisplatin, particularly at 5–10 μM cisplatin doses.

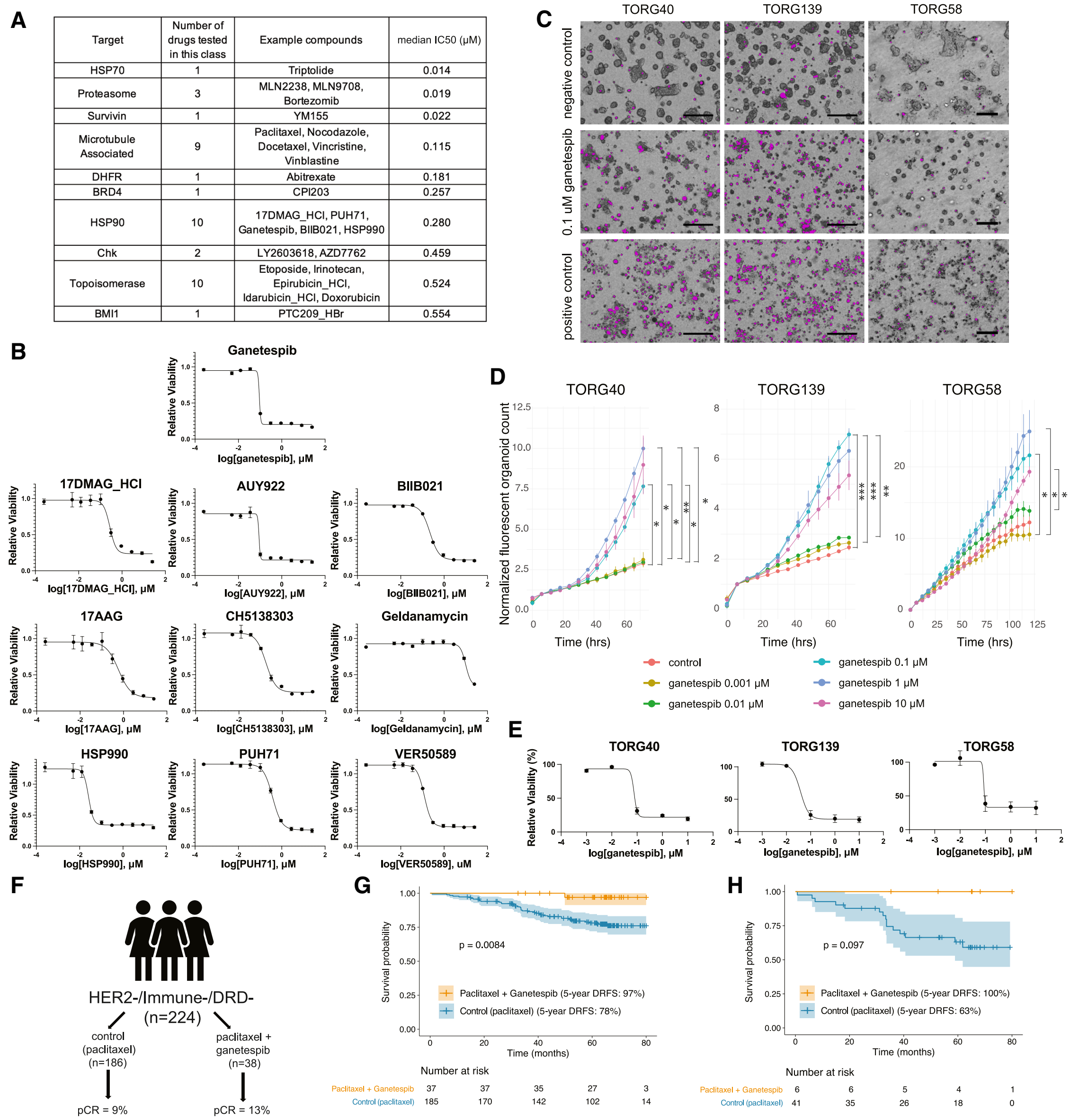

Target	Number of drugs tested in this class	Example compounds	median IC50 (μM)
HSP70	1	Triptolide	0.014
Proteasome	3	MLN2238, MLN9708, Bortezomib	0.019
Survivin	1	YM155	0.022
Microtubule Associated	9	Paclitaxel, Nocodazole, Docetaxel, Vincristine, Vinblastine	0.115
DHFR	1	Abitrexate	0.181
BRD4	1	CPI203	0.257
HSP90	10	17DMAG_HCl, PUH71, Ganetespib, BIIB021, HSP990	0.280
Chk	2	LY2603618, AZD7762	0.459
Topoisomerase	10	Etoposide, Irinotecan, Epirubicin_HCl, Idarubicin_HCl, Doxorubicin	0.524
BMI1	1	PTC209_HBr	0.554

Figure 6. Compounds with single-agent efficacy in TNBC organoid drug screen improve survival in I-SPY2

BRD4, bromodomain-containing protein 4; DHFR, dihydrofolate reductase; DRD, DNA repair deficiency; DRFS, distant recurrence-free survival; hrs, hours; HSP, heat shock protein; pCR, pathologic complete response; TN, triple-negative.

(A) Top 10 drug classes that were most effective as a monotherapy in the organoid drug screen based on the median half maximal inhibitory concentration (IC50)-value (μM) across the drug class. HSP90 inhibitors are among the largest drug classes evaluated in the screen.

(B) Individual dose-response curves for the 10 HSP90 inhibitors tested in the screen, showing consistently small IC50 values with the exception of one compound (geldanamycin). Error bars show SEM of two technical replicates.

(C) Validation of ganetespib efficacy in two additional TN/immune$^-$/DRD$^-$ organoids. Purple color indicates fluorescence from a cell-death dye (CellTox). Scale bars: 200 μm.

(D) Quantification of fluorescent (dead) object count using a cell-death dye (CellTox), corresponding to images in Figure 6C, normalized to the first stable fluorescence recording (T = 6 h). Data are expressed as the mean ± SEM of three technical replicates within a single experiment. Data representative of two independent biological replicates per organoid. ***$p < 0.001$, **$p < 0.01$, *$p < 0.05$ by Games-Howell test following Welch's ANOVA (significant results shown).

(E) Dose-response curves of ganetespib in two other TN/immune$^-$/DRD$^-$ organoids. The maximum drug effect appears to be achieved at 0.1–1 μM, with ~20% residual cell viability compared to the control. Bars show mean relative cell viability by CellTiter-Glo (%), compared to the untreated control ± SEM of two biological replicates per organoid, each with three technical replicates.

(F) Overview of I-SPY2 clinical trial data for the control and ganetespib arms in patients with HER2$^-$/immune$^-$/DRD$^-$ breast cancer.

(G) Kaplan-Meier analysis (log rank test) of DRFS in patients with HER2-/immune$^-$/DRD$^-$ tumors treated with control vs. ganetespib in the I-SPY2 trial.

(H) Exploratory Kaplan-Meier analysis (log rank test) of DRFS in the subset of patients with TN/immune$^-$/DRD$^-$ treated with control vs. ganetespib in the I-SPY2 trial.

CellPress
OPEN ACCESS

provide rationale for further evaluation of HSP90 inhibition in this high-need patient population.[5]

Most compounds assessed in our compound screen and in the I-SPY2 clinical trial were not primarily directed against metabolic pathways. As prior reports have suggested metabolic differences between organoids and primary tumors,[69] our study should be viewed primarily as a proof of concept rather than a comprehensive assessment across all therapeutic categories. The extent to which metabolic differences and other culture-associated adaptations may impact drug responses, particularly for therapies targeting metabolic dependencies, was not assessed in this study and remains an important future direction.

In summary, by combining computational analyses of large molecular and clinical datasets with organoid model systems, this proof-of-principle study demonstrated the utility of matching I-SPY2 resistance biomarkers and signatures to residual disease tumor organoid cultures. This research showed that tumor organoid cultures can be utilized in the laboratory to mimic drug resistance states and serve as a valuable complement to existing research models of breast cancer for preclinical drug testing informed by tumor biology. Our findings highlight the value of a reverse translational approach that integrates patient-level clinical trial data with *in silico* and *ex vivo* testing in organoid models to generate translational hypotheses.[70]

Limitations of the study

These findings should be viewed with consideration of the study's limitations. Given the modest cohort sizes, validation of our findings in independent cohorts is required. Not all patient specimens collected in this study yielded molecularly confirmed tumor organoids suitable for downstream analyses; thus, there is an establishment bias that affects generalizability. The organoid cultures lacked immune and stromal components, restricting analysis to biomarkers evaluable in epithelial tumor cells. Future studies using immune-competent organoid systems may enable modeling of additional treatment types, including immunotherapy. Moreover, our study utilized bulk gene expression and thus did not resolve intratumoral heterogeneity. Single-cell approaches could help delineate contributions from different cellular subpopulations in future studies. The predictive model was derived using a pragmatic stepwise selection procedure and only organoid-evaluable markers as input, potentially limiting the robustness and generalizability of the final model in other patient populations and treatment settings.

RESOURCE AVAILABILITY

Lead contact

Further information and requests for resources and data should be directed to and will be fulfilled by the lead contact, Jennifer M. Rosenbluth (jennifer.rosenbluth@ucsf.edu).

Materials availability

Organoid lines from this biobank and other experimental data are available upon reasonable request from the lead contact. Details on reagents and resources are included in the key resources table.

Data and code availability

- Deposited data: raw RNA-sequencing data (FASTQ files) and processed RSEM files for TNBC TORG gene expression analysis are publicly available to download from NCBI Gene Expression Omnibus (GEO) under accession number GEO: GSE303509.
- This manuscript does not report original computer code, algorithms, or computational models.
- Any additional information required to reanalyze the data reported in this work paper is available from the lead contact upon request.

ACKNOWLEDGMENTS

The authors thank Hans Clevers for 293T-Noggin and L-Wnt3a cell lines, Calvin Kuo for 293T-RSPO1, and Joan Brugge and Laura Selfors for their helpful discussions and advice. We thank Nadine Goldhammer, Arianna Zhu, Mackenzie Boedicker, Griffin Boedicker, Alexis Cook, and Pravin Phadatare for technical assistance; the Inflammatory Breast Cancer Program at Dana-Farber Cancer Institute; the DF/HCC Breast SPORE: Specialized Program of Research Excellence (SPORE) (NCI 1P50CA168504); and the UCSF Breast Care Center surgical teams including Jasmine Wong and the Breast Care Center interns for assistance with tissue specimen. We further thank the ICCB-Longwood Screening Facility at Harvard Medical School and the Wynton HPC Co-Op cluster, which is supported by UCSF research faculty and UCSF institutional funds. We thank Tempus AI for assistance with DNA sequencing data acquisition. Funding was from the National Institutes of Health (under award number R01CA281361), the Susan G. Komen Foundation, METAvivor (to J.M.R.), and Breast Cancer Foundation (to L.J.v.'t.V. and J.M.R.). T.B.V.B. was supported by the ROSANNA Fund, Hendrik Muller Fund, Netherland-America Foundation, the Foundation of Renswoude, and the Nijbakker-Morra Foundation. J.M.R. is a Chan-Zuckerberg Biohub – San Francisco investigator. The content is solely the responsibility of the authors and does not necessarily represent the official views of the NIH/NCI.

AUTHOR CONTRIBUTIONS

Conceptualization, J.M.R., T.B.V.B., L.J.v.'t.V., and D.M.W.; data curation, T.B.V.B., D.M.W., C.Y., I.J.N., M.C.B., and J.L.; data analysis, T.B.V.B. and D.M.W.; funding acquisition, J.M.R.; investigation: T.B.V.B., J.M.R., D.M.W., K.M., M.C.B., S.W., A.P., S.D.W.C., and J.L.; methodology, T.B.V.B., J.M.R., D.M.W., and I.S.H.; project administration, T.B.V.B. and J.M.R.; software, I.S.H. and D.M.W.; sample acquisition/resources, J.M.R., D.D., B.O., F.L., I.J.N., D.M.W., G.L.H., L.B.-S., L.J.E., and L.J.v.'t.V.; supervision, J.M.R., L.J.v.'t.V., B.M.T.B.; validation, T.B.V.B., D.M.W., M.C.B., S.D.W.C., and J.L.; visualization, T.B.V.B., D.M.W., J.L., and S.D.W.C.; writing – original draft, T.B.V.B., M.C.B., and J.M.R.; writing – review and editing: all authors.

DECLARATION OF INTERESTS

L.J.v.'t.V. is a part-time employee and stockholder of Agendia. L.J.E. reported that she is an uncompensated board member of the Quantum Leap Healthcare Collaborative, which sponsors the I-SPY trial.

STAR★METHODS

Detailed methods are provided in the online version of this paper and include the following:

- KEY RESOURCES TABLE
- EXPERIMENTAL MODEL AND STUDY PARTICIPANT DETAILS
  - HUB Living Biobank data
  - I-SPY2 trial data
  - TNBC TORG biobank
- METHOD DETAILS
  - Predictive models of treatment response derived from the I-SPY2 trial biomarker resource
  - Organoid embedding and staining (hematoxylin & eosin staining, immunohistochemistry)
  - Organoid DNA and RNA extraction
  - DNA sequencing and analysis
  - Breast cancer organoid validation and quality control

- ○ RNA sequencing and analysis
- ○ Merge of organoid data with TCGA
- ○ Analysis of 100 biomarker gene sets
- ○ Sensitivity testing to cisplatin-veliparib
- ○ Small molecule drug screen
- ○ Validation of top combination hits using a secondary, imaging-based assay
- ○ Validation of top hits in other intermediate/resistant organoid cultures
- ○ Synergy testing
- ○ Distant-recurrence free survival analysis in I-SPY2 clinical trial data
- QUANTIFICATION AND STATISTICAL ANALYSIS
- ADDITIONAL RESOURCES

SUPPLEMENTAL INFORMATION

Supplemental information can be found online at https://doi.org/10.1016/j.xcrm.2026.102973.

Received: October 30, 2025
Revised: May 26, 2026
Accepted: July 10, 2026
Published: August 6, 2026

REFERENCES

1. Gao, H., Korn, J.M., Ferretti, S., Monahan, J.E., Wang, Y., Singh, M., Zhang, C., Schnell, C., Yang, G., Zhang, Y., et al. (2015). High-throughput screening using patient-derived tumor xenografts to predict clinical trial drug response. Nat. Med. *21*, 1318–1325. https://doi.org/10.1038/nm.3954.
2. Baker, B.M., and Chen, C.S. (2012). Deconstructing the third dimension-how 3D culture microenvironments alter cellular cues. J. Cell Sci. *125*, 3015–3024. https://doi.org/10.1242/jcs.079509.
3. Sachs, N., de Ligt, J., Kopper, O., Gogola, E., Bounova, G., Weeber, F., Balgobind, A.V., Wind, K., Gracanin, A., Begthel, H., et al. (2018). A Living Biobank of Breast Cancer Organoids Captures Disease Heterogeneity. Cell *172*, 373–386.e10. https://doi.org/10.1016/j.cell.2017.11.010.
4. Bhatia, S., Kramer, M., Russo, S., Naik, P., Arun, G., Brophy, K., Andrews, P., Fan, C., Perou, C.M., Preall, J., et al. (2022). Patient-Derived Triple-Negative Breast Cancer Organoids Provide Robust Model Systems That Recapitulate Tumor Intrinsic Characteristics. Cancer Res. *82*, 1174–1192. https://doi.org/10.1158/0008-5472.CAN-21-2807.
5. Wolf, D.M., Yau, C., Wulfkuhle, J., Brown-Swigart, L., Gallagher, R.I., Lee, P.R.E., Zhu, Z., Magbanua, M.J., Sayaman, R., O'Grady, N., et al. (2022). Redefining breast cancer subtypes to guide treatment prioritization and maximize response: Predictive biomarkers across 10 cancer therapies. Cancer Cell *40*, 609–623.e6. https://doi.org/10.1016/j.ccell.2022.05.005.
6. Vlachogiannis, G., Hedayat, S., Vatsiou, A., Jamin, Y., Fernández-Mateos, J., Khan, K., Lampis, A., Eason, K., Huntingford, I., Burke, R., et al. (2018). Patient-derived organoids model treatment response of metastatic gastrointestinal cancers. Science *359*, 920–926. https://doi.org/10.1126/science.aao2774.
7. Wang, R., Mao, Y., Wang, W., Zhou, X., Wang, W., Gao, S., Li, J., Wen, L., Fu, W., and Tang, F. (2022). Systematic evaluation of colorectal cancer organoid system by single-cell RNA-Seq analysis. Genome Biol. *23*, 106. https://doi.org/10.1186/s13059-022-02673-3.
8. Lenin, S., Ponthier, E., Scheer, K.G., Yeo, E.C.F., Tea, M.N., Ebert, L.M., Oksdath Mansilla, M., Poonnoose, S., Baumgartner, U., Day, B.W., et al. (2021). A drug screening pipeline using 2d and 3d patient-derived in vitro models for pre-clinical analysis of therapy response in glioblastoma. Int. J. Mol. Sci. *22*, 4322. https://doi.org/10.3390/ijms22094322.
9. Mertens, S., Huismans, M.A., Verissimo, C.S., Ponsioen, B., Overmeer, R., Proost, N., van Tellingen, O., van de Ven, M., Begthel, H., Boj, S.F., et al. (2023). Drug-repurposing screen on patient-derived organoids identifies therapy-induced vulnerability in KRAS-mutant colon cancer. Cell Rep. *42*, 112324. https://doi.org/10.1016/j.celrep.2023.112324.
10. Ooft, S.N., Weeber, F., Dijkstra, K.K., Mclean, C.M., Kaing, S., Van Werkhoven, E., Schipper, L., Hoes, L., Vis, D.J., Van De Haar, J., et al. (2019). Patient-derived organoids can predict response to chemotherapy in metastatic colorectal cancer patients. Sci. Transl. Med. *11*, 2574. https://doi.org/10.1126/scitranslmed.aay2574.
11. Guillen, K.P., Fujita, M., Butterfield, A.J., Scherer, S.D., Bailey, M.H., Chu, Z., DeRose, Y.S., Zhao, L., Cortes-Sanchez, E., Yang, C.H., et al. (2022). A human breast cancer-derived xenograft and organoid platform for drug discovery and precision oncology. Nat. Cancer *3*, 232–250. https://doi.org/10.1038/s43018-022-00337-6.
12. Vilgelm, A.E., Bergdorf, K., Wolf, M., Bharti, V., Shattuck-Brandt, R., Blevins, A., Jones, C., Phifer, C., Lee, M., Lowe, C., et al. (2020). Fine-Needle Aspiration-Based Patient-Derived Cancer Organoids. iScience *23*, 101408. https://doi.org/10.1016/j.isci.2020.101408.
13. Driehuis, E., Van Hoeck, A., Moore, K., Kolders, S., Francies, H.E., Gulersonmez, M.C., Stigter, E.C.A., Burgering, B., Geurts, V., Gracanin, A., et al. (2019). Pancreatic cancer organoids recapitulate disease and allow personalized drug screening. Proc. Natl. Acad. Sci. USA *116*, 26580–26590. https://doi.org/10.1073/pnas.1911273116.
14. Saito, Y., Muramatsu, T., Kanai, Y., Ojima, H., Sukeda, A., Hiraoka, N., Arai, E., Sugiyama, Y., Matsuzaki, J., Uchida, R., et al. (2019). Establishment of Patient-Derived Organoids and Drug Screening for Biliary Tract Carcinoma. Cell Rep. *27*, 1265–1276.e4. https://doi.org/10.1016/j.celrep.2019.03.088.
15. Pauli, C., Hopkins, B.D., Prandi, D., Shaw, R., Fedrizzi, T., Sboner, A., Sailer, V., Augello, M., Puca, L., Rosati, R., et al. (2017). Personalized in vitro and in vivo cancer models to guide precision medicine. Cancer Discov. *7*, 462–477. https://doi.org/10.1158/2159-8290.CD-16-1154.
16. Hou, S., Tiriac, H., Sridharan, B.P., Scampavia, L., Madoux, F., Seldin, J., Souza, G.R., Watson, D., Tuveson, D., and Spicer, T.P. (2018). Advanced Development of Primary Pancreatic Organoid Tumor Models for High-Throughput Phenotypic Drug Screening. SLAS Discov. *23*, 574–584. https://doi.org/10.1177/2472555218766842.
17. Phan, N., Hong, J.J., Tofig, B., Mapua, M., Elashoff, D., Moatamed, N.A., Huang, J., Memarzadeh, S., Damoiseaux, R., and Soragni, A. (2019). A simple high-throughput approach identifies actionable drug sensitivities in patient-derived tumor organoids. Commun. Biol. *2*, 78. https://doi.org/10.1038/s42003-019-0305-x.
18. Du, Y., Li, X., Niu, Q., Mo, X., Qui, M., Ma, T., Kuo, C.J., and Fu, H. (2020). Development of a miniaturized 3D organoid culture platform for ultra-high-throughput screening. J. Mol. Cell Biol. *12*, 630–643. https://doi.org/10.1093/jmcb/mjaa036.
19. Bergdorf, K., Phifer, C., Bharti, V., Westover, D., Bauer, J., Vilgelm, A., Lee, E., and Weiss, V. (2020). High-throughput drug screening of fine-needle aspiration-derived cancer organoids. STAR Protoc. *1*, 100212. https://doi.org/10.1016/j.xpro.2020.100212.
20. Rao, X., Qiao, Z., Yang, Y., Deng, Y., Zhang, Z., Yu, X., and Guo, X. (2024). Unveiling Epigenetic Vulnerabilities in Triple-Negative Breast Cancer through 3D Organoid Drug Screening. Pharmaceuticals *17*, 225. https://doi.org/10.3390/ph17020225.
21. Högström, J.M., and Muranen, T. (2025). An Optimized Protocol for Simultaneous Propagation of Patient-derived Organoids and Matching CAFs. Bio. Protoc. *15*, e5160. https://doi.org/10.21769/BioProtoc.5160.
22. Hogstrom, J.M., Cruz, K.A., Selfors, L.M., Ward, M.N., Mehta, T.S., Kanarek, N., Philips, J., Dialani, V., Wulf, G., Collins, L.C., et al. (2023). Simultaneous isolation of hormone receptor–positive breast cancer organoids and fibroblasts reveals stroma-mediated resistance mechanisms. J. Biol. Chem. *299*, 105021. https://doi.org/10.1016/j.jbc.2023.105021.
23. Oliphant, M.U.J., Akshinthala, D., and Muthuswamy, S.K. (2024). Establishing conditions for the generation and maintenance of estrogen receptor-positive organoid models of breast cancer. Breast Cancer Res. *26*, 56. https://doi.org/10.1186/s13058-024-01798-6.

CellPress
OPEN ACCESS

24. Chang, K.-C., Silvestri, F., Oliphant, M.U.J., Martinez-Gakidis, M.A., Orgill, D.P., Garber, J.E., Dillon, D.D., and Brugge, J.S. (2024). Breast organoid suspension cultures maintain long-term estrogen receptor expression and responsiveness. NPJ Breast Cancer *10*, 107. https://doi.org/10.1038/s41523-024-00714-7.

25. Uematsu, H., Saito, C., Kondo, J., Onuma, K., Coppo, R., Mori, Y., Muto, M., Kikawa, Y., Tada, M., Sugie, T., and Inoue, M. (2023). De-differentiation in cultures of organoids from luminal-type breast cancer is restored by inhibition of NOTCH signaling. Hum. Cell *36*, 2099–2112. https://doi.org/10.1007/s13577-023-00975-7.

26. Liberzon, A., Birger, C., Thorvaldsdóttir, H., Ghandi, M., Mesirov, J.P., and Tamayo, P. (2015). The Molecular Signatures Database Hallmark Gene Set Collection. Cell Syst. *1*, 417–425. https://doi.org/10.1016/j.cels.2015.12.004.

27. Dushyanthen, S., Beavis, P.A., Savas, P., Teo, Z.L., Zhou, C., Mansour, M., Darcy, P.K., and Loi, S. (2015). Relevance of tumor-infiltrating lymphocytes in breast cancer. BMC Med. *13*, 202. https://doi.org/10.1186/s12916-015-0431-3.

28. Barnes, T.A., and Amir, E. (2017). HYPE or HOPE: The prognostic value of infiltrating immune cells in cancer. Br. J. Cancer *117*, 451–460. https://doi.org/10.1038/bjc.2017.220.

29. Cardoso, F., van't Veer, L.J., Bogaerts, J., Slaets, L., Viale, G., Delaloge, S., Pierga, J.-Y., Brain, E., Causeret, S., DeLorenzi, M., et al. (2016). 70-Gene Signature as an Aid to Treatment Decisions in Early-Stage Breast Cancer. N. Engl. J. Med. *375*, 717–729. https://doi.org/10.1056/nejmoa1602253.

30. Kuilman, M.M., Ellappalayam, A., Barcaru, A., Haan, J.C., Bhaskaran, R., Wehkamp, D., Menicucci, A.R., Audeh, W.M., Mittempergher, L., and Glas, A.M. (2022). BluePrint breast cancer molecular subtyping recognizes single and dual subtype tumors with implications for therapeutic guidance. Breast Cancer Res. Treat. *195*, 263–274. https://doi.org/10.1007/s10549-022-06698-x.

31. Bethesda (MD) (2020). LiverTox: Clinical and Research Information on Drug- Induced Liver Injury. https://www.ncbi.nlm.nih.gov/books/NBK548565/.

32. Rodler, E.T., Kurland, B.F., Griffin, M., Gralow, J.R., Porter, P., Yeh, R.F., Gadi, V.K., Guenthoer, J., Beumer, J.H., Korde, L., et al. (2016). Phase I study of veliparib (ABT-888) combined with cisplatin and vinorelbine in advanced triple-negative breast cancer and/or BRCA mutation-associated breast cancer. Clin. Cancer Res. *22*, 2855–2864. https://doi.org/10.1158/1078-0432.CCR-15-2137.

33. Donawho, C.K., Luo, Y., Luo, Y., Penning, T.D., Bauch, J.L., Bouska, J.J., Bontcheva-Diaz, V.D., Cox, B.F., DeWeese, T.L., Dillehay, L.E., et al. (2007). ABT-888, an orally active poly(ADP-ribose) polymerase inhibitor that potentiates DNA-damaging agents in preclinical tumor models. Clin. Cancer Res. *13*, 2728–2737. https://doi.org/10.1158/1078-0432.CCR-06-3039.

34. Silver, D.P., Richardson, A.L., Eklund, A.C., Wang, Z.C., Szallasi, Z., Li, Q., Juul, N., Leong, C.O., Calogrias, D., Buraimoh, A., et al. (2010). Efficacy of neoadjuvant cisplatin in triple-negative breast cancer. J. Clin. Oncol. *28*, 1145–1153. https://doi.org/10.1200/JCO.2009.22.4725.

35. Byrski, T., Gronwald, J., Huzarski, T., Grzybowska, E., Budryk, M., Stawicka, M., Mierzwa, T., Szwiec, M., Wiśniowski, R., Siolek, M., et al. (2010). Pathologic complete response rates in young women with BRCA1-positive breast cancers after neoadjuvant chemotherapy. J. Clin. Oncol. *28*, 375–379. https://doi.org/10.1200/JCO.2008.20.7019.

36. Geyer, C.E., Sikov, W.M., Huober, J., Rugo, H.S., Wolmark, N., O'Shaughnessy, J., Maag, D., Untch, M., Golshan, M., Lorenzo, J.P., et al. (2022). Long-term efficacy and safety of addition of carboplatin with or without veliparib to standard neoadjuvant chemotherapy in triple-negative breast cancer: 4-year follow-up data from BrighTNess, a randomized phase III trial. Ann. Oncol. *33*, 384–394. https://doi.org/10.1016/j.annonc.2022.01.009.

37. Wolf, D.M., Yau, C., Sanil, A., Glas, A., Petricoin, E., Wulfkuhle, J., Severson, T.M., Linn, S., Brown-Swigart, L., Hirst, G., et al. (2017). DNA repair deficiency biomarkers and the 70-gene ultra-high risk signature as predictors of veliparib/carboplatin response in the I-SPY 2 breast cancer trial. NPJ Breast Cancer *3*, 31. https://doi.org/10.1038/S41523-017-0025-7.

38. UniProt (2025). P83731 · RL24_HUMAN. https://www.uniprot.org/uniprotkb/P83731/entry.

39. National Library of Medicine (2025). RPL24 Ribosomal Protein L24 [Homo sapiens (Human)] (National Center for Biotechnology Information). https://www.ncbi.nlm.nih.gov/gene/6152.

40. Wilson-Edell, K.A., Kehasse, A., Scott, G.K., Yau, C., Rothschild, D.E., Schilling, B., Gabriel, B.S., Yevtushenko, M.A., Hanson, I.M., Held, J.M., et al. (2014). RPL24: a potential therapeutic target whose depletion or acetylation inhibits polysome assembly and cancer cell growth. Oncotarget *5*, 5165–5176. https://doi.org/10.18632/oncotarget.2099.

41. National Institutes of Health (2025). Database for Annotation, Visualization, and Integrated Discovery (DAVID). https://davidbioinformatics.nih.gov.

42. Curtis, C., Shah, S.P., Chin, S.F., Turashvili, G., Rueda, O.M., Dunning, M.J., Speed, D., Lynch, A.G., Samarajiwa, S., Yuan, Y., et al. (2012). The genomic and transcriptomic architecture of 2,000 breast tumours reveals novel subgroups. Nature *486*, 346–352. https://doi.org/10.1038/nature10983.

43. Jiang, Y.Z., Ma, D., Suo, C., Shi, J., Xue, M., Hu, X., Xiao, Y., Yu, K.D., Liu, Y.R., Yu, Y., et al. (2019). Genomic and Transcriptomic Landscape of Triple-Negative Breast Cancers: Subtypes and Treatment Strategies. Cancer Cell *35*, 428–440.e5. https://doi.org/10.1016/j.ccell.2019.02.001.

44. Yin, L., Duan, J.J., Bian, X.W., and Yu, S.C. (2020). Triple-negative breast cancer molecular subtyping and treatment progress. Breast Cancer Res. *22*, 61. https://doi.org/10.1186/s13058-020-01296-5.

45. Bar, Y., Fell, G., Dedeoglu, A., Moffett, N., Vidula, N., Spring, L., Wander, S.A., Bardia, A., Ko, N., Moy, B., et al. (2025). Dynamic HER2-low status among patients with triple negative breast cancer (TNBC) and the impact of repeat biopsies. NPJ Breast Cancer *11*, 27. https://doi.org/10.1038/s41523-025-00741-y.

46. Ma, Y., Jiao, D., Zhang, J., Lv, M., Chen, X., and Liu, Z. (2024). HER2-Low Status Was Associated With Better Breast Cancer-Specific Survival in Early-Stage Triple-Negative Breast Cancer. Oncologist *29*, E309–E318. https://doi.org/10.1093/oncolo/oyad275.

47. Wolf, D.M., Lenburg, M.E., Yau, C., Boudreau, A., and Van't Veer, L.J. (2014). Gene co-expression modules as clinically relevant hallmarks of breast cancer diversity. PLoS One *9*, e88309. https://doi.org/10.1371/journal.pone.0088309.

48. Rixe, O., Ortuzar, W., Alvarez, M., Parker, R., Reed, E., Paull, K., and Fojo, T. (1996). Oxaliplatin, Tetraplatin, Cisplatin, and Carboplatin: Spectrum of Activity in Drug-Resistant Cell Lines and in the Cell Lines of the National Cancer Institute's Anticancer Drug Screen Panel. Biochem. Pharmacol. *52*, 1855–1865. https://doi.org/10.1016/s0006-2952(97)81490-6.

49. Lokich, J., and Anderson, N. (1998). Carboplatin versus cisplatin in solid tumors: An analysis of the literature. Ann. Oncol. *9*, 13–21. https://doi.org/10.1023/A:1008215213739.

50. Nair, N.U., Greninger, P., Zhang, X., Friedman, A.A., Amzallag, A., Cortez, E., Sahu, A.D., Lee, J.S., Dastur, A., Egan, R.K., et al. (2023). A landscape of response to drug combinations in non-small cell lung cancer. Nat. Commun. *14*, 3830. https://doi.org/10.1038/s41467-023-39528-9.

51. Zhang, J., Chung, T., and Oldenburg, K. (1999). A Simple Statistical Parameter for Use in Evaluation and Validation of High Throughput Screening Assays. J. Biomol. Screen *4*, 67–73. https://doi.org/10.1177/108705719900400206.

52. Ianevski, A. (2022). SynergyFinder - User Documentation. https://synergyfinder.aittokallio.group/synfin_docs/.

53. Lang, J.E., Forero-Torres, A., Yee, D., Yau, C., Wolf, D., Park, J., Parker, B.A., Chien, A.J., Wallace, A.M., Murthy, R., et al. (2022). Safety and efficacy of HSP90 inhibitor ganetespib for neoadjuvant treatment of stage II/III breast cancer. NPJ Breast Cancer *8*, 128. https://doi.org/10.1038/s41523-022-00493-z.

54. Basu, A., Bodycombe, N.E., Cheah, J.H., Price, E.V., Liu, K., Schaefer, G.I., Ebright, R.Y., Stewart, M.L., Ito, D., Wang, S., et al. (2013). An interactive resource to identify cancer genetic and lineage dependencies targeted by small molecules. Cell *154*, 1151–1161. https://doi.org/10.1016/j.cell.2013.08.003.

55. Zou, J., Shah, O., Chiu, Y.C., Ma, T., Atkinson, J.M., Oesterreich, S., Lee, A.V., and Tseng, G.C. (2024). Systems approach for congruence and selection of cancer models towards precision medicine. PLoS Comput. Biol. *20*, e1011754. https://doi.org/10.1371/journal.pcbi.1011754.

56. Zhang, W., Wu, C., Huang, H., Bleu, P., Zambare, W., Alvarez, J., Wang, L., Paty, P.B., Romesser, P.B., Smith, J.J., and Chen, X.S. (2025). Enhancing chemotherapy response prediction via matched colorectal tumor-organoid gene expression analysis and network-based biomarker selection. Transl. Oncol. *52*, 102238. https://doi.org/10.1016/j.tranon.2024.102238.

57. Ortega-Álvarez, D., Tébar-García, D., Casado-Peláez, M., Olivares-Osuna, D., Castillo-Agea, E., Balibrea-Rull, J., Barbella-Aponte, R.A., Pérez-Parra, D., Musulén, E., Guardia, C., et al. (2026). Discovery and Evaluation of Biomarkers for Triple-Negative Breast Cancer Subtypes Uncovers Patient Stratification and Targeted Therapeutic Strategies. Cancer Res. *86*, 2360–2376. https://doi.org/10.1158/0008-5472.CAN-24-2758.

58. Cardoso, F., Paluch-Shimon, S., Senkus, E., Curigliano, G., Aapro, M.S., André, F., Barrios, C.H., Bergh, J., Bhattacharyya, G.S., Biganzoli, L., et al. (2020). 5th ESO-ESMO international consensus guidelines for advanced breast cancer (ABC 5). Ann. Oncol. *31*, 1623–1649. https://doi.org/10.1016/j.annonc.2020.09.010.

59. Koshy, N., Quispe, D., Shi, R., Mansour, R., and Burton, G.V. (2010). Cisplatin-gemcitabine therapy in metastatic breast cancer: Improved outcome in triple negative breast cancer patients compared to non-triple negative patients. Breast *19*, 246–248. https://doi.org/10.1016/j.breast.2010.02.003.

60. Zhang, J., Wang, Z., Hu, X., Wang, B., Wang, L., Yang, W., Liu, Y., Liu, G., Di, G., Hu, Z., et al. (2015). Cisplatin and gemcitabine as the first line therapy in metastatic triple negative breast cancer. Int. J. Cancer *136*, 204–211. https://doi.org/10.1002/ijc.28966.

61. Pandy, J.G.P., Balolong-Garcia, J.C., Cruz-Ordinario, M.V.B., and Que, F.V.F. (2019). Triple negative breast cancer and platinum-based systemic treatment: a meta-analysis and systematic review. BMC Cancer *19*, 1065. https://doi.org/10.1186/s12885-019-6253-5.

62. Mayer, E.L., Abramson, V., Jankowitz, R., Falkson, C., Marcom, P.K., Traina, T., Carey, L., Rimawi, M., Specht, J., Miller, K., et al. (2020). TBCRC 030: a phase II study of preoperative cisplatin versus paclitaxel in triple-negative breast cancer: evaluating the homologous recombination deficiency (HRD) biomarker. Ann. Oncol. *31*, 1518–1525. https://doi.org/10.1016/j.annonc.2020.08.2064.

63. Park, J.H., Ahn, J.H., and Kim, S.B. (2018). How shall we treat early triple-negative breast cancer (TNBC): from the current standard to upcoming immuno-molecular strategies. ESMO Open *3*, e000357. https://doi.org/10.1136/ESMOOPEN-2018-000357.

64. ClinicalTrials.gov (2024). Combination of Olaparib and Navitoclax in Women with HGSC and TNBC (National Library of Medicine). https://clinicaltrials.gov/study/NCT05358639.

65. Muttiah, C., Whittle, J.R., Oakman, C., and Lindeman, G.J. (2022). PALVEN: Phase Ib trial of palbociclib, letrozole and venetoclax in estrogen receptor-and BCL2-positive advanced breast cancer. Future Oncol. *18*, 1805–1816. https://doi.org/10.2217/fon-2021-1450.

66. Xiang, L., Gilkes, D.M., Chaturvedi, P., Luo, W., Hu, H., Takano, N., Liang, H., and Semenza, G.L. (2014). Ganetespib blocks HIF-1 activity and inhibits tumor growth, vascularization, stem cell maintenance, invasion, and metastasis in orthotopic mouse models of triple-negative breast cancer. J. Mol. Med. *92*, 151–164. https://doi.org/10.1007/s00109-013-1102-5.

67. Proia, D.A., Zhang, C., Sequeira, M., Jimenez, J.P., He, S., Spector, N., Shapiro, G.I., Tolaney, S., Nagai, M., Acquaviva, J., et al. (2014). Preclinical activity profile and therapeutic efficacy of the hsp90 inhibitor ganetespib in triple-negative breast cancer. Clin. Cancer Res. *20*, 413–424. https://doi.org/10.1158/1078-0432.CCR-13-2166.

68. Mccready, J., Wong, D.S., Burlison, J.A., Ying, W., and Jay, D.G. (2014). An impermeant ganetespib analog inhibits extracellular Hsp90-mediated cancer cell migration that involves Lysyl oxidase 2-like protein. Cancers (Basel) *6*, 1031–1046. https://doi.org/10.3390/cancers6021031.

69. Porter, R.J., Murray, G.I., and McLean, M.H. (2020). Current concepts in tumour-derived organoids. Br. J. Cancer *123*, 1209–1218. https://doi.org/10.1038/s41416-020-0993-5.

70. Vanmeerbeek, I., Naulaerts, S., and Garg, A.D. (2023). Reverse translation: the key to increasing the clinical success of immunotherapy? Genes Immun. *24*, 217–219. https://doi.org/10.1038/s41435.

71. Cattaneo, C.M., Dijkstra, K.K., Fanchi, L.F., Kelderman, S., Kaing, S., van Rooij, N., van den Brink, S., Schumacher, T.N., and Voest, E.E. (2020). Tumor organoid-T-cell coculture systems. Nat. Protoc. *15*, 15–39. https://doi.org/10.1038/s41596-019-0232-9.

72. Fujii, M., Matano, M., Nanki, K., and Sato, T. (2015). Efficient genetic engineering of human intestinal organoids using electroporation. Nat. Protoc. *10*, 1474–1485. https://doi.org/10.1038/nprot.2015.088.

73. Cancer Genome Atlas Research Network; Weinstein, J.N., Collisson, E.A., Mills, G.B., Shaw, K.R.M., Ozenberger, B.A., Ellrott, K., Shmulevich, I., Sander, C., and Stuart, J.M. (2013). The Cancer Genome Atlas Pan-Cancer analysis project. Nat. Genet. *45*, 1113–1120. https://doi.org/10.1038/ng.2764.

74. Mudge, J.M., Carbonell-Sala, S., Diekhans, M., Martinez, J.G., Hunt, T., Jungreis, I., Loveland, J.E., Arnan, C., Barnes, I., Bennett, R., et al. (2025). GENCODE 2025: reference gene annotation for human and mouse. Nucleic Acids Res. *53*, D966–D975. https://doi.org/10.1093/nar/gkae1078.

75. Durinck, S., Moreau, Y., Kasprzyk, A., Davis, S., De Moor, B., Brazma, A., and Huber, W. (2005). BioMart and Bioconductor: a powerful link between biological databases and microarray data analysis. Bioinformatics *21*, 3439–3440. https://doi.org/10.1093/bioinformatics/bti525.

76. Langmead, B., and Salzberg, S.L. (2012). Fast gapped-read alignment with Bowtie 2. Nat. Methods *9*, 357–359. https://doi.org/10.1038/nmeth.1923.

77. Boeva, V., Popova, T., Bleakley, K., Chiche, P., Cappo, J., Schleiermacher, G., Janoueix-Lerosey, I., Delattre, O., and Barillot, E. (2012). Control-FREEC: a tool for assessing copy number and allelic content using next-generation sequencing data. Bioinformatics *28*, 423–425. https://doi.org/10.1093/bioinformatics/btr670.

78. Love, M.I., Huber, W., and Anders, S. (2014). Moderated estimation of fold change and dispersion for RNA-seq data with DESeq2. Genome Biol. *15*, 550. https://doi.org/10.1186/s13059-014-0550-8.

79. DePristo, M.A., Banks, E., Poplin, R., Garimella, K.V., Maguire, J.R., Hartl, C., Philippakis, A.A., del Angel, G., Rivas, M.A., Hanna, M., et al. (2011). A framework for variation discovery and genotyping using next-generation DNA sequencing data. Nat. Genet. *43*, 491–498. https://doi.org/10.1038/ng.806.

80. Gendoo, D.M.A., Ratanasirigulchai, N., Schröder, M.S., Paré, L., Parker, J.S., Prat, A., and Haibe-Kains, B. (2016). Genefu: an R/Bioconductor package for computation of gene expression-based signatures in breast cancer. Bioinformatics *32*, 1097–1099. https://doi.org/10.1093/bioinformatics/btv693.

81. Skidmore, Z.L., Wagner, A.H., Lesurf, R., Campbell, K.M., Kunisaki, J., Griffith, O.L., and Griffith, M. (2016). GenVisR: Genomic Visualizations in R. Bioinformatics *32*, 3012–3014. https://doi.org/10.1093/bioinformatics/btw325.

82. Schloerke, B., Cook, D., Larmarange, J., Briatte, F., Marbach, M., Thoen, E., Elberg, A., and Crowley, J. (2024). GGally: Extension to "ggplot2". v2.2.1. https://doi.org/10.32614/CRAN.package.GGally.

83. Horikoshi, M., and Tang, Y. (2015). ggfortify: Data Visualization Tools for Statistical Analysis Results. https://doi.org/10.32614/CRAN.package.ggfortify.

84. Wickham, H., Chang, W., Henry, L., Pedersen, T.L., Takahashi, K., Wilke, C., Woo, K., Yutani, H., Dunnington, D., and van den Brand, T. (2007). ggplot2: Create Elegant Data Visualisations Using the Grammar of Graphics. https://doi.org/10.32614/CRAN.package.ggplot2.

85. Hafner, M., Niepel, M., Chung, M., and Sorger, P.K. (2016). Growth rate inhibition metrics correct for confounders in measuring sensitivity to cancer drugs. Nat. Methods *13*, 521–527. https://doi.org/10.1038/nmeth.3853.

86. Hänzelmann, S., Castelo, R., and Guinney, J. (2013). GSVA: gene set variation analysis for microarray and RNA-seq data. BMC Bioinf. *14*, 7. https://doi.org/10.1186/1471-2105-14-7.

87. Mayakonda, A., Lin, D.-C., Assenov, Y., Plass, C., and Koeffler, H.P. (2018). Maftools: efficient and comprehensive analysis of somatic variants in cancer. Genome Res. *28*, 1747–1756. https://doi.org/10.1101/gr.239244.118.

88. Dolgalev, I. (2018). msigdbr: MSigDB Gene Sets for Multiple Organisms in a Tidy Data Format. https://doi.org/10.32614/CRAN.package.msigdbr.

89. Kolde, R. (2019). pheatmap: Pretty Heatmaps. v1.0.12. https://doi.org/10.32614/CRAN.package.pheatmap.

90. Bolstad, B. (2026). preprocessCore: A collection of pre-processing functions. https://doi.org/10.18129/B9.bioc.preprocessCore.

91. Robin, X., Turck, N., Hainard, A., Tiberti, N., Lisacek, F., Sanchez, J.-C., and Müller, M. (2011). pROC: an open-source package for R and S+ to analyze and compare ROC curves. BMC Bioinf. *12*, 77. https://doi.org/10.1186/1471-2105-12-77.

92. Li, B., and Dewey, C.N. (2011). RSEM: accurate transcript quantification from RNA-Seq data with or without a reference genome. BMC Bioinf. *12*, 323. https://doi.org/10.1186/1471-2105-12-323.

93. Ianevski, A., He, L., Aittokallio, T., and Tang, J. (2017). SynergyFinder: a web application for analyzing drug combination dose-response matrix data. Bioinformatics *33*, 2413–2415. https://doi.org/10.1093/bioinformatics/btx162.

94. Dekkers, J.F., van Vliet, E.J., Sachs, N., Rosenbluth, J.M., Kopper, O., Rebel, H.G., Wehrens, E.J., Piani, C., Visvader, J.E., Verissimo, C.S., et al. (2021). Long-term culture, genetic manipulation and xenotransplantation of human normal and breast cancer organoids. Nat. Protoc. *16*, 1936–1965. https://doi.org/10.1038/s41596-020-00474-1.

95. Rosenbluth, J.M., Schackmann, R.C.J., Gray, G.K., Selfors, L.M., Li, C.M.C., Boedicker, M., Kuiken, H.J., Richardson, A., Brock, J., Garber, J., et al. (2020). Organoid cultures from normal and cancer-prone human breast tissues preserve complex epithelial lineages. Nat. Commun. *11*, 1711. https://doi.org/10.1038/s41467-020-15548-7.

96. Tempus AI Inc (2026). Tempus xT Validation Summary (Tempus AI, Inc. Official Website). https://www.tempus.com/document-library/tempus-xt-validation/.

97. Beaubier, N., Bontrager, M., Huether, R., Igartua, C., Lau, D., Tell, R., Bobe, A.M., Bush, S., Chang, A.L., Hoskinson, D.C., et al. (2019). Integrated genomic profiling expands clinical options for patients with cancer. Nat. Biotechnol. *37*, 1351–1360. https://doi.org/10.1038/s41587-019-0259-z.

98. Beaubier, N., Tell, R., Lau, D., Parsons, J.R., Bush, S., Perera, J., Sorrells, S., Baker, T., Chang, A., Michuda, J., et al. (2019). Clinical validation of the tempus xT next-generation targeted oncology sequencing assay. Oncotarget *10*, 2384–2396. https://doi.org/10.18632/oncotarget.26797.

99. Wang, K., Li, M., and Hakonarson, H. (2010). ANNOVAR: Functional annotation of genetic variants from high-throughput sequencing data. Nucleic Acids Res. *38*, e164. https://doi.org/10.1093/nar/gkq603.

100. UCSF Health Center for Clinical Genetics and Genomics (2025). UCSF 500 Cancer Gene Panel Test (UCSF500/UC500). https://genomics.ucsf.edu/UCSF500.

101. COSMIC (Sanger Institute) (2025). Data Downloads (Release V102. https://cancer.sanger.ac.uk/cosmic/download/cosmic.

102. National Cancer Institute (2025). Genomic Data Commons (TCGA - PanCanAtlas Publications). https://gdc.cancer.gov/about-data/publications/pancanatlas.

103. Burstein, M.D., Tsimelzon, A., Poage, G.M., Covington, K.R., Contreras, A., Fuqua, S.A.W., Savage, M.I., Osborne, C.K., Hilsenbeck, S.G., Chang, J.C., et al. (2015). Comprehensive genomic analysis identifies novel subtypes and targets of triple-negative breast cancer. Clin. Cancer Res. *21*, 1688–1698. https://doi.org/10.1158/1078-0432.CCR-14-0432.

104. Lehmann, B.D., Jovanović, B., Chen, X., Estrada, M.V., Johnson, K.N., Shyr, Y., Moses, H.L., Sanders, M.E., and Pietenpol, J.A. (2016). Refinement of triple-negative breast cancer molecular subtypes: Implications for neoadjuvant chemotherapy selection. PLoS One *11*, e0157368. https://doi.org/10.1371/journal.pone.0157368.

105. Lehmann, B.D., Bauer, J.A., Chen, X., Sanders, M.E., Chakravarthy, A.B., Shyr, Y., and Pietenpol, J.A. (2011). Identification of human triple-negative breast cancer subtypes and preclinical models for selection of targeted therapies. J. Clin. Investig. *121*, 2750–2767. https://doi.org/10.1172/JCI45014.

106. Liberzon, A., Birger, C., Thorvaldsdóttir, H., Ghandi, M., Mesirov, J.P., and Tamayo, P. (2015). The Molecular Signatures Database (MSigDB) hallmark gene set collection. Cell Syst. *1*, 417–425. https://doi.org/10.1016/j.cels.2015.12.004.

107. Bergamaschi, A., Tagliabue, E., Sørlie, T., Naume, B., Triulzi, T., Orlandi, R., Russnes, H.G., Nesland, J.M., Tammi, R., Auvinen, P., et al. (2008). Extracellular matrix signature identifies breast cancer subgroups with different clinical outcome. J. Pathol. *214*, 357–367. https://doi.org/10.1002/path.2278.

108. Amara, D., Wolf, D.M., van 't Veer, L., Esserman, L., Campbell, M., and Yau, C. (2017). Co-expression modules identified from published immune signatures reveal five distinct immune subtypes in breast cancer. Breast Cancer Res. Treat. *161*, 41–50. https://doi.org/10.1007/s10549-016-4041-3.

109. Teschendorff, A.E., Gomez, S., Arenas, A., El-Ashry, D., Schmidt, M., Gehrmann, M., and Caldas, C. (2010). Improved prognostic classification of breast cancer defined by antagonistic activation patterns of immune response pathway modules. BMC Cancer *10*, 604. https://doi.org/10.1186/1471-2407-10-604.

110. Rody, A., Karn, T., Liedtke, C., Pusztai, L., Ruckhaeberle, E., Hanker, L., Gaetje, R., Solbach, C., Ahr, A., Metzler, D., et al. (2011). A clinically relevant gene signature in triple negative and basal-like breast cancer. Breast Cancer Res. *13*, R97. https://doi.org/10.1186/bcr3035.

111. Palmer, C., Diehn, M., Alizadeh, A.A., and Brown, P.O. (2006). Cell-type specific gene expression profiles of leukocytes in human peripheral blood. BMC Genom. *7*, 115. https://doi.org/10.1186/1471-2164-7-115.

112. Danaher, P., Warren, S., Dennis, L., D'Amico, L., White, A., Disis, M.L., Geller, M.A., Odunsi, K., Beechem, J., and Fling, S.P. (2017). Gene expression markers of Tumor Infiltrating Leukocytes. J. Immunother. Cancer *5*, 18. https://doi.org/10.1186/s40425-017-0215-8.

113. Bianchini, G., Pusztai, L., Karn, T., Iwamoto, T., Rody, A., Kelly, C., Müller, V., Schmidt, S., Qi, Y., Holtrich, U., et al. (2013). Proliferation and estrogen signaling can distinguish patients at risk for early versus late relapse among estrogen receptor positive breast cancers. Breast Cancer Res. *15*, R86. https://doi.org/10.1186/bcr3481.

114. Rody, A., Holtrich, U., Pusztai, L., Liedtke, C., Gaetje, R., Ruckhaeberle, E., Solbach, C., Hanker, L., Ahr, A., Metzler, D., et al. (2009). T-cell metagene predicts a favorable prognosis in estrogen receptor-negative and HER2-positive breast cancers. Breast Cancer Res. *11*, R15. https://doi.org/10.1186/bcr2234.

STAR★METHODS

KEY RESOURCES TABLE

REAGENT or RESOURCE	SOURCE	IDENTIFIER
Antibodies		
Mouse monoclonal anti-Ki-67 B56	Abcam	Cat#ab279653; RRID: AB_2934265
Anti-mouse IgG, HRP-linked antibody	CST	Cat#7076; RRID: AB_330924
Chemicals, peptides, and recombinant proteins		
A83-01	SigmaAldrich	Cat#SML0788-5MG
ABT-263	SelleckChem	Cat#S1001
Advanced DMEM/F-12 (Dulbecco's Modified Eagle Medium/Ham's F-12)	ThermoFisher	Cat#12634-028
AT9283	SigmaAldrich	Cat#SML2394-5MG
B27 Supplement	ThermoFisher	Cat#17504-044
Basement Membrane Extract Type 2	BioTechne	Cat#3533-005-02
β-Estradiol	SigmaAldrich	Cat#E2257-1MG
BV6	SigmaAldrich	Cat#SIAL-5339650001
Cisplatin	SigmaAldrich	Cat#232120-50MG
Clofarabine	Thomas Scientific (Cayman Chemical)	Cat#14125-10
Collagenase	SigmaAldrich	Cat#C9407-1G
Dulbecco's Modified Eagle Medium	ThermoFisher	Cat#11995-073
Eosin Y	SigmaAldrich	Cat#SIAL-1170811000
Fetal Bovine Serum	Cytiva	Cat#SH30910.02
Forskolin	SigmaAldrich	Cat#F6886-10MG
Ganetespib	Medchemexpress	Cat#HY-15205-10MG
Geneticin	ThermoFisher	Cat#10131-035
Glutamax	ThermoFisher	Cat#35050-061
Hematoxylin	Sigma Aldrich	Cat#MHS32-1L
HEPES	ThermoFisher	Cat#15630-080
Histogel	Epredia	Cat#HG-4000-012
Human EGF	Peprotech	Cat#AF-100-15-100UG
Human FGF-7	Peprotech	Cat#100-19-500UG
Human FGF-10	Peprotech	Cat#100-26-500UG
Human Heregulin β1	Peprotech	Cat#100-03-50UG
Noggin-conditioned medium (from Noggin producing cell line)	H. Clevers laboratory[71]	31853056; https://doi.org/10.1038/s41596-019-0232-9
R-spondin1–conditioned medium (from R-spondin1–producing cell line)	C. Kuo laboratory[72]	26334867; https://doi.org/10.1038/nprot.2015.088
Wnt3A-conditioned medium (from L-Wnt3A cell line)	H. Clevers laboratory[72]	26334867; https://doi.org/10.1038/nprot.2015.088
Hydrocortisone	SigmaAldrich	Cat#H0888-10G
N-Acetylcysteine	SigmaAldrich	Cat#A0737-25MG
Nicotinamide	SigmaAldrich	Cat#N0636-100G
JNJ26481585	SelleckChem	Cat#S1096
Obatoclax mesylate	SelleckChem	Cat#S1057
Penicillin-Streptomycin	ThermoFisher	Cat#15140-122
Primocin	InvivoGen	Cat#Ant-pm-05
Recovery Cell Culture Freezing Medium	ThermoFisher	Cat#12648-010
SB202190	SigmaAldrich	Cat#S7067-25MG
Trichostatin A	SelleckChem	Cat#S1045

(*Continued on next page*)

Continued

REAGENT or RESOURCE	SOURCE	IDENTIFIER
TRIzol	ThermoFisher	Cat#15596018
Trypsin	Corning	Cat#25-053-C1
TryplE	ThermoFisher	Cat#12604-013
Veliparib	SelleckChem	Cat#S1004
Vorinostat	Fisher	Cat#50-202-8703
Y27632	Abcam	Cat#ab143784
Critical commercial assays		
Cell Titer Glo	Promega	Cat#G7571
CellTox^{TM} Green Cytotoxicity Assay	Promega	Cat#G8743
DNeasy Blood & Tissue Kit®	Qiagen	Cat#69504
Purelink RNA Mini Kit® Protocol	ThermoFisher	Cat#12183020
Tempus xT assay	Tempus AI, Inc., Chicago, IL	N/A
Deposited data		
HUB Living Biobank gene expression matrix	Sachs et al.[3]	29224780; https://doi.org/10.1016/j.cell.2017.11.010
I-SPY2-990 mRNA/RPPA Data Resource: mRNA component	Wolf et al.[5]	35623341; https://doi.org/10.1016/j.ccell.2022.05.005; NCBI GEO accession number GSE194040
TCGA PanCanAtlas RNA (Final)	Cancer Genome Atlas Research Network et al.[73]	24071849; https://doi.org/10.1038/ng.2764
TNBC TORG raw and analyzed bulk RNA-Seq data	This paper	NCBI GEO accession number GSE303509
The Human GENCODE Gene Set, Release 49 (09.2025), Genome assembly version GRCh38.p14, Ensembl release 115	Mudge et al.[74]	39565199; https://doi.org/10.1093/nar/gkae1078
Experimental models: Cell lines		
TNBC TORG organoid lines	This study	Table S3
Software and algorithms		
R	R project for statistical computing	https://www.r-project.org
biomaRt v2.60.1	Durinck et al.[75]	16082012; https://doi.org/10.1093/bioinformatics/bti525
Bowtie2 v2.5.5	Langmead et al.[76]	22388286; https://doi.org/10.1038/nmeth.1923
Control-FREEC	Boeva et al.[77]	22155870; https://doi.org/10.1093/bioinformatics/btr670
DESeq2 v1.44.0	Love et al.[78]	25516281; https://doi.org/10.1186/s13059-014-0550-8
GATK	DePristo et al.[79]	21478889; https://doi.org/10.1038/ng.806
genefu v2.36.0	Gendoo et al.[80]	26607490; https://doi.org/10.1093/bioinformatics/btv693
GenVisR v1.36.0	Skidmore et al.[81]	27288499; https://doi.org/10.1093/bioinformatics/btw325
GGally v2.2.1	Schloerke et al.[82]	https://doi.org/10.32614/CRAN.package.Ggally
ggfortify v0.4.17	Horikoshi et al.[83]	https://doi.org/10.32614/CRAN.package.ggfortify
ggplot2 v3.5.2	Wickham et al.[84]	https://doi.org/10.32614/CRAN.package.ggplot2
GRmetrics v1.30.0	Hafner et al.[85]	27135972; https://doi.org/10.1038/nmeth.3853

(*Continued on next page*)

Continued

REAGENT or RESOURCE	SOURCE	IDENTIFIER
GSVA v1.52.3	Hänzelmann et al.[86]	23323831; https://doi.org/10.1186/1471-2105-14-7
Human MSigDB v2024.1.Hs GMTs	Liberzon et al.[26]	26771021; https://doi.org/10.1016/j.cels.2015.12.004
maftools v2.20.0	Mayakonda et al.[87]	30341162; https://doi.org/10.1101/gr.239244.118
msigdbr v26.1.0	Dolgalev[88]	https://doi.org/10.32614/CRAN.package.msigdbr
pheatmap v1.0.12	Kolde[89]	https://doi.org/10.32614/CRAN.package.pheatmap
preprocessCore v1.66.0	Bolstad[90]	https://doi.org/10.18129/B9.bioc.preprocessCore
pROC v1.18.5	Robin et al.[91]	21414208; https://doi.org/10.1186/1471-2105-12-77
RSEM v1.3.1	Li et al.[92]	21816040; https://doi.org/10.1186/1471-2105-12-323
synergyfinder v3.12.0	Ianevski et al.[93]	28379339; https://doi.org/10.1093/bioinformatics/btx162
Illustrator 2025	Adobe	www.adobe.com
Photoshop 2025	Adobe	www.adobe.com
BioRender: Scientific Image and Illustration Software	BioRender	www.biorender.com
Prism v10.5.0 (673)	GraphPad	www.graphpad.com
Incucyte S3-C2	Sartorius	www.sartorius.com

EXPERIMENTAL MODEL AND STUDY PARTICIPANT DETAILS

HUB Living Biobank data

Analyzed, anonymized RNA-seq counts data of 26 breast cancer organoids that were published by Sachs et al. (2018) were received from the authors of the original publication.[3] All other (clinical) sample information related to the HUB Living Biobank was retrieved from the supplements to the original publication.

I-SPY2 trial data

The ongoing I-SPY2, multi-center phase 2 neoadjuvant chemo-/targeted-therapy platform trial was designed to rapidly screen promising experimental agents and identify improved treatment regimens in women with early-stage high molecular risk breast cancer, as well as to identify and propose potential biomarkers of response and resistance. The trial enrolls patients with stage II-III breast cancer based on hormone receptors (HR), HER2, and MammaPrint status, who meet tumor size criteria. Patients are randomized across multiple experimental arms, testing investigational agents in combination with standard chemotherapy, or the standard chemotherapy control arm. The primary endpoint of the trial is pathologic complete response (pCR), defined as the absence of invasive breast cancer in the breast and regional nodes at the time of surgery due to the complete eradication of the cancer cells by the preceding therapy.

For the biomarker analysis and predictive modeling in this manuscript, published biomarker expression and clinical data from 986 patients in the I-SPY2 trial[5] across 10 treatment arms were downloaded from the Gene Expression Omnibus (GEO) repository GSE194040 (URL: https://www.ncbi.nlm.nih.gov/geo/query/acc.cgi?acc=GSE194040).

TNBC TORG biobank

Between 2018 and 2026, breast tumor tissues were obtained from patients with early-stage breast cancer during lumpectomy, mastectomy, or core biopsy procedures under Protocol 10–458 at Dana-Farber Cancer Institute/Brigham & Women's Hospital and under IRB Protocol 23–39361 at the University of California, San Francisco. Patients gave their informed consent to have tissue used for scientific research purposes. Anonymized clinical information for all 14 organoid samples is available in Table S3. Because male breast cancer is rare, all samples in this study were collected from female breast cancer patients, and the influence of sex on the results of this study could not be determined.

Tumor organoids were grown in three-dimensional cultures following a published protocol.[94] In short, tissue samples were processed on the day of surgery. Upon arrival in the lab, tissues were photographed for documentation and, if big enough, divided into two or more 10–15 mm^3 pieces. Of each tissue piece, if big enough, a small section was cut off and formalin-fixed and paraffin-embedded (FFPE) for histopathological analysis. In the case of multiple tissue pieces, one or more pieces of the fresh tissue were viably frozen in 90% Fetal Bovine Serum (FBS; Cytiva, Cat. No. SH30910.02) and 10% DMSO and banked in liquid nitrogen for the subsequent generation of organoid cultures. The remaining tissue piece was minced and either directly embedded in a 50 μL drop of Cultrex basement membrane extract (BME) type 2 (BioTechne, Cat. No. 3532-005-02) in the center of a well in a pre-warmed 24-well tissue culture plate (Greiner, Cat. No. M9312 and CytoOne, Cat. No. CC7672-7524) or first digested by placing the minced tissue into a 50 mL conical tube containing 1 mg/mL collagenase (Sigma, Cat. No. C9407) and 20 mL AdDF+++ (Advanced DMEM/F12 (Thermo Fisher, Cat. No. 12634-028) containing 1× Glutamax (Thermo Fisher, Cat. No. 35050-061), 10mM HEPES (Thermo Fisher, Cat. No. 15630-080), and antibiotics: penicillin-streptomycin (Thermo Fisher, Cat. No. 15140-122) and Primocin (Invivogen, Cat. No. Ant-pm-05)). Tubes containing minced tissue and collagenase were wrapped in parafilm and placed on their side in an orbital shaker at 37°C for 15 to 30 min. After digestion, 30 mL AdDF+++ and 2% FBS were added, and the organoids were pelleted. Further mechanical shearing was achieved by adding 10 mL AdDF+++ and sequentially pipetting with 10-, 5-, and 1-mL pipette tips before the final pellet of primary breast organoids was obtained and embedded in a 50 μL drop of BME. BME drops were allowed to harden for 20 min at 37°C, after which 500 μL of a fully defined type 1 or type 2 organoid culture medium[94] was placed over the droplet. Type 1 medium is made of AdDF+++, R-spondin-1-conditioned medium,[72] Noggin-conditioned medium,[71] B27 supplement, nicotinamide, N-acetyl-L-cysteine, Y-27632, Heregulin β1, human FGF-7, human FGF-10, A83–01, human EGF and SB202190. Type 2 medium consists of AdDF+++, Wnt3a-conditioned medium,[72] R-spondin-1-conditioned medium, Noggin-conditioned medium, B27 supplement, nicotinamide, N-acetyl-L-cysteine, hydrocortisone, β-estradiol, Forskolin, Y-27632, Heregulin β1, human FGF-10, A83–01, and human EGF. Organoids were stably cultured in the default organoid medium (type 1, 71%), and in type 2 medium (29%) if stable growth was not achieved in type 1. Plates were kept in humidified 37°C/5% CO_2 incubators at ambient O_2, and the organoid medium was changed every 2–3 days. Organoids were passaged at different rates depending on the growth rate of the organoids, varying from once a week, to once in 4 or more weeks. Most organoids had a solid morphology and a moderate growth rate. 0.5 mL of TrypLE (Gibco, Cat. No. 12604013) per 2–3 wells of organoids was used to break down the organoids into smaller clumps of cells, following the addition of 10 mL AdDF+++, 0.2 mL FBS to deactivate the TrypLE and centrifugation at 300*g* for 3 min. Organoids were re-plated in BME domes at ratios varying from 1:1–1:6 depending on individual organoid growth characteristics. Growth rate was categorized as "Fast" (split at least 1:2 every week), "Moderate" (split 1:2 every 1–4 weeks), or "Slow" (split 1:2 every 4 or more weeks). Established cultures with organoids were viably frozen on a regular basis and banked at different passage numbers using Recovery Cell Culture Freezing Medium (Life Technologies, Cat. No. 12648-010) for future use.

Organoids were maintained under consistent culture conditions and routinely tested for mycoplasma contamination. The majority (excluding two organoids) underwent at least one freeze-thaw cycle. Depending on growth rate and success, organoids were submitted for bulk RNA sequencing and, if possible, whole-genome sequencing, in that order, after they had reached at least 4 passages, except for one culture (TORG565), which was passaged twice.

METHOD DETAILS

Predictive models of treatment response derived from the I-SPY2 trial biomarker resource

Published, matched biomarker and clinical outcomes data of patients of the first ten treatment arms (one control arm with standard chemotherapy and nine experimental arms with agents targeting a wide variety of pathways, including PARP inhibitors, AKT inhibitors, DNA-damaging agents, angiogenesis inhibitors, immunotherapy, small-molecule pan-HER2 inhibitors, and dual-HER2-targeting agents) of the I-SPY2 trial (n = 987 samples from n = 986 patients; the "I-SPY2-990 dataset") were used here to develop a mathematical prediction model for treatment response, based on the expression of biomarkers derived from patient tissue molecular data.[5]

Wolf et al.[5] originally reported twenty-three gene expression signatures that were successful qualifying biomarkers in I-SPY2 (reflecting DNA repair deficiency (n = 2), immune activation (n = 8), ER signaling (n = 2), HER2 signaling (n = 2), proliferation (n = 3), (phospho)activation of AKT and mTOR (n = 2), and ANG/TIE2 (n = 1) pathways, among others). These biomarkers were classified as 'qualifying biomarkers' in I-SPY2, meaning that they were pre-specified for analysis based on existing evidence that suggested a role in treatment response prediction, and they were tested prospectively for their specific response-predictive value using a pre-specified statistical framework. Many had a significant association with response (pathologic complete response; pCR) or non-response (non-pCR) to one or more treatments in the trial.

Of the 23 qualifying gene expression biomarkers from I-SPY2, we included in our analysis only those biomarkers that were detectable in organoid cultures (as defined by being variably expressed at levels greater than the 10th percentile in at least 10% of samples in the HUB biobank of breast cancer organoids, and excluded proprietary biomarkers (e.g., BluePrint/MammaPrint[29,30]), resulting in a final list of 9 qualifying gene expression markers from Wolf et al.[5] that could be assessed in organoids.

Using the I-SPY2-990 patient outcomes data (pathologic complete response; pCR) and the I-SPY2-990 patient tumor tissue expression level of these 9 organoid-evaluable biomarkers, we performed a multivariable logistic regression analysis across all receptor subgroup-treatment arm combinations. We used the step() function in R to perform forward selection and selected the

resulting four models with three or more predictors. We then manually reviewed these models using summary(), and in case of any non-significant terms, we subsequently used drop1(model, test = "Chisq") to remove variables that did not significantly contribute to the model (significant when $p < 0.05$). Of these revised models, only three had more than one significant predictor. Receiver operating characteristic (ROC) plots were made using pROC in R.[91]

We selected the model that predicted response to VC in patients with TNBC for further validation. Applying the predictive model of response to VC in TNBC organoids of the HUB and TNBC TORG biobanks, as well as patient tissues from the TCGA and I-SPY datasets, yielded a ranking of samples from most predicted sensitive to most predicted resistant.

Organoid embedding and staining (hematoxylin & eosin staining, immunohistochemistry)

After embedding intact BME gels containing organoids in histogel (Epredia, Cat. No. HG-4000-012) and fixing them in formalin,[95] they were paraffin-embedded, sectioned, and prepared for Hematoxylin & Eosin (H&E) staining and immunohistochemistry.

For H&E staining, slides were deparaffinized and rehydrated, then stained in hematoxylin (Sigma Aldrich, Cat. No. MHS32-1L) for 3 min, followed by washing in water until the water ran clear. They were destained in 100% ethanol for 1 min and subsequently stained in eosin Y (Sigma Aldrich, Cat. No. SIAL-1170811000) for 10 min, followed by washing in 100% ethanol twice for 5 min each. After dehydration, the slides were covered with coverslips. Immunohistochemistry was performed as published, using standard antigen retrieval, blocking, and antibody staining protocols, followed by brightfield imaging.[95]

Organoid DNA and RNA extraction

Organoid DNA Extraction

Organoids passage 4 or higher, except TORG565 as described above, were harvested when confluent by digesting them using TrypLE, followed by a wash in AdDF+++ and FBS, after which the pellet was resuspended in 500 μL PBS, pelleted again, and then snap-frozen in dry ice-cooled ethanol (Thomas, Cat. No C961Y21). Organoids were stored at −80°C until DNA was extracted using DNeasy Blood & Tissue Kit (Qiagen, Cat. No. 69504). Samples were eluted in 30 μL AE buffer. For lower-confluency samples where a low DNA yield was expected, the 30 μL eluate was run through the spin cartridge a second time to maximize the elution. Sample concentration was measured by Qubit analysis of 1 μL of sample. Samples were stored at −20°C until submission for library preparation and sequencing.

Organoid RNA Extraction

Organoids passage 4 or higher, except TORG565 as described above, were harvested when confluent using TRIzol reagent (ThermoFisher, Cat. No. 15596018). Chloroform was added (Sigma Aldrich, Cat. No. C2432-1L) according to the Purelink RNA Mini Kit Protocol (ThermoFisher, cat# 12183020). Samples were eluted in 30 μL RNase-free water through two serial elutions. Sample concentration was measured using a NanoDrop spectrophotometer. Samples were stored at −80°C until submission for library preparation and sequencing.

DNA sequencing and analysis

For organoids with corresponding primary tumor tissue available ($n = 7$), next-generation sequencing was conducted using the Tempus xT assay (Tempus AI, Inc., Chicago, IL),[96–98] on both the primary tumor (FFPE) and the derived organoid cultures to identify cancer-associated mutations and confirm concordance of the mutational profiles. Briefly, Tempus xT is a targeted, tumor-normal-matched DNA panel that detects single-nucleotide variants (SNVs), insertions and/or deletions (indels), and copy number variants in 648 cancer-associated genes, as well as chromosomal rearrangements in 22 genes with high sensitivity and specificity. Variant calling and quality control were performed by the sequencing provider as part of the standard Tempus xT.v4 clinical workflow using clinically validated bioinformatics pipelines. When matched normal tissue was unavailable, the Tempus AI tumor-only pipeline was applied, using a computational germline variant filtering approach that incorporates population reference databases to prioritize likely somatic variants.

When insufficient primary tumor tissue was available, whole-genome sequencing (WGS) was performed on the organoid culture alone. Three organoids were run through both pipelines (targeted DNA sequencing and WGS) for technical cross-validation. Whole-genome sequencing and analysis of TNBC TORG breast cancer organoids were performed at Novogene. In short, genomic DNA libraries were constructed and, after quality control, sequenced on the Illumina NovaSeq X Plus platform (PE150). Raw data were transformed into short-read sequences and recorded in FASTQ format. Quality control procedures filtered reads containing adapters or with low sequencing quality. High-quality reads were aligned to the reference genome to generate BAM files for subsequent analyses. Matched normal samples were not available for these cases; therefore, germline variants could not be definitively excluded. CNVs were detected using Control-FREEC.[77] SNPs and InDels were identified using GATK[79] HaplotypeCaller, with variant filtering performed using the GATK VariantFiltration module, followed by annotation using ANNOVAR. SNPs/InDels that were annotated with a COSMIC ID by ANNOVAR were filtered for breast cancer-associated disease annotations.[99] Then, we selected only genes present in the UCSF500 list,[100] and lastly, we removed all variations that did not have a "GENOMIC_MUTATION_ID" in COSMIC v102 (CancerMutationCensus_AllData_Tsv_v102_GRCh37.tar).[101] Finally, we excluded all genes for which we found multiple hits in at least half of the biobank as probable technical artifacts.

SNPs, InDels, and CNV calls generated using the two pipelines were converted to a common format and harmonized to visualize broad patterns across the biobank. This yielded a list of 1079 total SNPs and InDels across the full TORG dataset ($n = 10$). The plot

showing mutation frequencies was generated using maftools[87] in R. For samples that underwent both pipelines, WGS data were prioritized over targeted sequencing data for CNV calls. CNV visualization was performed using GenVisR.[81]

Breast cancer organoid validation and quality control

Because normal breast epithelial cells can proliferate under the culture conditions used, all organoid cultures underwent breast pathology review and molecular validation prior to inclusion in downstream analyses to confirm organoid identity and tumor content. Organoids were excluded if pathology review demonstrated substantial contamination by normal breast tissue and insufficient material was available for molecular characterization. In addition, for organoids with matched primary tumor tissue available, organoids were excluded when targeted sequencing failed to detect somatic mutations identified in the matched primary tumor at variant allele frequencies (VAFs) greater than 5%, suggesting insufficient representation of tumor-derived cells within the culture. For cases lacking sufficient matched tumor tissue, validation was based on multiple orthogonal features, including histopathologic assessment, detection of breast cancer-associated mutations, and WGS copy number alteration profiles. Organoids with relatively low CNV burden were retained when they harbored tumor-associated mutations consistent with the originating tumor. Cultures lacking both tumor-associated CNV changes and breast cancer-associated somatic variants were classified as non-tumor outgrowths and excluded from tumor-response analyses.

RNA sequencing and analysis

Bulk RNA-sequencing and analysis of TNBC TORG breast cancer organoids were performed at Novogene. In short, after sample quality control, mRNA libraries were prepared using poly(A) enrichment of the mRNA. The library was checked with Qubit and real-time PCR for quantification and a bioanalyzer for size distribution detection. Illumina NovaSeq 6000 PE150 or Illumina NovaSeq X Plus was used for high-throughput sequencing. All FASTQ files were processed in the same way. Raw sequencing reads were filtered to remove adapter-contaminated and low-quality sequences. Gene expression levels were quantified using RSEM,[92] with reads aligned to the human reference genome (hg38) with Bowtie2[76] and annotated using the GENCODE 49 gene set,[74] providing transcript abundance estimates for downstream analyses.

Merge of organoid data with TCGA

Expected gene counts from RSEM for TNBC TORG organoids were analyzed as follows. Per batch, genes (identified by Ensembl ID) with zero counts across all samples were removed. Ensembl IDs were mapped to Entrez IDs using the biomaRt package in R. Expression values were averaged for genes with duplicate Entrez IDs. Then, count data were log-transformed (log2(x+1)). Similarly, the raw counts of HUB organoids were log-transofmred and processed.

To analyze breast cancer subtypes and I-SPY gene expression biomarker signatures in tumor organoid data, we quantile-normalized the log-transformed organoid bulk RNA-sequencing data to a larger dataset of TCGA breast cancer tissues to enable analysis and interpretation of the organoid data in a larger, more robust context.[73] Expression and clinical data from breast cancer samples in TCGA were downloaded from the PanCanAtlas Publications Supplemental Data (''EBPlusPlusAdjustPANCAN_IlluminaHiSeq_RNASeqV2.geneExp.tsv'' and ''TCGA-CDR-SupplementalTableS1.xlsx'').[102] Similar to organoid expression data, TCGA expression data were log-transformed (log2(x+1)). Gene IDs in both TCGA and organoid datasets were converted from Ensembl ID to Entrez ID using biomaRt[75] in R, and the organoid expression data were quantile normalized using the TCGA dataset as the target (preprocessCore package in R[90]). Then, the organoid and TCGA data were merged and Entrez ID was converted to Gene Symbol, resulting in a combined matrix of 17733 genes and 189 samples for TORG-TCGA (14 TNBC TORG and 175 TNBC TCGA samples) and a combined matrix of 17896 genes and 1107 samples for HUB-TCGA (26 HUB and 1081 TCGA samples). Quality controls were performed to confirm the successful merge of the data, including principal component analysis (PCA) of the tumor organoids together with all TCGA breast cancer samples based on the expression of the PAM50 genes and distribution plots gene expression. PCA was created using DESeq2[78] and ggfortify[83] in R.

The merged organoid-TCGA datasets were then subtyped by PAM50-subtype (genefu package in R[80]) and two TNBC classification schemas (Burstein/Brown triple-negative classifications[103] and TNBC-type[104,105]). To evaluate the match between HUB PAM50 classifications by the authors of the original HUB biobank publication and our PAM50 subtype classification of the HUB organoids, we defined ''close match'' as an exact match in final call or, in case of non-matching calls, overlapping major subtype components in the distribution of subtype probability scores between the HUB and our analyses. The Burstein/Brown TN classifications (LAR, MES, BLIS, BLIA) were identified by (1) quantile transforming over their predictor genes; (2) calculating Euclidean distance to the 4 published centroids; and (3) assigning class based on the closest (minimal distance) centroid.[5] TNBC-type classifications (MSL, M, LAR, IM, BL2, BL1) were identified by uploading the triple-negative samples of the merged TORG-TCGA expression dataset to the online calculator (https://cbc.app.vumc.org/tnbc/). When analyzing TNBC-specific gene-expression-based subtype classifications, we found that 11/175 (6.3%) of the TNBC TCGA samples were rejected by the TNBC-type algorithm based on the expression of an ER-positive-like profile.

Analysis of 100 biomarker gene sets

Hallmark gene set scores (50 gene sets)[106] were calculated by gsva (msigdbr[88] and GSVA[86] packages in R) and qualifying I-SPY2 biomarkers were scored as described by Wolf et al. (2022)[5] in the merged organoid-TCGA tissue datasets. Moreover, a set of

exploratory, partly immune, biomarkers from the I-SPY2 compendium and other published gene signatures were analyzed.[47,107–114] Gene lists and references are provided in Table S9. One biomarker that was evaluated in the HUB dataset could not be evaluated in the TORG dataset due to insufficient PCDC1 expression, therefore 99 biomarkers were included in the final TORG analysis. After calculating absolute gene signature scores and Z-scoring the results, the organoid and TCGA samples were split by receptor subtype, and relative expression values between 0 and 1 ('p-th quantiles') were calculated across each subset, allowing the interpretation of the relative expression level (ranking) of a signature in one sample compared with other samples of the same receptor subtype, both organoids and TCGA tissues.

Sensitivity testing to cisplatin-veliparib

As an initial screen, we performed a "rapid response assay" across 7 organoid lines using a single-dose treatment approach after limited organoid expansion. A biologically informative screening dose was selected *a priori* to distinguish relative responder versus non-responder phenotypes across multiple organoid lines while preserving assay feasibility in limited patient-derived material. This assay was designed to provide a rapid functional assessment of directional treatment response across multiple patient-derived organoids, rather than to establish definitive quantitative sensitivity thresholds.

Organoids were grown in 50 μL 100% BME gel domes in 24-well plates supplemented with organoid medium and grown to at least medium confluency. The well with organoids was collected and digested into small clumps using TrypLE for 15 min, filtered through a 100 μm filter, and resuspended in organoid medium containing 6–10% BME. 90 μL of organoid suspension was dispensed into each well for a total of 4–6 wells. One to two days after plating, organoids were exposed to a fixed combination dose of 10 μM cisplatin (SigmaAldrich, Cat. No. 232120-50MG) in 0.9% NaCl plus 20 μM veliparib (SelleckChem, Cat. No. S1004) in DMSO or vehicle (DMSO) control (2–3 technical replicates per condition) in media containing CellTox at a 1:1000 final concentration (Promega, Cat. No G8743) for 72–120 h, depending on organoid growth rate. Using live-cell imaging, we assessed relative cell death in the cisplatin + veliparib wells compared with the control wells, measured as fluorescent (CellTox) object count per image as a marker of cell death, and relative organoid growth, assessed by Brightfield imaging, in response to veliparib-cisplatin compared with control at the assay endpoint.

In-depth *in vitro* drug response evaluation to veliparib and cisplatin (monotherapies and combination therapy) was tested in every organoid culture that had sufficient growth to perform the drug experiment at least in triplicate (biological replicates), with at least 3 technical replicates in each biological replicate.

Organoids were similarly processed from gel domes in 24-well plates to suspension (90 μL of organoid suspension/well) in a clear-bottom 96-well plate (Corning, Cat. No. 3610). For these organoids, one confluent well yielded 10–20 wells in a 96-well plate. On day 2, we added 30 μl of medium containing CellTox a 1:1000 final concentration as a fluorescent marker of cell death by live-cell imaging, and either DMSO (negative control), 10 μM cisplatin in 0.9% NaCl, 20 μM veliparib in DMSO, or 10 μM cisplatin in 0.9% NaCl +20 μM veliparib in DMSO, in technical triplicate. The final DMSO concentration in the wells was kept under 1%. Automated live-cell imaging captured each well at 6-h intervals using both brightfield and fluorescent imaging. For fast-growing organoids, drug experiments were stopped at T = 72 h, and cell viability was assessed using CellTiter-Glo. Experiments with slower-growing organoids were stopped at T = 120 h and cell viability was assessed using CellTiter-Glo.

In TORG40, the most robust growing organoid, which was also the organoid with the highest predicted resistance to veliparib + platinum chemotherapy (VP), and TORG146, a robust predicted VP-sensitive culture, we performed a 5-point dose-response curve to cisplatin (in three biological replicates) to identify the IC10 and IC50, to guide dose selection for the platinum backbone therapy in later combination treatment testing in the organoids. After plating as described previously, 30 μL of medium containing cisplatin in increasing doses between 4 and 400 μM was added to each of the wells in triplicate (final concentrations of cisplatin in the well: between 1 and 100 μM). After 72 h in TORG40, and 120 h in TORG146, cell viability was assessed using CellTiter-Glo. Plates were read in a luminometer.

Small molecule drug screen

After identifying a predicted and *in vitro* confirmed resistant organoid culture to VP, we expanded this culture to perform a high-throughput drug screen using the Ludwig Selleck Anti-Cancer Library (https://iccb.med.harvard.edu/ludwig-selleck-anti-cancer-library) containing 386 characterized bioactive compounds with validated targets against cancer-related proteins at the ICCB-Longwood Screening Facility at Harvard Medical School (HMS).

TORG40, the organoid with the highest predicted and *in vitro* confirmed resistance to VP was used for the initial screen. Prior to screening, TORG40 was grown in 100% BME gel domes in 24-well plates supplemented with fully defined organoid medium. Two days prior to library addition, the domes of gel containing organoids were digested into small clumps using TrypLE, filtered through a 100 μm filter, and resuspended in organoid medium containing 6% BME gel. Every 6 confluent wells of a 24-well plate yielded one 384-well plate. The organoid suspension was then plated into 384-well plates (Corning 3570) with 30 μL/well using the ThermoFisher Multidrop Combi Reagent Dispenser.

On the day of screening, 100 nL of each library compound in DMSO was pin-transferred into the 384-well plate. Each compound was plated in a 9-step logarithmic concentration range from 25 μM down to 0.25 nM in the well. For each drug compound at every concentration, there were four replicates. After adding the library compounds, two replicates received 10 μL/well of organoid medium, and two replicates received 10 μL/well of organoid medium containing cisplatin for a final volume of 40 μL/well and a cisplatin concentration of 5 μM. Columns 1, 2, and 23 of each plate were given 10 μL of 1 mg/mL aqueous cisplatin as a positive control, and

column 24 (negative control) was given 10 μL of organoid medium with DMSO to normalize the amount of vehicle (DMSO) across the wells (<1% DMSO/well). Column 24 in the +cisplatin plates was given organoid medium with DMSO and cisplatin to achieve a final cisplatin dose of 5 μM. Plates were stacked 4 high in a high-humidity incubator and left for three days. The library pin transfers were split into four library plates at a time due to the large number of assay plates. On day 3 after pin transfer, cells were lysed with 20 μL/well CellTiter-Glo (1:2 ratio to medium volume, Promega, Cat. No. G7572), shaken at room temperature for 15 min, and viability was measured by luminescence using a PerkinElmer EnVision.

To calculate relative cell viability, crosstalk-corrected luminescence values were normalized to the appropriate negative controls. Combination treatment wells (containing cisplatin) were normalized to negative controls containing 5 μM cisplatin to control for the moderate killing by cisplatin as a monotherapy (~5%). Using the average relative cell count of the duplicates for each condition at different concentrations (concentrations on a logarithmic scale), dose-response curves were generated using GraphPad Prism, and the area-under-the-dose-response-curve (AUC) was calculated using base R code. GRmetrics[85] (R) was used to calculate metrics (e.g., IC50) for drug response data. Z′ factors were calculated for each of the 64 plates in the drug screen and summarized with the median and interquartile range.[51]

The ratio of AUCs between the condition with cisplatin and without cisplatin was used to rank the compounds and select the top 5% of compounds in the screen with the largest reduction in AUC with the addition of 5 μM cisplatin ($n = 19$). We then reviewed only those compounds of which the IC50 value of the combination treatment with cisplatin was among the smallest 20% in the compound screen ($n = 17/19$), and chose a selection of 8 compounds, with various mechanisms of action (HDAC inhibitors (epigenetics), Bcl-2 family inhibitors (apoptosis), an inhibitor of DNA synthesis (DNA damage repair), an IAP inhibitor (apoptosis), and an aurora kinase inhibitor (cell cycle regulation) with a maximum of 3 compounds per drug target to validate in a secondary assay.

Validation of top combination hits using a secondary, imaging-based assay

TORG40 was plated in suspension (90 μL/well) in a 96-well plate as described above. Two days after plating, drugs/controls and a fluorescent cytotoxicity dye, CellTox at a 1:1000 final concentration, were added in 30 μL of organoid medium. Each experimental drug (Trichostatin A, Vorinostat, BV6, JNJ26481585, ABT-263, Obatoclax, Clofarabine, and AT9283) was tested at 3 concentrations (5 μM, 1 μM, and 0.2 μM), both with and without a fixed dose (5 μM) of cisplatin. The drugs were plated in at least 2 technical replicates per concentration. In addition to the experimental wells, a negative control (DMSO), a negative control with 5 μM cisplatin, a positive control with ultra-high dose, 100 μM cisplatin and a positive control with lysis buffer solution (Promega, Cat. No. G8743) were included. The drugged organoids were incubated for 72 h in the Incucyte live-cell analysis system (Sartorius, Model No. S3-C2) with images automatically taken of each well every 6 h. The system automatically selects one focus position for imaging, which cannot be changed manually. After 72 h, cells were lysed with CellTiter-Glo (Promega, Cat. No. G7572) at a 1:2 ratio, and luminescence readings were taken on the BioTek Synergy HT after leaving the plate on a shaker for 30 min.

We evaluated multiple outcome measures acquired in Incucyte, including fluorescent and brightfield object count, fluorescent and brightfield area, fluorescence intensity, fluorescent object count normalized to total organoid area by brightfield, and found fluorescent object count relative to baseline (T = 0 h) or the first timepoint (T = 6 h), depending on which was the first in-focus fluorescence recording, to be the most robust outcome measure. The total fluorescent area did not always correlate with the number of fluorescent objects/organoids, likely due to differences in focus position, which is automatically selected by the system. This is why fluorescent object count was a more stable outcome than fluorescent area. We confirmed an additive effect of cisplatin with all eight top compounds at a minimum of one of the concentration levels tested as quantified by an increase in total fluorescent object count in the combination treatment with cisplatin versus the monotherapy, along with matching morphological changes by Brightfield imaging. Plots were made using GraphPad Prism and ggplot2 in R.

Validation of top hits in other intermediate/resistant organoid cultures

TORG40, TORG58, and TORG139 were plated in suspension (90 μL/well) in a 96-well plate as described above. Similarly, two days after plating, drugs, negative (DMSO) and ultra-high dose positive (100 μM cisplatin and/or 5 μM ABT-263) controls, and CellTox at a 1:1000 final concentration (Promega, Cat. No. G8743), were added in 30 μL of organoid medium. ABT-263 (SelleckChem, Cat. No. S1001) was tested at 0.3 μM with and without 5 μM cisplatin in three biological replicates, with 3 technical replicates in each biological replicate. Separately, ganetespib (Medchemexpress, Cat. No. HY-15205-10MG) was tested as a monotherapy in a 5-point dose-response curve ranging from 0.001 μM to 10 μM in the same fashion in at least two biological replicates, with 3 technical replicates in each biological replicate. The drugged organoids were incubated for variable times ranging from 72 h to 120 h, depending on individual growth rates, in the Incucyte, with images automatically taken of each well every 6 h. After incubation, cells were lysed with CellTiter-Glo (Promega, Cat. No. G7572) at a 1:2 ratio, and luminescence readings were taken on the BioTek Synergy HT after leaving the plate on a shaker for 30 min. Plots were made using GraphPad Prism and ggplot2 in R.

Synergy testing

A formal synergy experiment was performed with ABT-263 for TORG40, TORG58, and TORG139 by plating the organoids in suspension (90 μL/well) in a 96-well plate as described above. Similarly, two days after plating, drugs and controls as described above, and CellTox at a 1:1000 final concentration (Promega, Cat. No. G8743) were added in 30 μL of organoid medium. Cisplatin was added at 5 increasing doses (0 μM, 1 μM, 5 μM, 10 μM, 50 μM, and 100 μM) along the vertical axis and 4 increasing doses of ABT-263 (0 μM,

0.01 μM, 0.1 μM, 0.5 μM, and 1 μM) along the horizontal axis of a 96-well plate. Each dose combination was tested in technical duplicates within each plate, with two biological replicates per organoid. The drugged organoids were incubated for 72 h in the Incucyte, with images automatically taken of each well every 6 h. After incubation, cells were lysed with CellTiter-Glo (Promega, Cat. No. G7572) at a 1:2 ratio, and luminescence readings were taken on the BioTek Synergy HT after leaving the plate on a shaker for 30 min.

The synergyfinder package in R[93] was used for analyzing these multiple drug combination datasets and provided implementations for 4 popular synergy scoring models, including the Bliss synergy score. Summary synergy scores can be interpreted as the average excess response due to drug interactions (i.e., synergy score of 15 corresponds to 15% of response beyond expectation). Since combination synergy is highly context-specific, there are no defined thresholds for good synergy scores. One way to give meaning to synergy scores is to interpret a synergy score of less than −10 as the interaction between two drugs likely to be antagonistic, from −10 to 10 as the interaction between two drugs likely to be additive, and larger than 10 as the interaction between two drugs likely to be synergistic.[52] Synergy plots show the results of one biological replicate for each organoid culture.

Distant-recurrence free survival analysis in I-SPY2 clinical trial data

In the I-SPY2 trial, patients with high-risk early-stage HER2-negative breast cancer were eligible for randomization to the HSP90 inhibitor ganetespib in combination with standard chemotherapy between October 2014 and October 2015,[53] which was one of the 10 treatment arms in the "I-SPY2-990 dataset". In the ganetespib arm (150 mg/m^2 ganetespib every 3 weeks with weekly paclitaxel over 12 weeks, followed by four cycles of intravenous 60 mg/m^2 doxorubicin plus 600 mg/m^2 cyclophosphamide (AC)), 38 patients had HER2-/Immune-/DRD-tumors. The standard neoadjuvant chemotherapy arm (weekly paclitaxel over 12 weeks, followed by AC) included 186 patients with HER2-/Immune-/DRD-tumors. The primary endpoint was pathologic complete response (pCR), defined as the absence of invasive disease in breast and regional nodes (ypT0/is and ypN0) at the time of surgery. Distant recurrence-free survival (DRFS), defined as time to distant recurrence or death from any cause, was evaluated as a secondary endpoint. Kaplan-Meier survival curves were generated for each arm, along with hazard ratios obtained through Cox proportional hazards modeling. Data collection was cut off in September 2025. For the evaluation of DRFS, 37 of 38 patients in the ganetespib arm and 185 of 186 in the control arm had follow-up data, with a median follow-up time of 5.4 years.

QUANTIFICATION AND STATISTICAL ANALYSIS

Statistical methods are outlined in the respective figure legends where applicable. Statistical analyses were performed in R. Comparisons of gene (signature) expression levels in subsets of patients and organoids were made using Wilcoxon rank sum tests, with *p*-values adjusted for multiple hypothesis testing using the Benjamini-Hochberg (BH) method using the multtest package in R. P-values <0.05 were considered statistically significant. Pearson correlation analysis was used to test correlations between expression scores of selected biomarkers in organoids and clinical features of the corresponding tumors. Drug response (cell viability) outcomes using CellTiter-Glo are reported as the mean of 2–3 biological replicates ±standard error of the mean (SEM). Drug response outcomes from CellTox (live-cell imaging) are shown as the mean of technical replicates ±SEM within a single experiment. Data are representative of 2–3 independent biological replicates. Detailed information on the number of replicates is indicated in the STAR Methods section of each experiment. After manual adjustments to exclude background fluorescence, setting the radius and the fluorescence threshold, the fluorescent object count normalized to baseline (T = 0 h) or the first timepoint (T = 6 h), depending on which was the first in-focus fluorescence recording, was extracted from the Incucyte integrated analysis software and subsequently visualized in R using the ggplot2 package. Statistical testing for all cell viability outcomes (by CellTiter-Glo) and fluorescence outcomes (by CellTox) was done using the Welch's *t* test for comparing two groups or a Welch's ANOVA, followed by the Games–Howell test for comparing multiple treatment conditions. Statistically significant results are indicated by exact *p* values or asterisks in the figures, which are specified in the figure legends. Distant recurrence–free survival (DRFS) was analyzed using the Kaplan–Meier method, and survival curves were compared between groups using the log rank test. Cox proportional hazards regression models were used to estimate hazard ratios (HRs) and corresponding 95% confidence intervals (CIs). All heatmaps were created using pheatmap[89] in R. Scatter and correlation plots were created using GGally[82] and ggplot2[84] in R. Graphics (Figures 1A, 2A, 3A, and 6F) were created in BioRender.

ADDITIONAL RESOURCES

The I-SPY trial is registered under ClinicalTrials.gov ID NCT01042379 (https://clinicaltrials.gov/study/NCT01042379).

Cell Reports Medicine, Volume *7*

Supplemental information

Biomarker-guided responses in patient-derived organoids predict effective therapies in breast cancer

Tam Binh V. Bui, Denise M. Wolf, Michael C. Bruck, Kaitlin Moore, Jessica Lien, Sarah D. W. Choi, Shruti Warhadpande, Amirabbas Parizadeh, Deborah Dillon, Beth Overmoyer, Filipa Lynce, Lamorna Brown-Swigart, Gillian L. Hirst, Christina Yau, I-SPY2 Investigators, Isaac J. Nijman, Boudewijn M.T. Burgering, Isaac S. Harris, Laura J. Esserman, Laura J. van `t Veer, and Jennifer M. Rosenbluth

Figure S1

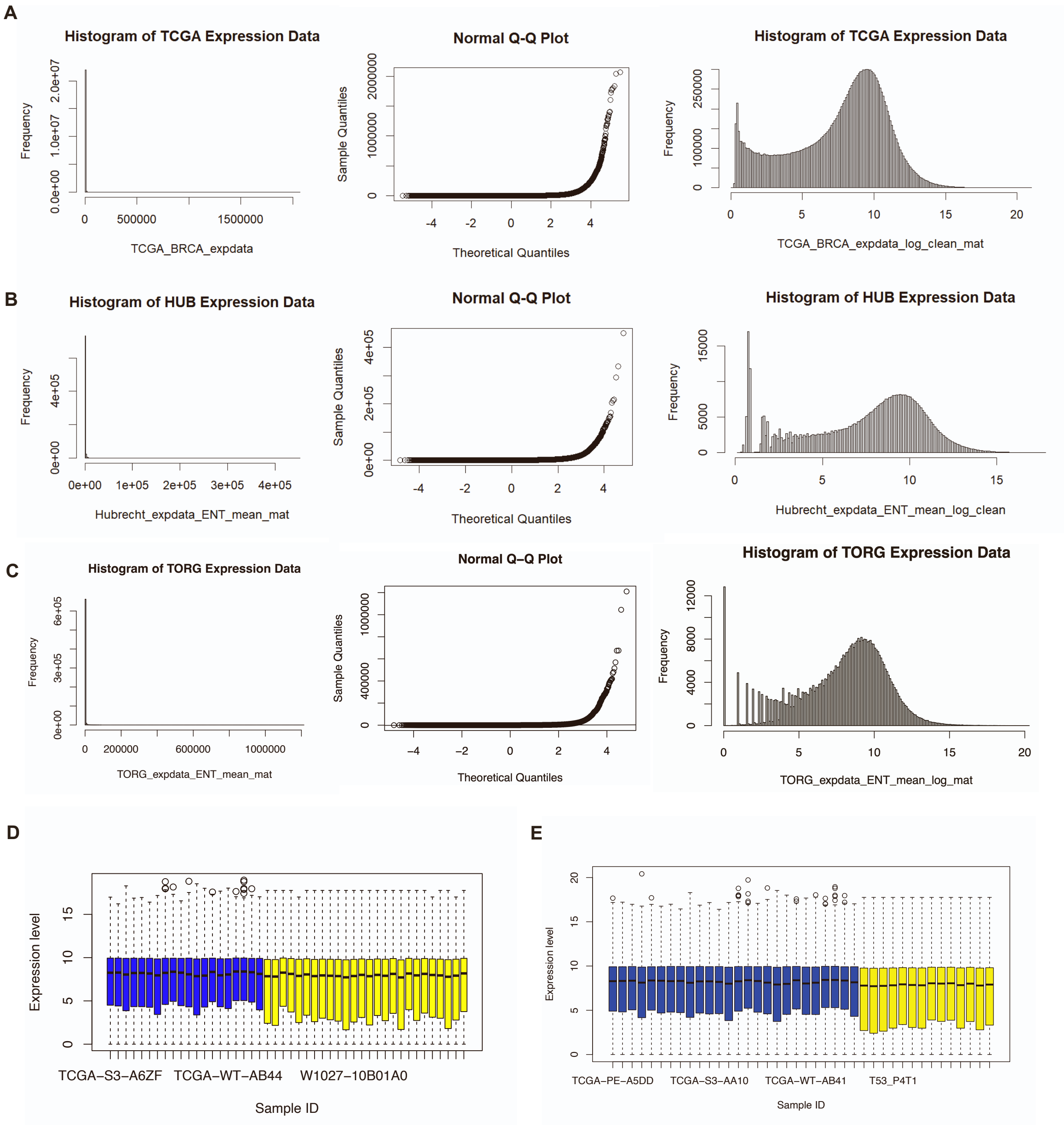
A
Histogram of TCGA Expression Data
Frequency
TCGA_BRCA_expdata
Normal Q-Q Plot
Sample Quantiles
Theoretical Quantiles
Histogram of TCGA Expression Data
TCGA_BRCA_expdata_log_clean_mat
B
Histogram of HUB Expression Data
Hubrecht_expdata_ENT_mean_mat
Normal Q-Q Plot
Histogram of HUB Expression Data
Hubrecht_expdata_ENT_mean_log_clean
C
Histogram of TORG Expression Data
TORG_expdata_ENT_mean_mat
Normal Q–Q Plot
Histogram of TORG Expression Data
TORG_expdata_ENT_mean_log_mat
D
Expression level
TCGA–S3–A6ZF TCGA–WT–AB44 W1027–10B01A0
Sample ID
E
Expression level
TCGA–PE–A5DD TCGA–S3–AA10 TCGA–WT–AB41 T53_P4T1
Sample ID

Figure S1. Quality controls for the merge/integration of organoid bulk RNA-Seq and TCGA tissue bulk RNA-Seq data. Q-Q plot = Quantile-Quantile plot. TCGA = The Cancer Genome Atlas. Related to Figure 1 and Figure 3.

A. Gene expression data from TCGA published breast cancer tissues. Left to right: histogram of data before log-transformation, Q-Q plot of data before log-transformation, and histogram of log-transformed expression data.

B. Gene expression data from HUB biobank (Hubrecht Institute) organoids. Left to right: histogram of data before log-transformation, Q-Q plot of data before log-transformation, and histogram of log-transformed expression data.

C. Gene expression data from TNBC TORG (UCSF triple-negative breast cancer organoids). Left to right: histogram of data before log-transformation, Q-Q plot of data before log-transformation, and histogram of log-transformed expression data.

D. Boxplots showing expression levels for a representative subset of the TCGA dataset (blue bars), and all HUB organoid samples (n=26; yellow bars) after quantile-normalization of organoid data to TCGA data and merge of the data. The boxplots illustrate comparable expression levels within samples of both datasets after transformation of the data.

E. Boxplots showing expression levels for a representative subset of the TCGA dataset (blue bars), and all TORG TNBC organoid samples (n=14; yellow bars) after quantile-normalization of organoid data to TCGA data and merge of the data. The boxplots illustrate comparable expression levels within samples of both datasets after transformation of the data.

Figure S2

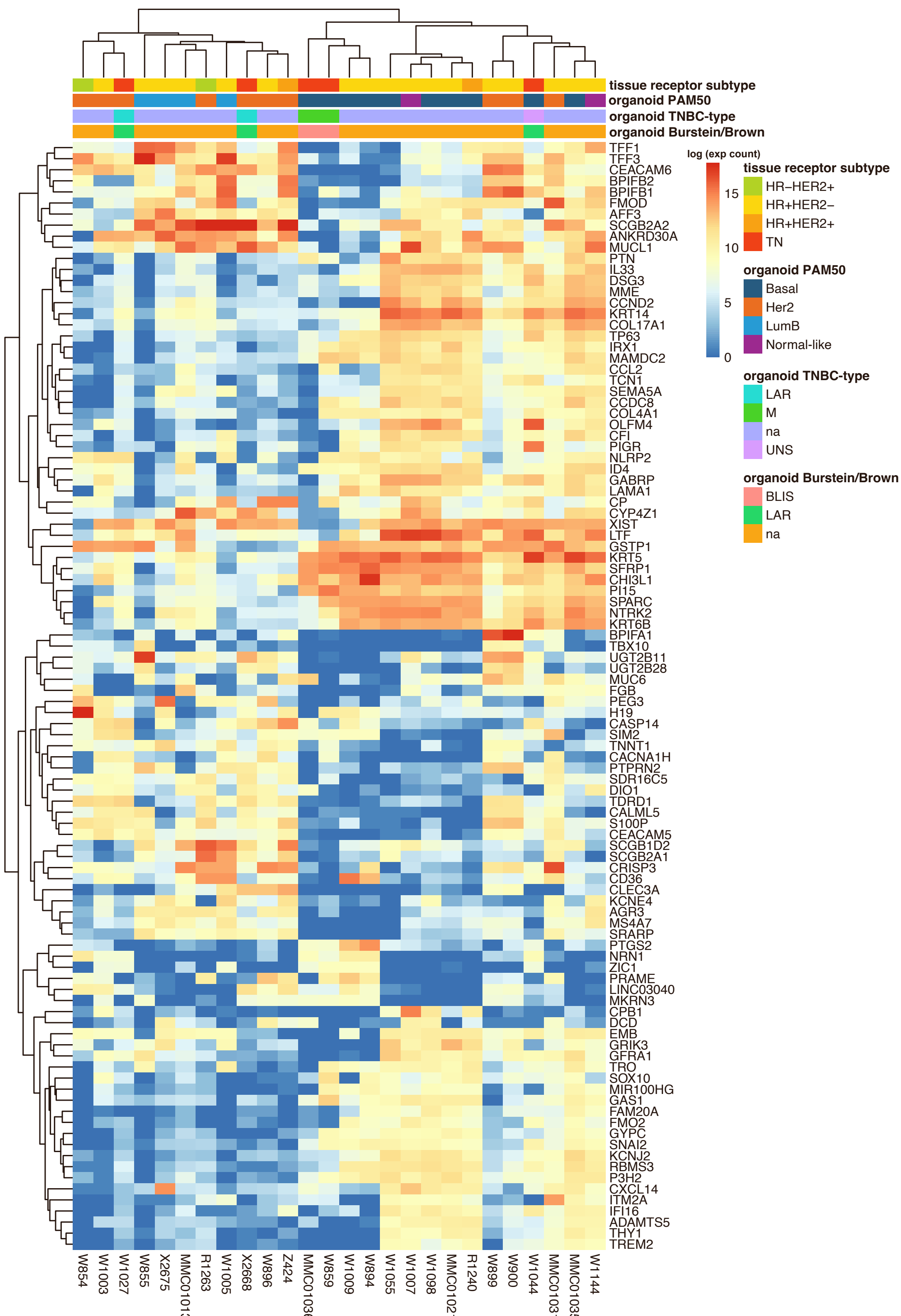
tissue receptor subtype
organoid PAM50
organoid TNBC-type
organoid Burstein/Brown
log (exp count)
15
10
5
0
tissue receptor subtype
HR−HER2+
HR+HER2−
HR+HER2+
TN
organoid PAM50
Basal
Her2
LumB
Normal-like
organoid TNBC-type
LAR
M
na
UNS
organoid Burstein/Brown
BLIS
LAR
na
TFF1
TFF3
CEACAM6
BPIFB2
BPIFB1
FMOD
AFF3
SCGB2A2
ANKRD30A
MUCL1
PTN
IL33
DSG3
MME
CCND2
KRT14
COL17A1
TP63
IRX1
MAMDC2
CCL2
TCN1
SEMA5A
CCDC8
COL4A1
OLFM4
CFI
PIGR
NLRP2
ID4
GABRP
LAMA1
CP
CYP4Z1
XIST
LTF
GSTP1
KRT5
SFRP1
CHI3L1
PI15
SPARC
NTRK2
KRT6B
BPIFA1
TBX10
UGT2B11
UGT2B28
MUC6
FGB
PEG3
H19
CASP14
SIM2
TNNT1
CACNA1H
PTPRN2
SDR16C5
DIO1
TDRD1
CALML5
S100P
CEACAM5
SCGB1D2
SCGB2A1
CRISP3
CD36
CLEC3A
KCNE4
AGR3
MS4A7
SRARP
PTGS2
NRN1
ZIC1
PRAME
LINC03040
MKRN3
CPB1
DCD
EMB
GRIK3
GFRA1
TRO
SOX10
MIR100HG
GAS1
FAM20A
FMO2
GYPC
SNAI2
KCNJ2
RBMS3
P3H2
CXCL14
ITM2A
IFI16
ADAMTS5
THY1
TREM2
W854
W1003
W1027
W855
X2675
MMC01013
R1263
W1005
X2668
W896
Z424
MMC01036
W859
W1009
W894
W1055
W1007
W1098
MMC01021
R1240
W899
W900
W1044
MMC01031
MMC01035
W1144

Figure S2. Unsupervised clustering of HUB biobank organoids based on the top 100 most differentially expressed genes. Annotations for each of the indicated subtype classification schemas are shown. BLIS = basal-like immunosuppressed. Exp count = expected count (RSEM). HR = hormone receptor. LAR = luminal androgen receptor. LumB = luminal B. M = mesenchymal. na = not applicable (sample is not triple-negative). TN = triple-negative. UNS = a subtype could not be selected by the algorithm. Related to Figure 1.

Figure S3

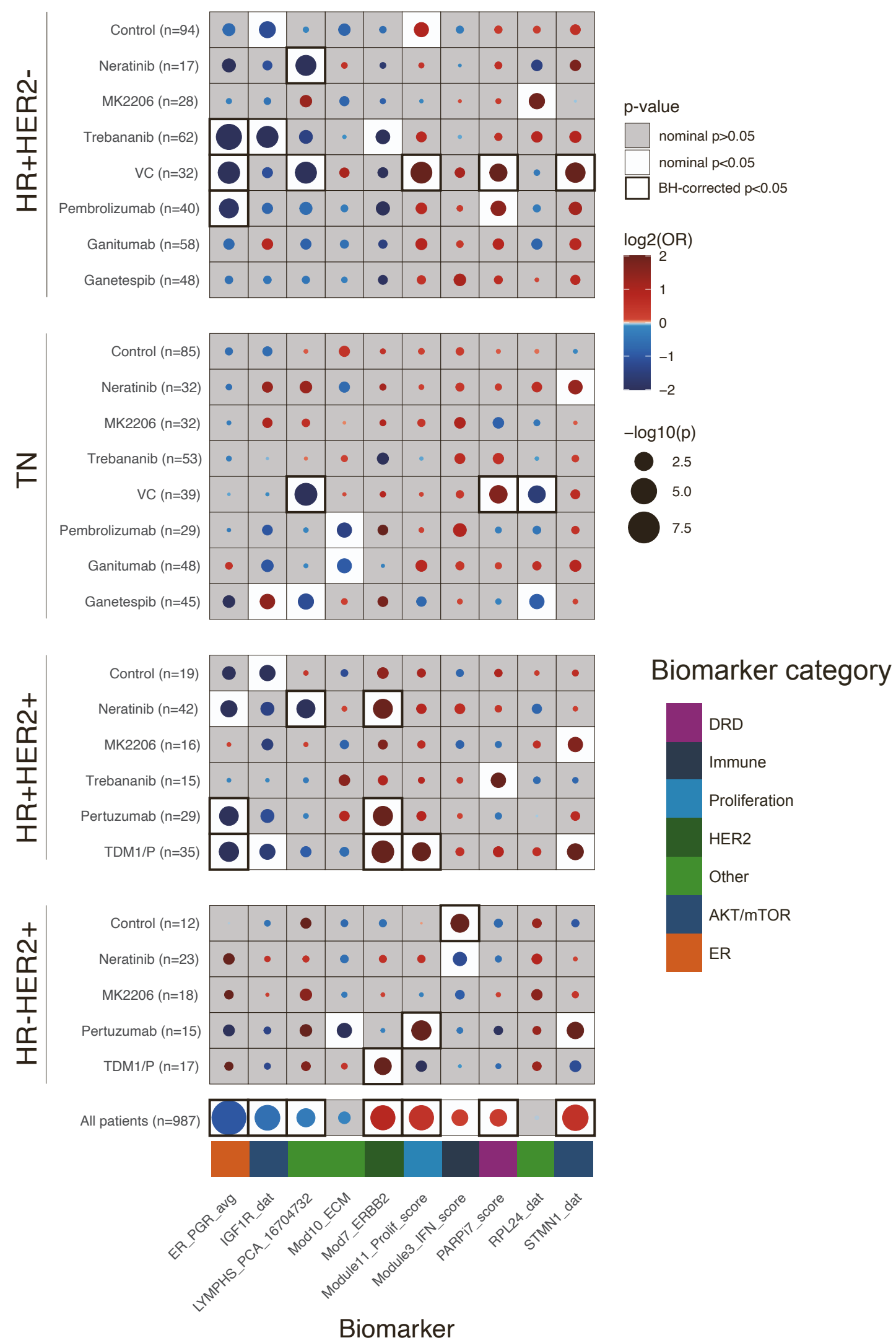

Figure S3. Results of pathologic complete response (pCR) association analysis in the I-SPY2 clinical trial are shown for each organoid-evaluable biomarker in each treatment arm by receptor subtype, based on gene expression and clinical data from the trial (Wolf et al., 2022) (right panel). Each row is a treatment arm. Dot color (red/blue) and intensity reflect the direction and strength of association with pCR, while dot size corresponds to the significance of the association. White background boxes indicate a nominal p-value < 0.05, and black border indicates False Discovery Rate (FDR) p-value <0.05 (Benjamini-Hochberg correction, BH). HR = hormone receptor. OR = odds ratio. p = p-value. TDM1/P = ado-trastuzumab emtansine plus pertuzumab (T-DM1/P). TN = triple negative. VC = veliparib-carboplatin. Related to Figure 2.

Figure S4

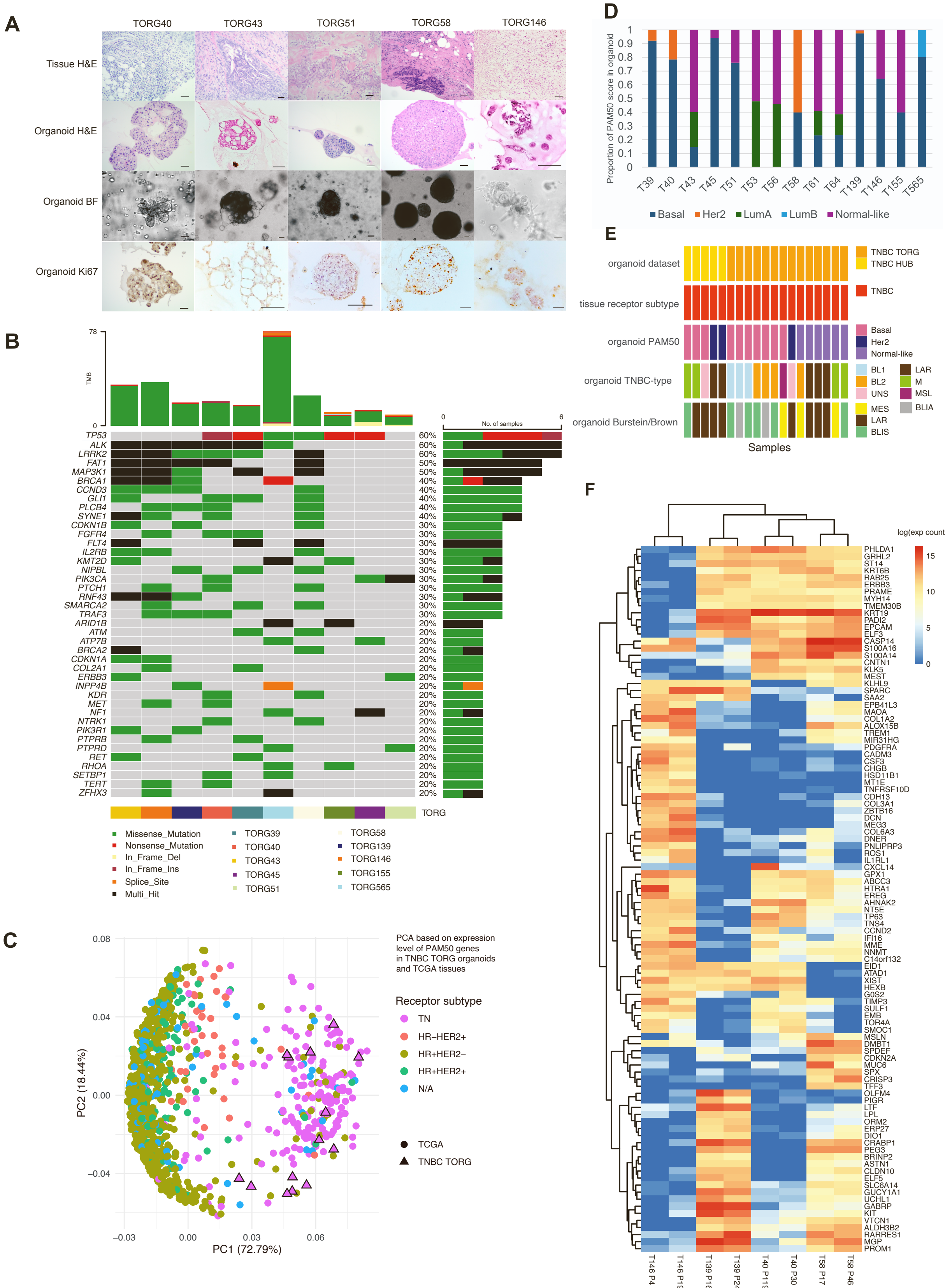
A
TORG40
TORG43
TORG51
TORG58
TORG146
Tissue H&E
Organoid H&E
Organoid BF
Organoid Ki67
B
TMB
No. of samples
Missense_Mutation
Nonsense_Mutation
In_Frame_Del
In_Frame_Ins
Splice_Site
Multi_Hit
TORG39
TORG40
TORG43
TORG45
TORG51
TORG58
TORG139
TORG146
TORG155
TORG565
C
PCA based on expression level of PAM50 genes in TNBC TORG organoids and TCGA tissues
Receptor subtype
TN
HR−HER2+
HR+HER2−
HR+HER2+
N/A
TCGA
TNBC TORG
PC1 (72.79%)
PC2 (18.44%)
D
Proportion of PAM50 score in organoid
Basal
Her2
LumA
LumB
Normal-like
E
organoid dataset
tissue receptor subtype
organoid PAM50
organoid TNBC-type
organoid Burstein/Brown
Samples
TNBC TORG
TNBC HUB
TNBC
F
log(exp count)

Figure S4. Validation of sensitivity/resistance biomarkers and cancer gene sets in the TNBC TORG organoid bank. BL1/BL2 = basal-like 1/2. BLIA = basal-like immune-activated. BLIS = basal-like immunosuppressed. Exp count = expected count (RSEM). HR = hormone receptor. LAR = luminal androgen receptor. LumA = luminal A. LumB = luminal B. M/MES = mesenchymal. MSL = Mesenchymal Stem-Like. N/A = not available. 'P' = passage number. TMB = tumor mutational burden. TN = triple negative. TNBC = triple negative breast cancer. UNS = a subtype could not be selected by the algorithm. Related to Figure 3.

A. Representative organoid cultures show diverse morphologies and phenotypes in culture, including botryoid, solid, cystic, and metaplastic/spindle cell phenotypes. Histology of the tumor tissue and embedded derived organoids (stained by Hematoxylin and Eosin; H&E), a brightfield (BF) image of the organoids in culture, and results of immunohistochemical staining for Ki67 are shown. Ki67 expression was evident in all cases that underwent additional immunohistochemical staining (n=7) with a range of expression levels, consistent with clinical variation in high-grade features. Scale bar = 100 μm.

B. Summary plot showing single-nucleotide variants (SNVs) and insertions/deletions in a representative subset of the tumor organoids (n=10), revealing the presence of mutations in breast cancer-associated genes across the biobank. Group B organoids (Figure 3D) generally did not have as many copy number alterations, but did exhibit mutations in cancer-associated genes, including TP53 and DIS3, and expressed Hallmark gene signatures, including higher epithelial mesenchymal transition and estrogen/androgen response signatures compared with organoids in group A (BH-corrected p-value <0.05) (Supplemental Table S5).

C. Principal component analysis (PCA) showing that organoids (triangles) cluster with The Cancer Genome Atlas (TCGA) tissue samples (circles) based on expression levels of PAM50 genes. Colors indicate breast cancer receptor subtypes. Axes include the percentage of variance explained by each principal component (PC).

D. Distribution of PAM50 score components for each organoid in the TNBC TORG biobank, showing heterogeneity in each case, with the highest score resulting in the final subtype call.

E. Hubrecht Organoid Technology (HUB) TNBC and TNBC TORG organoids reflect diverse breast cancer subtypes, identified by both general (PAM50) and TNBC-specific (TNBC-type and Burstein/Brown subtypes) classifications, compared to the receptor subtype of the original tumor specimen. The most frequent TNBC-type classifications were basal-like subtypes BL1 and BL2 (37% combined) and Luminal Androgen Receptor subtype (LAR; 26%). Using Burstein/Brown subtypes, basal-like immunosuppressed (BLIS) was the most frequent subtype among the TNBC organoids (32%), with fewer basal-like immune-activated (BLIA) cases (11%), as expected for organoids.

F. Heatmap comparing the expression of the top 100 differentially expressed genes in earlier and later passage cultures from four organoid models. Hierarchical clustering demonstrated that specimens from the same organoid model clustered more closely with one another than with other models across passages, supporting the relative preservation of model-specific transcriptional features over time in culture.

Figure S5

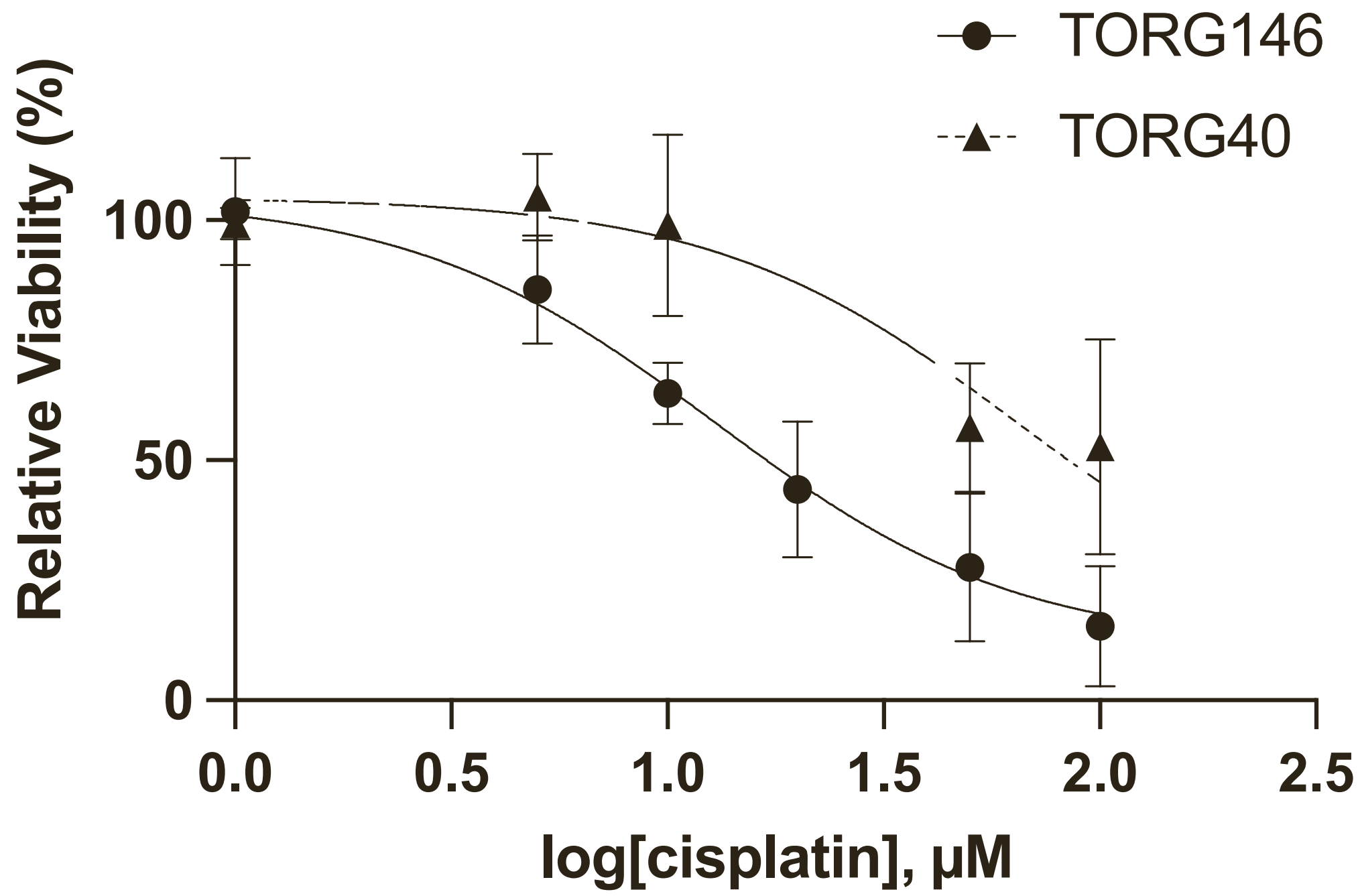

Figure S5. In vitro dose-response curve in TORG40 demonstrates resistance to cisplatin and dose-response curve in TORG146 demonstrates sensitivity to cisplatin. Relative cell viability (%) was determined at T=72 hours, and T=120 hours, respectively, using a luminescence-based cell viability assay (CellTiter-Glo). Data are expressed as the mean ± standard error of the mean (SEM) of three biological replicates. Related to Figure 5.

Table S1. Summary of clinical characteristics of HUB organoids included in the gene expression analysis (n=26). HR = hormone receptor. Related to Figure 1.

		**HR+HER2- (n=17)**	**Triple-Negative (n=5)**	**HR-HER2+ (n=2)**	**HR+HER2+ (n=2)**
**Grade**	1	0	1 (20%)	0	0
	2	11 (65%)	1 (20%)	0	2 (100%)
	3	6 (35%)	3 (60%)	2 (100%)	0
**Histological type**	Ductal carcinoma	14 (82%)	3 (60%)	2 (100%)	2 (100%)
	Lobular carcinoma	3 (18%)	0	0	0
	Other	0	2 (40%)	0	0
**Neoadjuvant therapy**	None	11 (65%)	4 (80%)	2 (100%)	1 (50%)
	Endocrine therapy	1 (6%)	0	0	0
	Chemotherapy	1 (6%)	0	0	1 (50%)
	Missing	4 (24%)	1 (20%)	0	0

Table S2. Details on multivariable logistic regression methods. Related to Figure 2.

A. Summary of significant multivariable logistic regression models to predict pathologic complete response (pCR) before refinement of the models. Using the I-SPY2-990 patient outcomes data (pCR) and the I-SPY2-990 patient tumor tissue expression level of these 9 organoid-evaluable biomarkers, we performed a multivariable logistic regression analysis across all receptor subgroup-treatment arm combinations. We used the step() function in R to perform forward selection and selected the resulting four models with three or more predictors. HR = hormone receptor. pCR = pathologic complete response. TN = triple negative.

Receptor subtype	Treatment arm	Model as identified by step() before refinement
HR+HER2-	Ganitumab (anti-IGF-1R)	pCR~Module11_Prolif_score + IGF1R_dat + LYMPHS_PCA_16704732 + Module3_IFN_score + STMN1_dat
HR+HER2+	Pertuzumab (anti-HER2)	pCR ~ Mod7_ERBB2 + PARPi7_score + ER_PGR_avg
HR+HER2+	T-DM1/Pertuzumab (anti-HER2)	pCR ~ Mod7_ERBB2 + Module11_Prolif_score + ER_PGR_avg + STMN1_dat
HR-HER2- (TN)	Veliparib/carboplatin (PARP inhibitor + platinum chemotherapy)	pCR ~ LYMPHS_PCA_16704732 + PARPi7_score + RPL24_dat

B. Final multivariable logistic regression models that predict treatment response in a specific receptor subtype after refinement. We manually reviewed the four models shown in A using summary(), and in case of any non-significant terms, we subsequently used drop1(model, test="Chisq") to remove variables that did not significantly contribute to the model (significant when p<0.05). Of these revised models, only three had more than one significant predictor. HR = hormone receptor. OR = odds ratio. P = predicted probability of pathologic complete response. pCR = pathologic complete response. TN = triple negative.

Receptor subtype	Treatment arm	OR predictor 1 (95% CI), p	OR predictor 2 (95% CI), p	OR predictor 3 (95% CI), p	Final model after refinement using drop1() including coefficients
HR+ HER2-	Ganitumab (anti-IGF-1R)	IGF1R_dat: 3.16 (1.03-9.75), p-value: 0.040	LYMPHS_PCA _16704732: 0.232 (0.0456-1.18), p-value: 0.072	n/a	log(P / (1 - P)) = -2.50 + 1.15* IGF1R_dat -1.46* LYMPHS_PCA_16704732
HR+ HER2+	Pertuzumab (anti-HER2)	Mod7_ERBB2: 3.90 (1.35-11.2), p-value: 0.00825	n/a	n/a	log(P / (1 - P)) = -1.84 + 1.36* Mod7_ERBB2
HR+ HER2+	T-DM1/ Pertuzumab (anti-HER2)	Mod7_ERBB2: 5.81 (1.07- 31.4), p-value: 0.036	Module11_ Prolif_score: 21.9 (1.32-364), p-value: 0.0252	ER_PGR_avg: 0.0421 (0.00147-1.21), p-value: 0.0542	log(P / (1 - P)) = 1.24 + 1.76* Mod7_ERBB2 + 3.09* Module11_Prolif_score - 3.17* ER_PGR_avg
HR- HER2- (TN)	Veliparib/ carboplatin (PARP inhibitor + platinum chemo-therapy)	LYMPHS_PCA_ 16704732: 0.0660 (0.00950- 0.458), p-value: 0.00445	PARPi7_ score: 4.82 (1.32-17.6), p-value: 0.0137	n/a	log(P / (1 - P)) = -1.95 - 2.72*LYMPHS_PCA_ 16704732 + 1.57*PARPi7_score

C. Receiver operating characteristic (ROC) curves showing the performance of the multivariable logistic regression model for predicting pCR with area under the curve (AUC) values for final models that predict treatment response in a specific receptor subtype after refinement using drop1() to exclude redundant predictors. AUC values closer to 1.0 indicate better performance of the model.

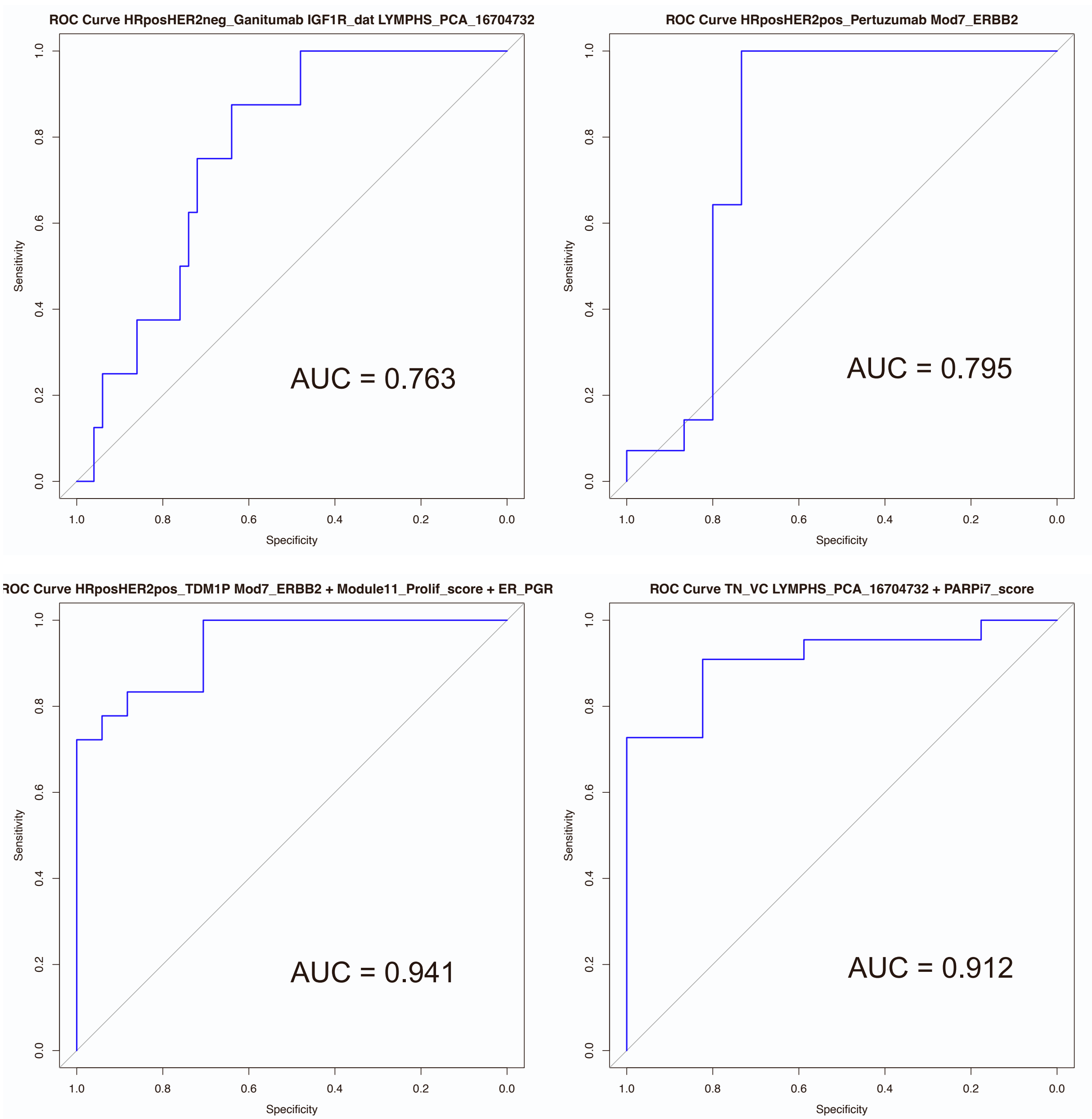

Supplement: Supplementary file 2 [file mmc9.pdf]
